# 5-AZA-dC induces epigenetic changes associated with modified glycosylation of secreted glycoproteins and increased EMT and migration in chemo-sensitive cancer cells

**DOI:** 10.1186/s13148-021-01015-7

**Published:** 2021-02-12

**Authors:** Gordon Greville, Esther Llop, Jane Howard, Stephen F. Madden, Antoinette S. Perry, Rosa Peracaula, Pauline M. Rudd, Amanda McCann, Radka Saldova

**Affiliations:** 1 GlycoScience Group, the National Institute for Bioprocessing, Research and Training (NIBRT), Fosters Avenue, Mount Merrion, Blackrock, Co Dublin Ireland; 2 College of Health and Agricultural Science (CHAS), UCD School of Medicine, University College Dublin (UCD), Belfield, Dublin 4, Ireland; 3 Biochemistry and Molecular Biology Unit, Department of Biology, University of Girona, Girona, Spain; 4 Girona Biomedical Research Institute (IDIBGI), Girona, Spain; 5 UCD Conway Institute of Biomolecular and Biomedical Research, University College Dublin (UCD), Belfield, Dublin 4, Ireland; 6 Data Science Centre, Royal College of Surgeons in Ireland (RCSI), Dublin 2, Ireland; 7 School of Biology and Environmental Science, University College Dublin (UCD), Belfield, Dublin 4, Ireland

**Keywords:** 5-AZA-2′-deoxycytidine, Ovarian, Breast, Cancer, Glycosylation

## Abstract

**Background:**

Glycosylation, one of the most fundamental post-translational modifications, is altered in cancer and is subject in part, to epigenetic regulation. As there are many epigenetic-targeted therapies currently in clinical trials for the treatment of a variety of cancers, it is important to understand the impact epi-therapeutics have on glycosylation.

**Results:**

Ovarian and triple negative breast cancer cells were treated with the DNA methyltransferase inhibitor, 5-AZA-2-deoxycytidine (5-AZA-dC). Branching and sialylation were increased on secreted *N*-glycans from chemo-sensitive/non-metastatic cell lines following treatment with 5-AZA-dC. These changes correlated with increased mRNA expression levels in *MGAT5* and *ST3GAL4* transcripts in ovarian cancer cell lines. Using siRNA transient knock down of GATA2 and GATA3 transcription factors, we show that these regulate the glycosyltransferases ST3GAL4 and MGAT5, respectively. Moreover, 5-AZA-dC-treated cells displayed an increase in migration, with a greater effect seen in chemo-sensitive cell lines. Western blots showed an increase in apoptotic and senescence (p21) markers in all 5-AZA-dC-treated cells. The alterations seen in *N*-glycans from secreted glycoproteins in 5-AZA-dC-treated breast and ovarian cancer cells were similar to the *N*-glycans previously known to potentiate tumour cell survival.

**Conclusions:**

While the FDA has approved epi-therapeutics for some cancer treatments, their global effect is still not fully understood. This study gives insight into the effects that epigenetic alterations have on cancer cell glycosylation, and how this potentially impacts on the overall fate of those cells.

**Graphic abstract:**

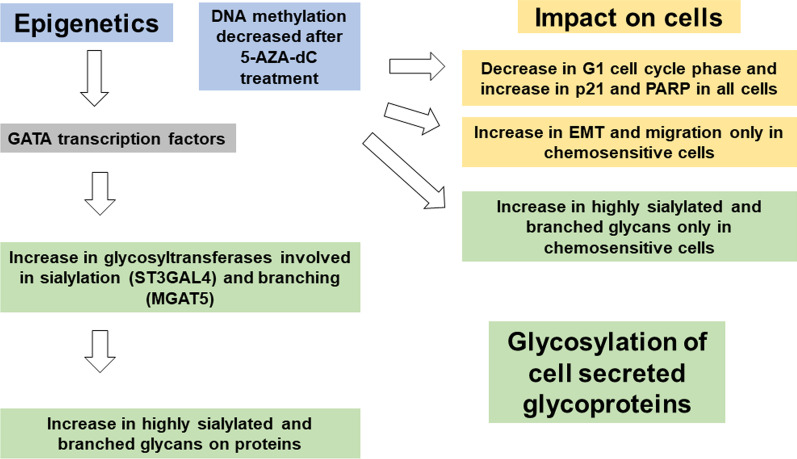

## Background

Breast cancer is the leading cause of cancer-related deaths in women; triple negative breast cancer (TNBC) subtype, accounting for 10–20% of the cases [1], is more aggressive and harder to treat [2]. Although ovarian cancer is far less common, more than 70% of women present at a late stage, giving them a less than 40% overall survival rate [2]. DNA methylation is altered in cancer, and DNA methyltransferases are attractive targets for epi-therapies such as Vidaza (*5-*AZAcytidine) and Decitabine (*5-*AZA-2′-deoxycytidine) (https://clinicaltrials.gov/ct2/home) [2], which were approved by the FDA for the treatment of myelodysplastic syndrome [3]. Global hypomethylation and site-specific hypermethylation have been observed on the DNA of both breast and ovarian tumours [4]. Promoter hypermethylation results in gene silencing as a consequence of transcription factors (TFs) being unable to bind to a promoter/enhancer region of a gene [5]. The hypermethylation of genes involved in cell cycle control or tumour suppression causes these genes to be silenced and promotes cancer initiation and progression.

Glycosylation is the most common posttranslational modification of proteins and is altered in cancer and other pathologies such as chronic inflammatory diseases exemplified by rheumatoid arthritis [6]. In cancer, the predominant glycan changes reflect increases in highly sialylated and branched glycans, which are associated with inflammation, metastasis and disease progression [6]. In a previous publication, Saldova et al*.* [7] demonstrated increases in highly sialylated and branched glycans on the *N-*glycans from secreted glycoproteins from the ovarian cancer cell line OVCAR3 post-5-AZA-dC treatment [7]. Similar results were published by Chachadi et al. [8]. Specifically, they reported enhanced glycosylation changes post-5-AZA-dC treatment that were associated with enhanced metastatic potential [8]. Both papers not only highlight the role that DNA methylation plays in glycosylation, but also the need for a more in*-*depth look at the effects of *5-*AZA-dC on glycosylation patterns in various tumour tissues, and on tumours of varying grade and stage [9].

The effects on cellular processes and end points when epigenetic alterations occur are well documented [10, 11]. However, with respect to altered glycosylation, processes such as cellular apoptosis and cellular senescence are less well understood. Research has been conducted, where cellular fate has been altered using various compounds to specifically target glycosylation. Gwak et al*.* [12] have shown that inhibiting *N*-linked protein glycosylation, using resveratrol, caused an accumulation of unfolded proteins in the endoplasmic reticulum (ER) of ovarian cancer cells and induced ER stress-mediated apoptosis [12]. In another study, NGI-1, an oligosaccharyltransferase (OST) inhibitor, was used to induce senescence in non-small cell lung carcinoma cells, by blocking the cell surface localisation of EGFR through altering glycosylation [13]. It is therefore warranted to consider that epigenetic tags and glycosylation should not be looked at as separate entities, but in combination, as they are concomitantly altered in cancer.

The current study follows up on the published work by Saldova et al. [7]. Our objective was to investigate whether changes in DNA methylation result in altered glycosylation, triggering differential cellular fate, in two chemo-resistant/chemo-sensitive pairs of ovarian and two triple negative breast cancer (TNBC) cell lines in-vitro*.* The impact of this treatment on apoptosis, senescence and epithelial to mesenchymal transition (EMT) is described. Survival analyses on online data repositories (kmplot.com) were undertaken to access the effect of glycan alterations in progression of ovarian and triple negative breast cancer (TNBC).

## Results

### 5-AZA-dC treatment alters global DNA methylation, the cell cycle and cisplatin sensitivity

**Fig. 1.**
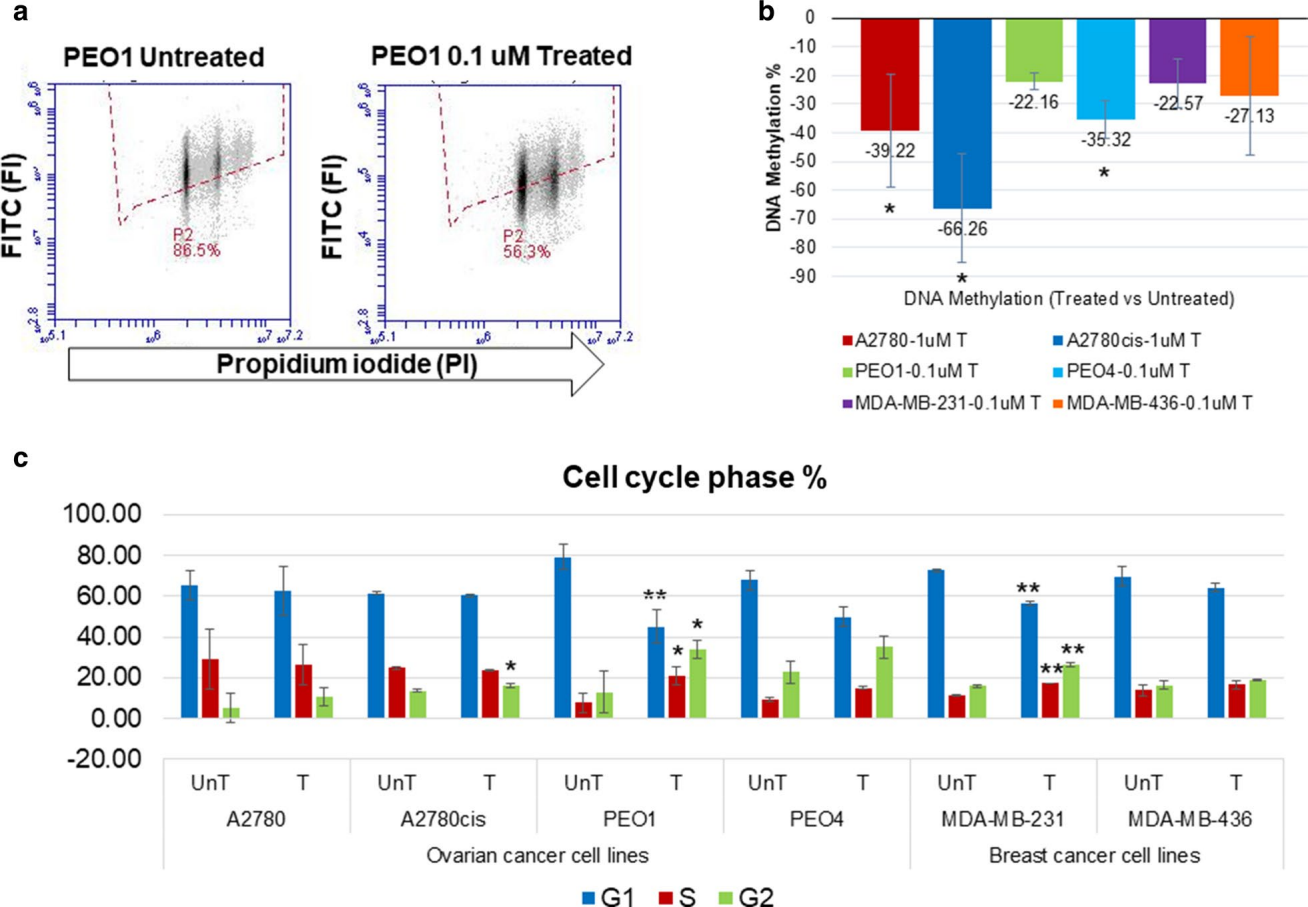
5-AZA-dC successfully demethylates global DNA. **a** Representative example of a flow cytometry dot plot for the PEO1 cell line, both untreated (UnT) and treated with 0.1 µM of 5-AZA-dC, measuring the fluorescent intensity (FI) of both PI and FITC. **b** Total percentage of DNA methylation post-5-AZA-dC treatment. **c** Histogram profiles, representing the percentage phases of the cell cycle (G1, S, and G2), before and after 5-AZA-dC treatment. All experiments are *n* = 3 biological replicates. Error bars represent ± SD. Significant changes are starred: *p value ≤ 0.05 or **p value ≤ 0.005 (*T *test)

Two pairs of chemo-sensitive/chemo-resistant ovarian cancer (A2780/A2780cis, PEO1/PEO4), and two TNBC (MDA-MB-231 and MDA-MB-436) cell lines were chosen for this study, to determine what effect 5-AZA-dC treatment has on the secreted glycome of chemo-sensitive/chemo-resistant cancer cell lines. The inclusion of two TNBC cell lines (MDA-MB-231 and MDA-MB-436) allowed for comparison with another tumour type. Briefly, A2780 and A2780cis cell lines were treated with 1 μM 5-AZA-dC and the remaining cell lines were treated with 0.1 μM 5-AZA-dC. Following 5-AZA-dC treatment, cells were harvested for flow cytometry dot plot analyses (Fig. 1a). Global DNA methylation was assessed using an anti-5′-methylcytidine antibody. Analyses showed a reduction in DNA methylation in all 6 cell lines, ranging from 22.16% in PEO1 cells, to 66.26% in A2780cis (Fig. 1b). The demethylation was higher in the chemo-resistant (A2780cis, PEO4), compared to the chemo-sensitive (A2780, PEO1) cells, significantly in PEO1 compared to PEO4 (*p* = 0.033) (Fig. 1b).

Post-5-AZA-dC treatment, the phases of the cell cycle were significantly altered in the A2780cis, PEO1 and MDA-MB-231 treated cells, compared to untreated controls. The G1 phase was decreased for all 6 cell lines, reaching statistical significance for PEO1 and MDA-MB-231 treated cells. The S phase was significantly increased in PEO1 and MDA-MB-231 treated cells, and G2 phase was significantly increased in A2780cis, PEO1 and MDA-MB-231 treated cells (*p* ≤ 0.05) (Fig. 1c).

Chemo-sensitive A2780 cells treated with 5-AZA-dC and cisplatin showed an increase in cell numbers (*p* = 0.044), whereas the chemo-resistant A2780cis cells showed a decrease in cell numbers (*p* = 0.004). This suggests that 5-AZA-dC treatment increased resistance to cisplatin in the A2780 cell line while enhancing sensitivity to cisplatin in the A2780cis cell line (Additional file 5: Figure S1).

### The effect of 5-AZA-dC treatment on glycosylation is cell line specific

**Fig. 2:**
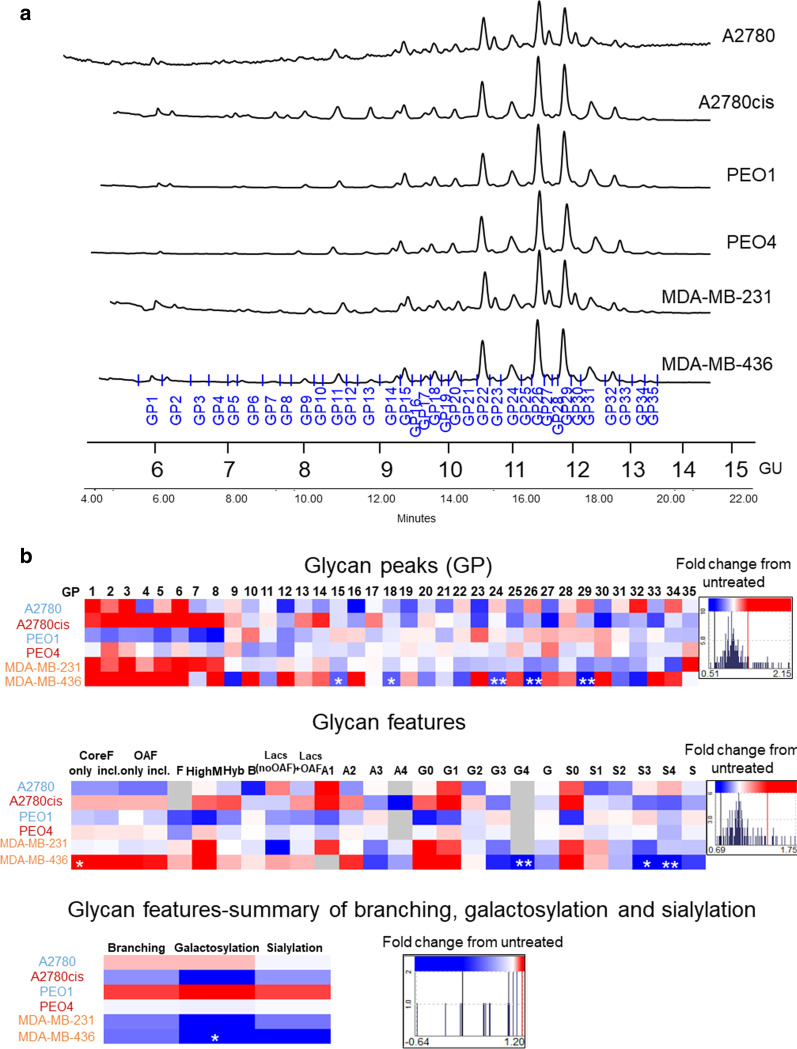
Glycosylation changes are cell line specific. **a** Representative UPLC chromatograms produced from secreted *N*-glycans of ovarian (A2780, A2780cis, PEO1 and PEO4) and triple negative breast cancer (TNBC) cell lines (MDA-MB-231 and MDA-MB-436) and their separation into 35 peaks. **b** Plotted peak areas from the secreted *N*-glycans of ovarian and breast cancer cell lines. The glycans in each peak (**GP1–GP35)** and features are listed in Additional file 2: Table S1. Significant changes (*p*  < 0.05) are starred: *P value ≤ 0.05 or **p value ≤ 0.005. (MANOVA). Heatmap histograms indicating fold changes in 5-AZA-dC treated compared to untreated cells, were created using Hierarchial Clustering Explorer HCE 3.5 software. Blue indicates decreases, and red indicates increases. The shade of colour corresponds to amounts of the decreases/increases

The HILIC-UPLC chromatograms from secreted glycans were separated into 35 peaks (Fig. 2a) and from the cell glycans into 39 peaks (Additional file 5: Figure S2A). The major glycans present in each peak from the secreted glycoproteins are listed and matched to the main peaks in our previously published paper [14] Table 1. The major glycans were pooled to the common features in each cell line. Branching, galactosylation and sialylation were calculated Additional file 2: Table S1. The preliminary assignments of the cell glycans and pooling into the common features using HILIC-UPLC and exoglycosidase digestions are shown in Additional file 3: Table S2. Cell and secreted glycan had differences in profiles. Specifically, while secreted glycans mostly contained highly branched and sialylated glycans, the cell glycans contained mostly oligomannosylated glycans.

**Table 1:** Main glycans on secreted glycoproteins in all cell lines

Peak	GU	Major glycans					
		A2780	A2780cis	PEO1	PEO4	MDA-MB-231	MDA-MB-436
1	6.37	FA1G1	FA2	FA1G1	FA2	M5	FA2
2	6.47	M5	M5	M5	A2G1/FA2[6]G1	A1G1S[6]1	M5
3	6.72	A2F1G1	A2F1G1	FA2[3]G1/A2F1G1	FA2[3]G1	A1G1S[6]1	A2F1G1
4	7.19	A2F1G1	A2F1G1	A2F1G1	A2F1G1	A2F1G1	A2F1G1
5	7.34	A2F1G1	A2F1G1	A2F1G1	A2F1G1	A2F1G1	A2F1G1
6	7.44	FA3G1	FA3G1	FA3G1	A2F1G1	M6	M6/FA3G1
7	7.58	M5A1G1/FA2G2	M5A1G1/FA2G2	M5A1G1	M5A1G1	M5A1G1/FA2G2	FA2G2
8	8.20	FA2BG2/FA3G2	FA2BG2/FA3G2	FA3G2	FA2BG2/FA3G2	FA2BG2/FA3G2	FA2BG2/FA3G2
9	8.47	M7/M4A1G1S1	M7/M4A1G1S1	M4A1G1S1	M7	M4A1G1S1	M7
10	8.62	A2G2S1	A2G2S1	A2G2S1	A2G2S1/A3G1S1	A2G2S1	A2G2S1/A3G1S1
11	8.97	A2G2S1	A2G2S1	A2G2S1	A3G3	A2G2S1	A2G2S1
12	9.13	M8	M8	M8	FA2F2G2	FA2F2G2	M8/FA2G2S1
13	9.45	M5A2BF1G2Lac1S(6)1	M5A2BF1G2Lac1S(6)1	M5A2BF1G2Lac1S(6)1	M5A2BF1G2Lac1S(6)1	M5A2BF1G2Lac1S(6)1	M5A2BF1G2Lac1S(6)1/FA2F2G2/FA2G1Lac1S1
14	9.86	A2G2S2	A2G2S2	A2G2S2	A3G2S1	A2G2S2	FA3′G3
15	9.99	A2G2S2	A2G2S2	A2G2S2	A2G2S2	A2G2S2	A2G2S2
16	10.16	FA2G2S2	FA2G2S2	FA2G2S2	FA2G2S2	FA2G2S2	FA2G2S2
17	10.32	A2G2S2	A2G2S2	A2G2S2	A2G2S2	A2G2S2	A2G2S2
18	10.47	A2G2S2	FA3G2S1	FA3G2S1	FA3G2S1/FA2F2G2S1	FA3G2S1	A4G4
19	10.64	FA2G2S2	FA2G2S2	FA2G2S2	FA2G2S2	FA2G2S2	FA2G2S2
20	10.81	A3G3S2	A3G3S2	A3G3S2	A3G3S2	A3G3S2	A3G3S2
21	10.98	A2G2S2/FA2G2S2/A2BG2S2	A2G2S2/FA2G2S2/A2BG2S2	A2G2S2/FA2G2S2/A2BG2S2	A2G2S2/A2BG2S2	A2BG2S2/A3F2G3	A3G3S1/FA2G2S2/A2BG2S2/A3F2G3
22	11.25	A3G3S2	A3G3S2	A3G3S2	A3G3S2	A3G3S2	A3G3S2
23	11.42	A3G3S2/FA3BG3S1	A3G3S2/FA3BG3S1	A3G3S2/FA3BG3S1	A3G3S2/A3F1G2S1	A3G3S2/FA3BG3S1	A3G3S2/A3F1G2S1
24	11.72	A3G3S3	A3G3S3	A3G3S3	A3G3S3	A3G3S3	A3G3S3
25	11.97	A3G3S2	A3G3S2	A3G3S2	A3G3S2	A3G3S2	A3G3S2
26	12.15	A3G3S3	A3G3S3	A3G3S3	A3G3S3	A3G3S3	A3G3S3
27	12.31	A3F1G3KDN3	A3F1G3KDN3	A3F1G3KDN3	A3F1G3KDN3	A3F1G3KDN3	A3F1G3KDN3
28	12.96	A3G3S3	A3G3S3	A3G3S3	A3G3S3	A3G3S3	A3G3S3
29	12.58	A3G3S3	A3G3S3	A3G3S3	A3G3S3	A3G3S3	A3G3S3
30	12.76	A3G3S3	A3G3S3	A3G3S3	A3G3S3	A3G3S3	A3G3S3
31	13.04	A3G3S3	A3G3S3	A3G3S3	A3G3S3	A3G3S3	A3G3S3
32	13.44	A3G3S4	A3G3S4	A3G3S4	A3G3S4	M5A2G2S2	A3G3S4
33	13.62	FA3BG3S4	FA3BG3S4/FA4F1G1S2	FA3BG3S4	FA3BG3S4/A3F2G3S3	FA3BG3S4/FA4F1G1S2	FA4F1G1S2
34	13.96	A2G3Lac1S2	A2G3Lac1S2	A2G3Lac1S2	A2G3Lac1S2	A2G3Lac1S2	A2G3Lac1S2
35	14.18	FA3′BG3S(3)4	FA3′BG3S(3)4	FA3′BG3S(3)4	FA3′BG3S(3)4	FA3′BG3S(3)4	FA3′BG3S(3)4

All *N*-glycans have two core GlcNAcs; **F**, at the start of the abbreviation indicates a core-fucose α1,6-linked to the inner GlcNAc; **Mx**, number (x) of mannose on core GlcNAcs; **D1,** indicates that the α1-2 mannose is on the Manα1-6Manα1-6 arm, **D2,** on the Manα1-3Manα1-6 arm, **D3,** on the Manα1-3 arm of M6 and on the Manα1-2Manα1-3 arm of M7 and M8; **Ax**, the number of antenna (GlcNAc) on trimannosyl core; **A2**, biantennary with both GlcNAcs as β1,2-linked; **A3**, triantennary with a GlcNAc linked β1,2 to both mannose and the third GlcNAc linked β1,4 to the α1,3 linked mannose; **A4**, GlcNAcs linked as A3 with additional GlcNAc β1,6 linked to α1,6 mannose; **B**, bisecting GlcNAc linked β1,4 to β1,3 mannose; **Gx**, number (x) of β1,4 linked galactose on antenna; **F(x)**, number (x) of fucose linked α1,3 to antenna GlcNAc; **Sx**, the number (x) of sialic acids linked to galactose. Where not specified, sialic acid is linked both α2,3 and α2,6

When comparing chemo-sensitive with chemo-resistant cell lines, some differences in secreted glycans were observed (Additional file 5: Figure S3), namely, an increase in GP13 (M5A2BF1G2Lac1S(6)1) in both chemo-resistant cell lines, A2780cis compared to A2780 (*p* ≤ 0.005) and PEO4 compared to PEO1 (*p* ≤ 0.05), and an increase in GP14 (A2G2S2 isoforms) in PEO4 compared to PEO1 (*p* ≤ 0.005). There were no significant differences in cell glycans when comparing A2780 and A2780cis cell lines.

In the secreted glycans, the variation in glycans between 5-AZA-dC treated and untreated samples appeared to be cell line specific (Fig. 2b). Specifically, when looking at the individual glycan peaks (GPs), significant changes in 5-AZA-dC treated samples (*, **) are observed in the MDA-MB-436 cell line, in which there is a significant decrease in **GP15, 18** (A2G2S2 isoforms) (*p* ≤ 0.05) and **GP24, 26, 29** (A3G3S3 isoforms) (*p* ≤ 0.005) (up to 0.51fold) (Table 1). Although not statistically significant, only the chemo-sensitive cell lines (A2780 and PEO1) show an increased trend (fields in red, Fig. 2b) in peaks containing triantennary trigalactosylated glycans, mostly trisialylated, but also disialylated (**GP22** = A3G3S2; **GP24, 26, 29** = A3G3S3). Chemo-resistant cells (A2780cis and PEO4) and TNBC cells (MDA-MB-231 and MDA-MB-436), show a decrease in these highly branched and sialylated glycans. Significant changes (*, **) were observed in the TNBC cell line MDA-MB-436, with a significant increase in the core fucosylated glycan (CoreF), a decrease in tetragalactosylated glycans (G4), and a decrease in tri and tetra sialylated glycans (S3 and S4). Although not statistically significant, only the chemo-sensitive cell lines (A2780 and PEO1) showed decreases (in the blue colour) in core fucosylated glycans and glycans with polylactosamine extentions with outer arm fucose as well as increases (in the red colour) in trisialylated glycans (changes varied from 0.69fold decrease up to 1.75fold increase).

When branching, galactosylation and sialylation were summarised, and MDA-MB-436 cells showed a significant (*) decrease in galactosylation (*p* ≤ 0.05). Although not significant, chemo-sensitive cell lines showed increases in branching, galactosylation and sialylation, whereas the chemo-resistant ovarian (A2780cis and PEO4) and TNBC cells showed the opposite trend, specifically, a decrease in branching, galactosylation and sialylation) (Fig. 2b).

In the cell glycans, peaks GP37 (FA4G4S(6,6,6)3), GP39 (FA4G4S(6,6,6,6)4) and tetrasialylated glycans (S4) were significantly decreased in treated A2780 cells (Additional file 5: Figure S2B).

### 5-AZA-dC treatment increases EMT and migration in chemo-sensitive ovarian cancer cell lines A2780 and PEO1

**Fig. 3:**
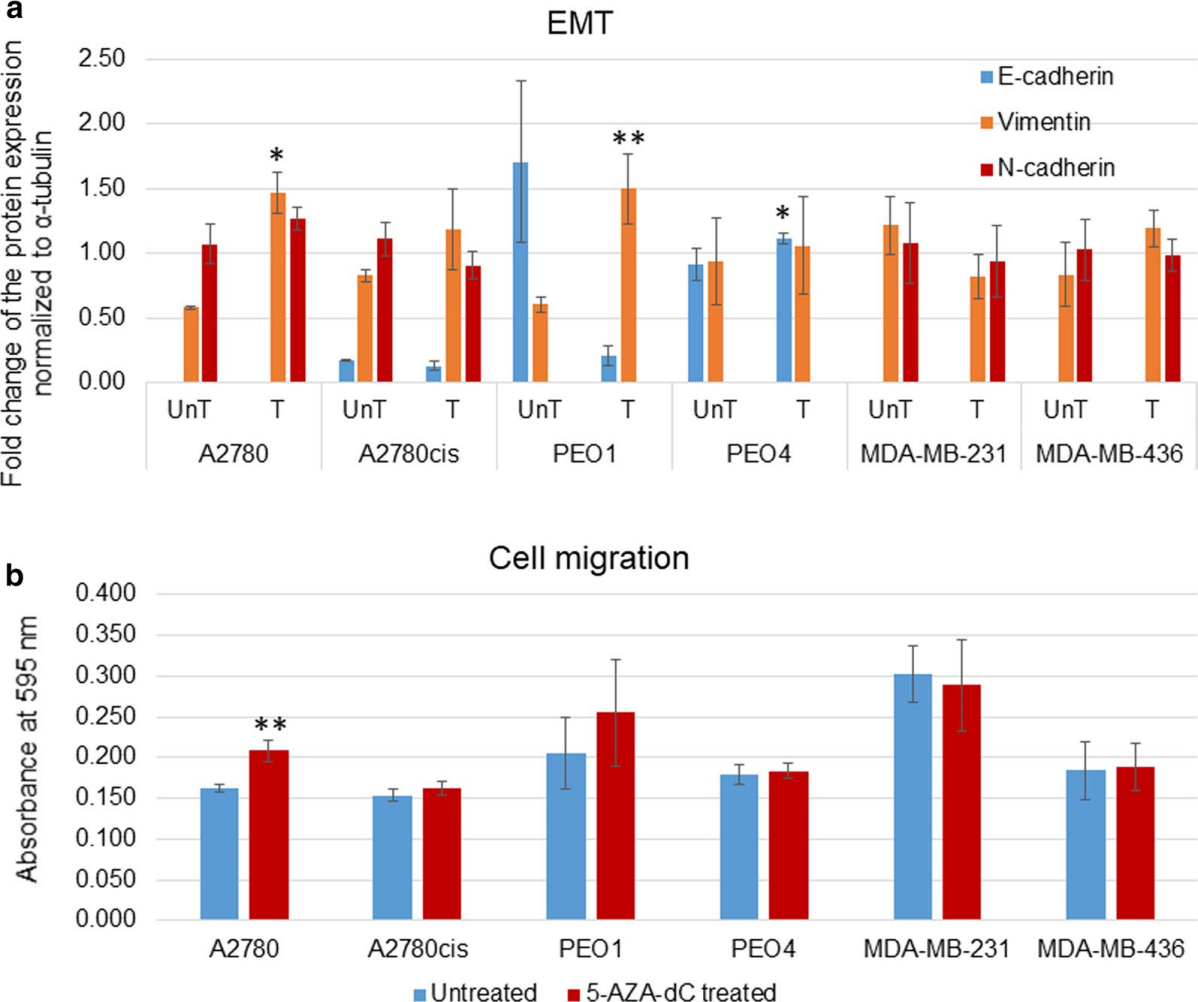
EMT and cell migration increases in chemo-sensitive cell lines. **a** Fold change of the protein expression of E-cadherin, vimentin and N-cadherin normalised to α-tubulin as determined by densitometry (Image J) (Western blots are in Additional file 5: Figure S4). Each condition represents three biological replicates. **b** Quantification of the absorbance readings from the Oris™ migration assay. A comparison of the absorbance from untreated vs 5-AZA-dC treated cells. Error bars were calculated from 3 independent experiments with 4 technical replicates per experiment. Significant changes are starred: *p value ≤ 0.05 or **p value ≤ 0.005 (*T* test)

Having identified changes in the secreted *N-*glycan structures post-5-AZA-dC treatment, we next investigated what impact 5-AZA-dC treatment had on tumourigenic phenotypes. Firstly, markers related to the EMT specifically, E-cadherin, which is downregulated in EMT and N-cadherin and Vimentin, upregulated in EMT were investigated (Fig. 3a) [15]. A2780, MDA-MB-231 and MDA-MB-436 cell lines expressed high levels of N-cadherin with no E-cadherin detectable before the treatment suggesting a marked mesenchymal phenotype (Fig. 3a). A2780 and PEO1 both showed a significant increase in vimentin (*p* ≤ 0.05, *p* < 0.005, respectively) and PEO1 had a trend decrease in E-cadherin (*p* = 0.051) post-5-AZA-dC treatment compared to untreated controls. This indicated an increase in EMT in these chemo-sensitive cell lines (Fig. 3a and Additional file 5: Figure S4). The chemo-resistant cell line PEO4 showed a significant increase in E-cadherin compared to un-treated controls (*p* ≤ 0.05), suggesting a decrease in EMT (Fig. 3a and Additional file 5: Figure S4). The other chemo-resistant cell lines (A2780cis and TNBC cells lines MDA-MB-231 and MDA-MB-436) demonstrated no significant changes with 5-AZA-dC treatment (Fig. 3A, Additional file 5: Figure S4).

Cell migration (Fig. 3b) of the 5-AZA-dC treated cell lines compared to untreated controls was then investigated. The Oris migration assay results showed a significant increase in migration in A2780 (*p* ≤ 0.005) post-5-AZA-dC treatment (Fig. 3b). None of the other cell lines showed any difference in migration post-5-AZA-dC treatment. However, although not significant, the PEO1 cell line had an increasing trend in migration (Fig. 3b). To see how the migration relates to the proliferation, faster growing cells (A2780, A2780cis and MB-MDA-231) were seeded at half the cell concentrations (2.5 × 10^4^ cells) compared to the slow growing cells (5 × 10^4^ cells) (PEO1, PEO4 and MB-MDA-436). Additionally, A2780cis cells, having a relatively high proportion of cells in S-phase (Fig. 1c), were treated with the solubilised dye after the migration results were measured, to see the amounts of the cells in each 5-AZA-dC treated and untreated wells. These results showed that there was no contribution of proliferation to the migration results (Additional file 5: Figure S5).

### Evaluation of senescence and apoptosis post-5-AZA-dC treatment

**Fig. 4.**
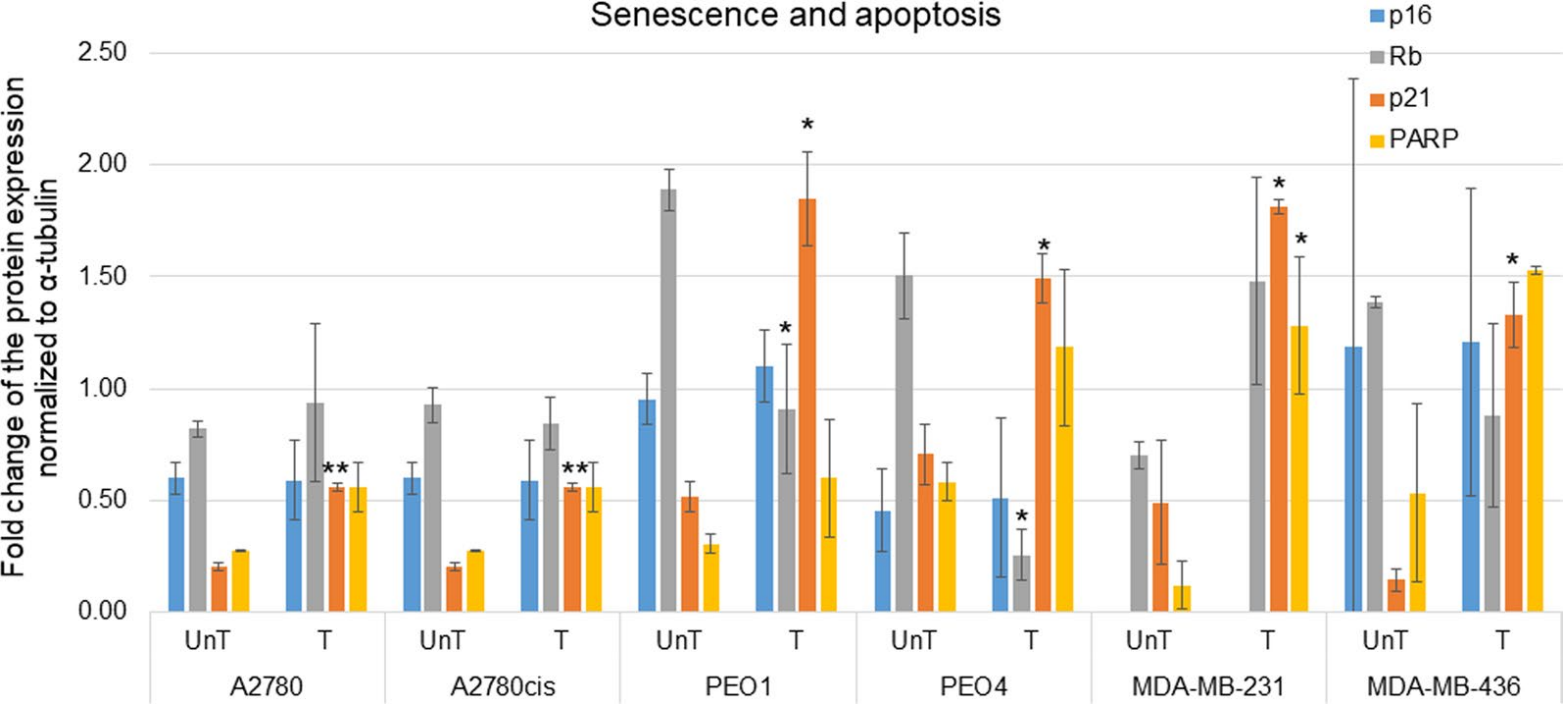
5-AZA-dC treatment results in increased expression of the cellular senescence marker p21 and increased PARP-cleavage. Protein expression of p16, p21, Rb and PARP normalised to α-tubulin as determined by densitometry (Image J) (Western blots are represented in Additional file 5: Figure S6). Each condition was undertaken on two biological replicates. Significant changes are starred: *p value  ≤ 0.05 or **p value  ≤ 0.005 (*T *test)

To determine what potential impact 5-AZA-dC had on inducing senescence or apoptosis, PARP was analysed as a marker of apoptosis and p16, retinoblastoma (Rb) and p21 for cellular senescence. 5-AZA-dC treatment induced a significant increase in p21 expression in all cell lines (*p* ≤ 0.05) compared to untreated controls. For Rb, there was a significant decrease in expression in PEO1 and PEO4 cells compared to untreated controls. In relation to apoptosis, there was a significant increase in PARP cleavage in the MDA-MB-231 (*p* ≤ 0.05) post-5-AZA-dC treatment. There were no significant differences found for p16 across all cell lines and treatments (Fig. 4 and Additional file 5: Figure S6).

### GATA2 and 3 transcription factors regulate expression of MGAT5 and ST3GAL4 glycosyltransferases

**Fig. 5:**
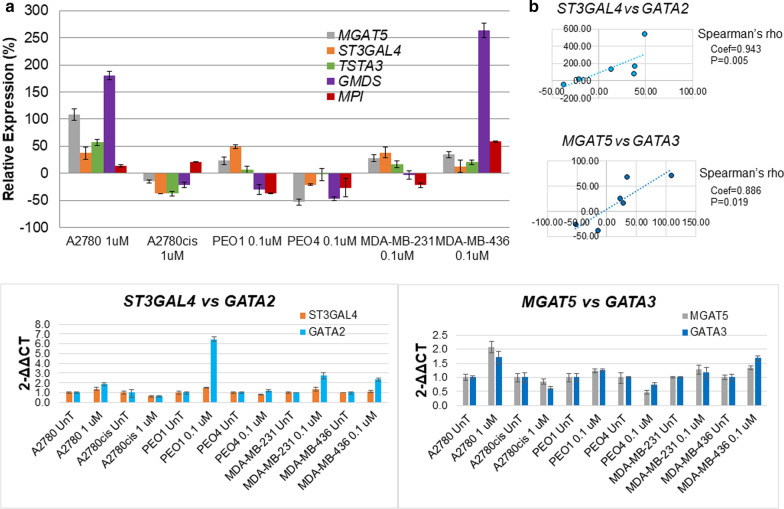
UPLC *N-*glycan changes are associated with the mRNA expression levels of glycosyltransferases. **a** Histogram representation of the relative expression of both the *MGAT5* and *ST3GAL4* glycosyltransferases and enzymes involved in the sugar nucleotide donor pathway (*MPI*, *TSTA3* and *GMDS*) in 5-AZA-dC treated compared to the untreated controls. **b** A comparison of the expression of the two transcription factors, *GATA2* and *3* with the glycosyltransferases, *ST3GAL4* and *MGAT5.* The relative expression level of each gene was calculated according to the ddCt method normalised to TBP. Each condition was undertaken as one biological replicate with 2–3 technical replicates. Spearman’s correlation was used

To further investigate the glycosylation changes seen in the UPLC profiles, the expression levels of the various glycosyltransferases and sugar donor enzymes were analysed (Fig. 5a). Preliminary results on one biological replicate (2–3 technical replicates) showed alterations in expression in all the enzymes analysed in all cell lines. Consistent with increases in highly branched and sialylated glycans, the expression of *MGAT5* and *ST4GAL3*, glycosyltransferases responsible for branching and sialylation, was also increased in chemo-sensitive cell lines A2780 and PEO1 post-5-AZA-dC treatment. Therefore, this suggested that these enzymes could be potentially regulated by promoter methylation. However, through preliminary analyses, only one of the two *MGAT5* RefSeq curated transcript variants has a CpG island (variant 1) and *ST3GAL4* is not subject to promotor DNA methylation (Supporting data1). Briefly, using data from the ENCODE project, we identified transcription factors (TFs) with binding sites in the proximal promoter regions of both *MGAT5* and *ST3GAL4*. While *GATA1-3* were not the only TFs with binding sites in the promoters of both *MGAT5* and *ST3GAL4*, the finding fact that all three were present in both promoters suggested to us that these TFs may play an important role *MGAT5* and *ST3GAL4* transcription and therefore warranted further investigation [14]. GATA1 showed very low expression in all cell lines (data not shown). However, increased GATA2 levels correlated with increased ST3GAL4 levels (coef = 0.943; *p* = 0.005), and increased GATA3 levels correlated with increased MGAT5 levels (coef = 0.886) *p* ≤ 0.05) (Fig. 5b).

**Fig. 6:**
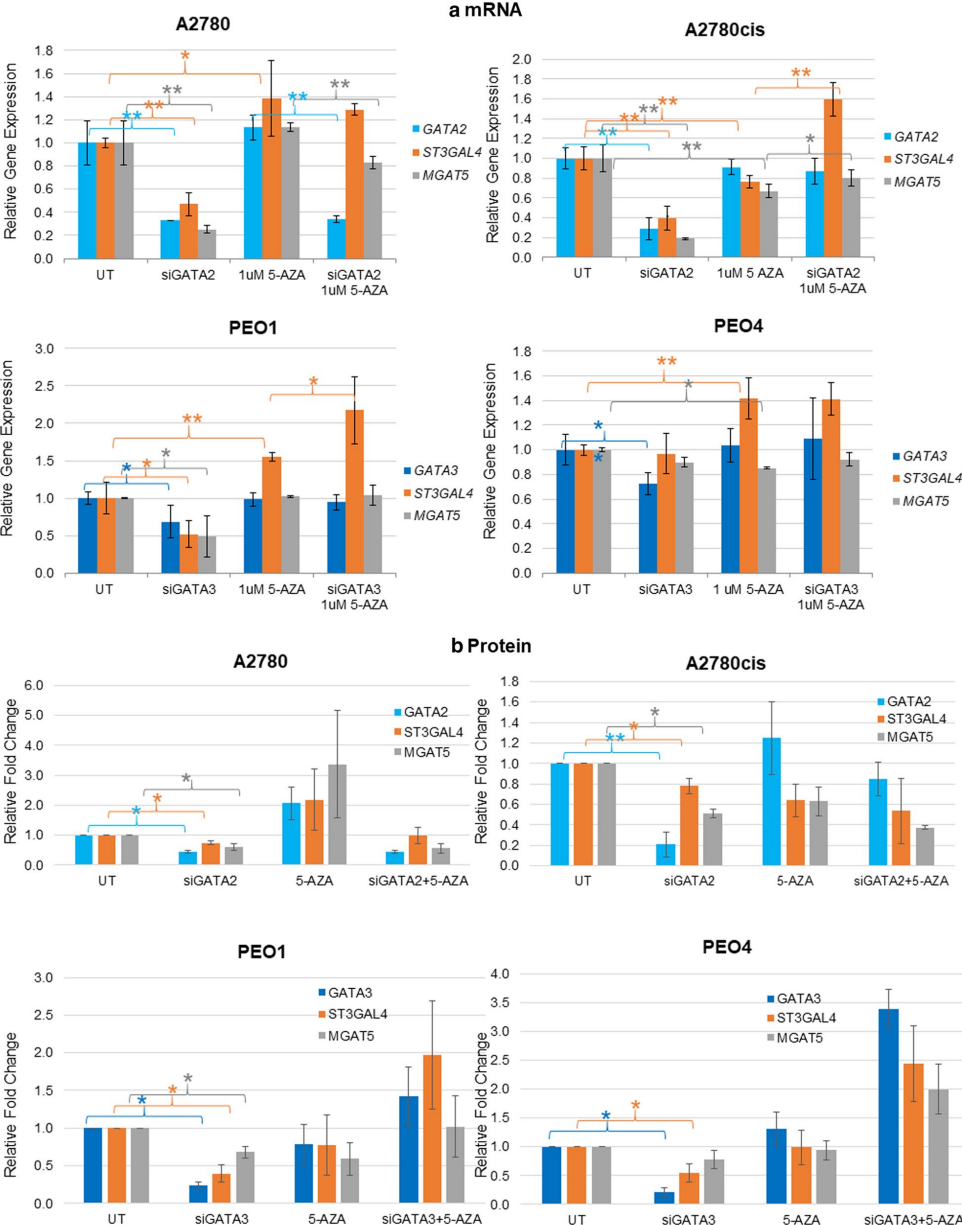
Expression of GATA2 and 3 affects the expression of MGAT5 and ST3GAL4. **a** Quantitative RT-PCR analysis of *MGAT5* and *ST3GAL4* and their transcription factors (*GATA2* and *GATA3*). The relative mRNA expression level of each gene was calculated according to the ddCt method, normalised to TBP. Each condition represents two biological replicates. **b** Densitometric (Image J) fold change of the protein expression of GATA2, GATA3, MGAT5 and ST3GAL4 following siRNA knockdown of GATA2 or 3 (Western blots are in Additional file 5: Figure S7). Each condition represents two or three biological replicates. *p value  ≤ 0.05 or **p value ≤ 0.005 (fold expression in particular conditions was compared as follows: untreated vs 5-AZA-dC treated and both conditions with vs without siGATA2 treatments). (Two-way ANOVA and Spearman’s correlation) All *p *values for this figure are in Additional file 4: Table S3 including the correlation between mRNA and protein expression)

Subsequent siRNA knockdown of the *GATA2* and *GATA3* was performed to determine the impact this might have on the expression levels of the glycosyltransferases MGAT5 and ST3GAL4 (Fig. 6, Additional file 5: Figure S7). For these experiments, the chemo-sensitive/chemo-resistant pairs A2780/A2780cis were chosen for their high expression levels of GATA2 and PEO1/PEO4 for their high expression levels of GATA3.

Following successful knockdown of GATA2 in the A2780/A2780cis pair, there was a significant decrease in the mRNA levels of *ST3GAL4* and *MGAT5* compared to non-transfected cells (Fig. 6a). For 5-AZA-dC-treated A2780 cells, successful GATA2 knockdown demonstrated a significant mRNA decrease in MGAT5 (*p* ≤ 0.005). 5-AZA-dC-treated A2780cis cells showed no successful GATA2 knockdown, and a significant increase in MGAT5 and ST3GAL4 was observed (*p* ≤ 0.05) (Fig. 6a). Following successful knockdown of GATA3 in the PEO1/PEO4 isogenic pair, there was a significant decrease in the mRNA levels of *MGAT5* in PEO1 compared to non-transfected cells (*p* ≤ 0.05) (Fig. 6a). For 5-AZA-dC treated PEO1/PEO4 cells, there was no decrease in GATA3, and ST3GAL4 was significantly increased in PEO1 (*p* ≤ 0.05) (Fig. 6a).

At the protein level, MGAT5 and ST3GAL4 were significantly decreased following siRNA knock down of GATA2 and 3, but only in untreated cells (Fig. 6b).

Finally, increased mRNA levels of GATA2 were significantly associated with increases in the mRNA levels of MGAT5 and ST3GAL4. In addition, increased MGAT5 protein levels correlated with increased protein levels of ST3GAL4 in all cell lines. Moreover, increased MGAT5 gene levels correlated with increased gene levels of ST3GAL4 in A2780 and A2780cis cell lines (*ρ* = 0.483–0.911) (*p* ≤ 0.05) (Additional file 4: Table S3).

### In silico survival analysis identifies increased ST3GAL4 mRNA expression as a marker of poor prognosis.

**Fig. 7:**
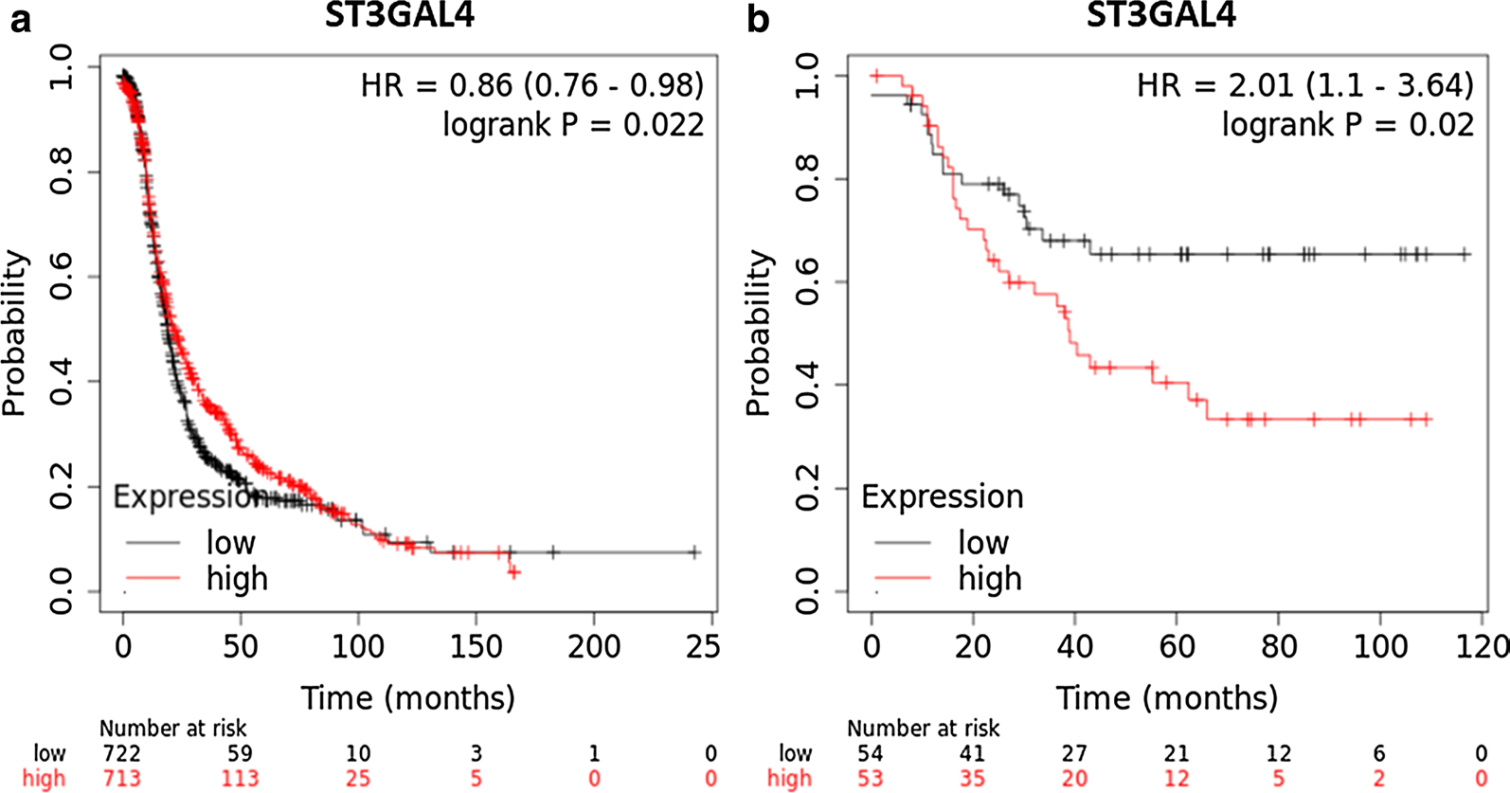
Survival analysis indicates the poor prognostic significance of increased ST3GAL4 expression. ST3GAL4 mRNA in **a** ovarian cancer patients in progression free survival separated by the median and in **b** lymph node positive triple negative breast cancer patients in recurrence free survival when separated by the median. *HR *hazard ratio

Increased ST3GAL4 mRNA levels were associated with worse progression free survival in patients with a diagnosis of ovarian cancer when separated by the median (logrank *p* = 0.022, hazard ratio = 0.86) (Fig. 7a) (kmplot.com). High mRNA levels of ST3GAL4 were also associated with shorter recurrence free survival in patients with a diagnosis of lymph node positive TNBC when separated by the median (logrank *p* = 0.022, hazard ratio = 2.01) (Fig. 7b). ST33GAL4 expression did not influence recurrence free survival in this cohort. Survival analysis of available datasets showed that MGAT5 expression did not significantly correlate with progression free survival and overall survival of patients with ovarian cancer or recurrence free survival of patients with TNBC [16].

## Discussion

The epigenetic link between glycosylation and cancer is a relatively new concept [2]. This study looked at the impact 5-AZA-dC had on the *N*-glycosylation of TNBC and ovarian cancer cell lines. DNA methylation was decreased in all cells following pharmacological demethylation, more in the chemo-resistant cells from the chemo-resistant/chemo-sensitive pair. 5-AZA-dC increased resistance to cisplatin in chemo-sensitive cells while enhancing sensitivity in chemo-resistant cell lines.

There were two glycans, namely hybrid pentamanosylated bisected outer arm fucosylated biantennary digalactosylated monosialylated glycan with polylactosamine extension and biantennary digalactosylated disialylated glycan, increased on secreted glycoproteins in chemo-resistant compared to chemo-sensitive cell lines.

Although not reaching significance, there was an observed increase in *highly branched, galactosylated and sialylated glycans on the secreted glycoproteins*, in the chemo-sensitive ovarian cancer cell lines A2780 and PEO1 consistent with the findings of Saldova et al*.* [7], where the ovarian cancer cell line OVCAR3 was studied [7]. The chemo-resistant ovarian cancer and TNBC breast cancer cell lines showed decreases in these types of glycans. Chakraborty et al. [17] and Saldova et al*.* [7] published conflicting results in relation to the changes seen in branched glycans post-5-AZA-dC treatment, Saldova et al. [7] accredited this to the variation in cell line models used [7, 17] and confirmed by this study. The impact these glycan alterations potentially have on tumourigenesis is significant, as increased altered glycosylation such as branching and sialylation is widely implicated in immune evasion, drug resistance, *metastasis and EMT* [18–20]. The markers analysed for EMT were epithelial (E)-cadherin, N-cadherin and Vimentin. E-cadherin is a protein necessary for apical-basal polarity [21], and N-cadherin and Vimentin are required for enabling cell motility [21]. This combination of EMT markers would then identify a cadherin switch typical of cells undergoing EMT. A2780, MDA-MB-231 and MDA-MB-436 cell lines expressed high levels of N-cadherin in the absence of E-cadherin before 5-AZA treatment suggesting a marked mesenchymal phenotype. Increases in EMT were shown only in the chemo-sensitive ovarian cancer cell lines A2780 and PEO1; the same cell lines with increased branching, galactosylation and sialylation on cell secreted glycans. These results correlate with the migratory capacity of these cells, as a significant increase in the migration of A2780 and a trend in an increase in migration in PEO1 cells was seen after 5-AZA-dC treatment. E-cadherin is one of the most important molecules in cell–cell adhesion in epithelial tissues and has been found to be shed from the cell surface into the secreted fraction in ovarian cancer cells [22]. MGAT5, responsible for increased branching of *N*-linked glycans, when overexpressed in a gastric cancer cell line, induced an error in the trafficking of the E-cadherin protein, resulting in its mis/re-location of E-cadherin from the cell membrane to the cytoplasm, resulting in reduction in cell to cell adhesion [2]. This is consistent with the finding that increased branching of the *N*-linked glycans on E-cadherin results in tumours with increased mobility and metastatic potential [23].

Differential markers which are well established with other cellular events such as *apoptosis and senescence* were examined. Firstly, PARP cleavage, a marker of apoptosis, showed an increase in all 6 cell lines post-5-AZA-dC treatment. 5-AZA-dC causes DNA damage inducing strand breaks and this recruits PARP, which functions in the DNA damage response and regulates DNA repair, which forms covalent adducts that induce apoptosis [24]. DNA methylation downregulates PARP expression [25], therefore, our results are consistent with the increased expression of PARP following 5-AZA-dC treatment in our cell lines. A marker of cellular senescence, p21, was significantly increased in all cell lines following 5-AZA-dC treatment. **P21** inhibits G1 phase [26], consistent with our cells decreasing numbers in the G1 phase, following 5-AZA-dC treatment. P21 is responsible for growth initiation, local tumour cell invasion and aggressiveness [27].

Another marker of senescence, p16, showed no significant variation in expression. **P16** is inactivated in tumours and is highly expressed in senescent cells [28], Rb is activated upon senescence by p21 or p16 [28], preventing premature senescence though DNMT1. DNA methylation inactivates p21 [29] and p16 [30]. Therefore, it is consistent with our significant increase in p21 in all cell lines. 5-AZA-dC is a 5-methylcytosine nucleoside analogue which can replace cytosine after conversion to its triphosphate form. Its main mode of action though is in the trapping of DNMTs and subsequently reducing their ability to methylate DNA [31]. This process takes numerous cell replications before the effect is pronounced. Therefore, p16, which is activated later for the stabilisation of the senescence phenotype, may need a longer time period before differential levels of p16 expression would be observed [32]. DNA methylation down regulates **Rb** [33]; however, we saw a significant decrease in Rb in PEO1 and PEO4 cell lines post-5-AZA-dC treatment. These cell lines have numerous mutations in BRCA 1 and 2 proteins [34], and the Rb gene is frequently inactivated by gene disruption in *BRCA* [35]. Interestingly, A2780 has BRCA1 and 2 mutations also [34].

*Expression of glycosyltransferases* responsible for altered glycosylation was investigated. We have looked at the glycosyltransferases which were previously found altered and responsible for changes on secreted *N*-glycans in ovarian cancer [7] and found that ***MGAT5*** and ***ST3GAL4*** correlated with increases in highly branched and sialylated structures on secreted glycans in the chemo-sensitive ovarian cancer cell lines. Our in silico survival analysis indicated the poor prognostic significance of increased ST3GAL4 expression in ovarian and lymph node positive TNBC patients, in agreement with the fact, that increase in ST3GAL4 and associated sialylation is associated with cancer progression and chemo-resistance [2, 23, 36]. Expression of other glycosyltransferases, such as MGAT3, which increased expression was found to be responsible for a decrease in core fucosylated tetra-antennary secreted *N*-glycans post-5-AZA-dC treatment in HepG2 cell lines [37], could be potentially also further investigated. Our *in-silico* analysis identified potential TFs with binding sites in the proximal promoter regions of both *MGAT5* and *ST3GAL4*, *GATA1-3*, which may regulate the expression of these glycosyltransferases. While GATA3 expression is required for normal development of the mammary gland, it has recently been reported to be overexpressed in many metastatic breast cancers [38]. Increased GATA2/3 expression is associated with a more aggressive phenotype and associated with metastatic disease [39, 40]. Both TFs are highly dependent on their methylation status for regulation [41, 42]. Knockdown of either *GATA2* or *GATA3,* resulted in a significant decrease in MGAT5, ST3GAL4, or both, on gene expression and protein levels, as confirmed by RT-qPCR and western blots. Interestingly, following 5-AZA-dC treatment, there was only successful knockdown of *GATA2* in the A2780 cell line with an associated decrease in *MGAT5*. In the A2780cis and PEO1 cell lines, the expression of the *ST3GAL4* and *MGAT5* was significantly increased after 5-AZA-dC treatment. Demethylation of GATA TFs may cause their significant increase following 5-AZA-dC, and therefore, this effect could mask the effect of the siRNA silencing. Decreases in the mRNA and protein levels of GATA2/GATA3 correlated with decreases in mRNA and protein levels of ST3GAL4/MGAT5. The GATA family of TFs contain six proteins, GATA1-6. It has been reported that aberrant DNA methylation on GATA2 affects GATA6 expression levels in gastric cancer progression [43]. 5-AZA-dC treatment may induce DNA methylation changes on GATA TFs and further contribute to disease progression. Considering only GATA1-3 was assessed, it may be possible that GATA 4–6 also have binding motifs on the glycosyltransferases and warrants further investigation.

*Study limitations and future perspectives* To further investigate cell-line specific patterns of glycan changes, future investigations would include whole genome methylation and transcriptome analysis post-5-AZA-dC treatment. To better characterise the roles of GATA2 and GATA3 in MGAT5 and ST3GAL4 expression, chromatin immunoprecipitation could confirm that both GATA2 and GATA3 bind to* MGAT5* and* ST3GAL4* genes thereby regulating their expression. The possibility that 5-AZA-dC treatment results in the hypomethylation these GATA TFs could be confirmed by bisulfite pyrosequencing. Additionally, binding of GATA may be methylation-sensitive. GATA1 TF binds to CGATA elements only if cytosine is unmethylated [44].

Based on our results, the 5-AZA-dC treatment, currently in the clinical trials, could be potentially beneficial for patients with chemo-resistant cancer but potentially harmful for chemo-sensitive cancer presentations; therefore, patients should be carefully selected for this treatment. Glycomics changes in glycosylation on serum proteins have been altered with cancer treatment and prognosis [6, 36, 45] and could be further investigated.

## Conclusion

Our results show that the alterations produced by 5-AZA-dC treatment are cell line specific. This study is the first to connect increases in branching and sialylation on *N*-glycome from secreted glycoproteins in chemo-sensitive cell lines to increase migration post-5-AZA-dC treatment. This was directly related to the altered transcription of the glycosyltransferases *ST3GAL4* and *MGAT5,* regulated in part by GATA2 and GATA3 TFs. Increased expression of ST3GAL4 was associated poor recurrence free survival in ovarian and lymph node positive TNBC patients. While more investigation is required, there appears to be a direct and novel link between the GATA TFs and these glycosyltransferases. 5-AZA-dC also triggers a therapeutic-induced senescence (TIS) and an EMT phenotype in the chemo-sensitive ovarian cancer cell lines A2780 and PEO1, with an associated increase in cellular migration. Based on our results, the 5-AZA-dC treatment could be potentially beneficial for patients with chemo-resistant cancer but harmful for chemo-sensitive patients. Therefore, our study is important for patient selection for this treatment.

## Methods

### Tissue culture and 5-AZA-2′-deoxycytidine treatment

The ovarian cancer cell lines A2780, PEO1, PEO4, and the TNBC cell lines MDA-MB-231, MDA-MB-436 were obtained and cultured as described in Greville et al. [14]. The cells were treated with 5-AZA-dC, specifically, A2780 and A2780cis cells were treated with 1 μM 5-AZA-dC, while PEO1, PEO4, MDA-MB-231 and MDA-MB-436 were treated with 0.1 μM 5-AZA-dC (Sigma)) every 24 h for 3 days (72 h). A dose response curve of 0.1, 1 and 2 μM 5-AZA-dC treatment was carried out for each of the cell lines with cell viability and DNA demethylation assessed. For A2780 and A2780cis, the optimum concentration was found to be 1 μM and for other cell lines 0.1 μM 5-AZA-dC was optimal. During the last 5-AZA-dC treatment, complete growth medium was replaced with serum free medium. Following treatment, the serum-free medium was collected for analysis of secreted glycoproteins. To investigate the potential impact of 5-AZA-2′-deoxycytidine treatment on cisplatin sensitivity, cells treated with cisplatin and combination of cisplatin and 5-AZA-dC were cultured at 37 °C with 5% CO_2_ for 3 days and cells were counted and compared.

Flow cytometry to assess DNA methylation status was performed as described in Greville et al. [14].

### Harvesting of secreted and cell glycoproteins

#### *N*-glycans from the secreted glycoproteins

Supernatants from the cultured cells were collected and concentrated using an Amicon Ultra-15 10 K ultrafiltration (Millipore) to a final volume of ~ 200 μL. Proteins were precipitated with a half volume of 50:50 TCA: acetone (w/v) on ice. The mixture was then incubated for 45 min on ice and centrifuged at 13,000 rpm for 5 min. The resultant pellet was washed with cold acetone and centrifuged again at 13,000 rpm for 5 min.

#### *N*-glycans from the cell glycoproteins

Cultured cells were harvested and resuspended in 250μL sample buffer (2% SDS, 62.5 mM TRIS pH 6.6) and left on ice for 20 min, pipetted again and left on ice further 20 min. The whole content was then drawn up and down 10times through a 21 gauge needle and centrifuged at 13,400 rpm at 4 °C for 20 min. The resultant supernatant was removed, and the pellets were dried.

These final pellets of secreted and cell glycoproteins were dried and resuspended in sample buffer for subsequent glycan analysis.

### Glycan analysis

*N-*glycan analyses including glycan release, labelling, HILIC-UPLC and exoglycosidase digestions were performed as described in Greville et al. [14]. Briefly, *N-*glycans were released from glycoproteins in samples by in situ digestion with Peptide *N-*glycosidaseF (PNGaseF; Prozyme) in-gel blocks, and fluorescently labelled with 2-aminobenzamide (2AB) by reductive amination. HILIC-UPLC was carried out on a BEH Glycan 1.7 μM 2.1 × 150 mm column (Waters) on an Acquity UPLC H-Class (Waters) coupled with an Acquity fluorescence detector using 30 min method and calibrated using a dextran ladder. The 2AB-labelled oligosaccharides were digested using arrays of the following exoglycosidase enzymes: *Arthrobacter ureafaciens* sialidase (ABS), *Streptococcus pneumoniae* sialidase (NAN1), bovine testes β-galactosidase (BTG), bovine kidney α-fucosidase (BKF), β-*N*-acetylglucosaminidase cloned from *Streptococcus pneumonia*, expressed in *E. coli* (GUH), and jack bean α-mannosidase (JBM), almond meal α-fucosidase (AMF).

### Feature analysis

Glycan peaks were pooled based on similar structural or compositional features of the peak glycan members. Features pertaining to a peak were determined based on the major glycan members of that peak (Additional file 2: Table S1 for secreted glycans and Additional file 3: Table S2 for cell glycans).

### Electrophoresis and western blot analysis

Proteins extracted from trypsinised cells using RIPA lysis buffer (BioRad) were separated by SDS-PAGE using a 4–15% precast TGX gels (BioRad) and transferred onto PVDF membranes using the Trans-Blot®Turbo™ system (Biorad). Blots were blocked and incubated with rabbit monoclonal antibodies targeting PARP, p21, p16, N-Cadherin, Vimentin, GATA2 and GATA3; rabbit polyclonal antibody targeting LC3, mouse monoclonal antibodies targeting E-Cadherin, retinoblastoma (Rb), ST3GAL4 and MGAT5 as described in Greville et al. [14]. The membranes were then incubated with a secondary goat anti-mouse or anti-rabbit antibody. The blots were developed using TMB for enzymatic colourimetric detection. To analyse protein loading, mouse monoclonal, α-Tubulin antibody (1:10,000; Santa Cruz, CA, USA) was used. Western blots were quantified using ImageJ™ software (FIJI).

### RT-qPCR (reverse transcription quantitative PCR)

Eight gene transcripts were analysed comprising of glycosyltransferases mannosyl-(α1,6-)-glycoprotein β1,6-N-acetyl-glucosaminyltransferase (*MGAT5*), β-galactoside alpha-2,3-sialyltransferase 4 (*ST3GAL4*), GDP-mannose-4,6-dehydratase (*GMDS*), mannose phosphate isomerase (*MPI*), tissue specific transplantation antigen (*TSTA3*) *GATA1*, *GATA2* and *GATA3* as described in Greville et al. [14]. TBP was selected as a reference gene [46–48]. At least two independent technical replicates were performed for each sample. Samples were analysed by triplicate in each experiment. Results were expressed as the mean ± SD values.

### Migration assay

The Oris™ Cell Migration Assay (Platypus Technologies) was used to assess migration in all untreated and treated cell lines as described in Greville et al. [14]. 2.5 × 10^4^ (for quicker growing cells A2780, A2780cis and MB-MDA-231) or 5 × 10^4^ cells/well/100 μL (for slower growing cells PEO1, PEO4 and MB-MDA-436) were added into stopper-loaded wells to normalise for the effect of proliferation. Proliferation controls were additionally treated with 1% SDS in water to solubilise the colour and incubated 1 hr at RT on a rocking plate. A reading of each well was taken at 595 nm.

### Transient GATA knockdown

Cells were transiently transfected with 100 nM of either siRNA targeting GATA2 (A2780, A2780cis), or GATA3 (PEO1, PEO4) (Dharmacon) transcripts as described in Greville et al. [14].

### Statistical analysis

All data are expressed as the means ± the standard deviation (SD). Statistical analyses were performed using SPSS statistical software for Windows (version 24.0; SPSS Inc.). For significances in glycan data, the HILIC-UPLC data were logit transformed and then used MANOVA and Tukey test. For Western blot densitometry, proliferation and migration assays, parametric *T *test was used. For RT-qPCR, a 2-way ANOVA was used with a post-hoc *T *test. *p *values were adjusted for multiple testing using the Benjamini–Hochberg method. The criterion for significance was set at *p value ≤ 0.05 or **p value ≤ 0.005.

### Survival analysis

The prognostic significance of ST3GAL4 and MGAT5 was evaluated using the Kaplan–Meier method (kmplotter.com). Progression free survival was chosen as the outcome of interest for ovarian cancer, while recurrence free survival was chosen as the outcome of patients with lymph node positive triple negative breast cancer. Expression levels were divided above and below the median into two cohorts; high vs low expression. Differences in survival was determined by the log rank test and expressed as a logrank *p* value. Univariate analysis was carried out using Cox proportional hazard models, and hazard ratios (HR) and 95% confidence intervals (CI) were calculated.

## Supplementary information

**Additional file 1:** Supporting data: Glycosylation gene promoter CpG island analysis

**Additional file 2:** Table S1.* N*-glycans and features from secreted glycoproteins from all cell lines.

**Additional file 3:** Table S2. Detailed assignments of* N*-glycans from cell glycoproteins of A2780 cell line using UPLC and exoglycosidase digestions.

**Additional file 4:** Table S3. P-values for siRNA knock down experiments (Fig. 6)

**Additional file 5:** Figure S1: 5-AZA-dC treatment increases resistance to cisplatin in chemo-sensitive and decreases in chemo-resistant cell lines. % viability of A2780 and A2780cis cells treated with 1 μM 5-AZA-dC and 1 μM 5-AZA-dC in combination with 1 μM cisplatin. * = P-value ≤ 0.05 or ** = P-value ≤ 0.005 (T-test). Figure S2: Glycosylation changes of cell glycans on A2780 and A2780cis chemo-sensitive/chemo-resistant pair. (A) Representative UPLC chromatograms produced from secreted *N*-glycans of ovarian chemo-sensitive- chemo-resistant pair (A2780, A2780cis) and their separation into 39 peaks. (B) Plotted peak areas from the cell *N*-glycans of these cell lines. The glycans in each peak (GP1-GP39) and features are listed in Table S2. Significant changes (*p* < 0.05) are starred: * = P-value ≤ 0.05 or ** = P-value ≤ 0.005. (MANOVA). Heatmap histograms indicating fold changes in 5-AZA-dC treated compared to untreated cells were created using Hierarchial Clustering Explorer HCE 3.5 software. Blue indicates decreases, and red indicates increases. The shade of colour corresponds to amounts of the decreases/increases. Figure S3: Secreted glycans differ in chemo-resistant comparing to chemo-sensitive cell lines. Plotted peak areas of GP13 and 14 from the secreted *N*-glycans of chemo-sensitive and chemo-resistant ovarian cancer cell lines. Significant changes (*p* < 0.05) are starred: * = P-value ≤ 0.05 or ** = P-value ≤ 0.005. (MANOVA). Figure S4. Representative Western blots of EMT markers in the 4 ovarian cancer cell lines and 2 TNBC cell lines post-5-AZA-dC treatment (T) compared to non-treated (UT) controls. Figure S5: Migration results are not attributable to proliferation. Migration (A) and proliferation (B) or the representative 1 uM 5-AZA-dC treated relative to the untreated A2780cis cells. Figure S6. Western blot analyses of the senescence markers p16, p21 and Rb and of cellular apoptosis (PARP cleavage) markers, post-5-AZA-dC treatment. Representative Western blots of the senescence associated proteins p21, p16, Rb and the apoptosis marker PARP of 4 ovarian cancer cell lines and 2 TNBC cell lines in 5-AZA-dC treated (T) compared to untreated (UT) controls. Figure S7. SiRNA knockdown of GATA2/3, impacts on the protein expression levels of MGAT5 and ST3GAL4. Western blot analyses of siRNA knockdown of GATA2/3 in 4 ovarian cancer cell lines (A2780, A2780cis, PEO1 and PEO4). GATA2 was knocked down in the A2780 and A2780cis cell lines and GATA3 in the PEO1 and PEO4 cell lines. Results are shown comparing (i) untreated controls (UT) with siRNA GATA knockdown UT (siG2/3) (left) with (ii) 5-AZA-dC treated (T) with siRNA GATA knockdown treated T (siG + T). All results are in triplicates (*n* = 3, biological replicates).

## Data Availability

All data generated or analysed during this study are included in this published article (and its supplementary information files).

## Abbreviations

5-AZA-dC: 5-AZA-2-deoxycytidine
5′MeC: Anti-5′methylCytidine
CI: Confidence intervals
DNMTi: DNA methylatransferase inhibitors
EMT: Epithelial to mesenchymal transition
ER: Endoplasmic reticulum
GMDS: GDP-mannose-4,6-dehydratase
HILIC: Hydrophilic interaction liquid chromatography
HR: Hazard ratio
GU: Glucose unit
MGAT5: Glycosyltransferases mannosyl-(α1,6-)-glycoprotein β1,6-*N*-acetyl-glucosaminyltransferase
MPI: Mannose phosphate isomerase
OST: Oligosaccharyltransferase
PI: Propidium iodide
PNGase F: Peptide *N-*glycosidase F
RT-qPCR: Reverse transcription quantitative PCR
ST3GAL4: β-Galactoside alpha-2,3-sialyltransferase 4
STR: Short tandem repeat
TF: Transcription factor
TNBC: Triple negative breast cancer
TSTA3: Tissue specific transplantation antigen
UPLC: Ultra performance liquid chromatography
