# Supplementary material for: 5-AZA-dC induces epigenetic changes associated with modified glycosylation of secreted glycoproteins and increased EMT and migration in chemo-sensitive cancer cells
Source: Clin Epigenetics. 2021 Feb 12;13:34. doi: 10.1186/s13148-021-01015-7 (PMC7881483; doi:10.1186/s13148-021-01015-7)
Supplement: Supplementary file 1 — Additional file 1: Supporting data: Glycosylation gene promoter CpG island analysis [file 13148_2021_1015_MOESM1_ESM.docx]

**Glycosylation gene promoter CpG island analysis**

**Methodology**

**Bisulfite sequencing & Methylation Sensitive High Resolution Melt (MS-HRM) primer design**

The UCSC Genome Browser (<http://genome.ucsc.edu/>) was employed to identify CpG island(s) within 10 kb up- or downstream of the transcriptional start site (TSS) of the five genes *MGAT5, ST3GAL4*, *GATA1*, *GATA2* and *GATA3*. This 10 kb bracket was justified because CpG islands have been located within the 5'-flanking DNA, promoter and exons of genes and with the recent discovery of “CpG island shores” (1-2). The CpG islands were characterised for transcription factor binding sites and promoter elements using Promoter Scan (accessed at: <http://thr.cit.nih.gov/molbio/proscan/>) (3).

PCR primer sets were designed to specifically amplify both modified, methylated DNA and modified, unmethylated DNA in putative promoter sequences and 5’ UTRs (Table 1). Where possible a CpG dinucleotide was included in the 5’ end of primer oligonucleotides, to reduce PCR amplification bias of unmethylated DNA (4).

**Sodium bisulfite modification**

Sodium bisulfite modification of genomic DNA results in conversion of unmethylated (but not methylated) cytosine residues to uracil. Genomic DNA from cell lines (500ng) was bisulfite modified using the EpiTect Bisulfite kit (Qiagen, UK), following the manufacturer’s guidelines. EpiTect methylated and unmethylated control DNAs were used as positive controls. *In vitro* dilutions of methylated: unmethylated DNA were generated to produce a series of controls: (100%, 75%, 50%, 10%, 1%, 0.1% and 0%) to establish melting profiles for individual PCR assays and to determine the limit of sensitivity of detection of methylated DNA.

**Methylation Sensitive – High Resolution Melt (MS-HRM)**

The 5’ CpG island and 5’ UTR of the *ST3GAL4* gene was screened for CpG methylation by MS-HRM, as previously described (5). Briefly, 20 µl PCR reactions consisted of 1X MeltDoctor^TM^ HRM Master Mix (Applied Biosystems, UK), 300 nM of each forward and reverse primer (Eurofins MWG Operon, Germany) and 10 ng of bisulfite modified DNA. PCR reactions were performed on a 7900 HT Fast Real-Time PCR instrument (Applied Biosystems, UK) under the following thermal cycling conditions: an initial hot-start at 95°C for 10 minutes followed by 40 cycles of 95°C for 15 seconds, 65°C for 30 seconds and 72°C for 30 seconds, concluded with a final extension at 72°C for 5 minutes. The melt curve/dissociation was performed immediately after amplification by denaturing PCR products at 95°C and allowing them to re-anneal at 60°C, according to the manufacturer’s guidelines (Applied Biosystems, UK). Dissociation curves were analysed using MS HRM v2.0.1 software (Applied Biosystems, UK).

**RESULTS**

**Analysis of *ST3GAL4* promoter methylation by MS-HRM**

The optimal PCR annealing temperature for *ST3GAL4* was empirically calculated based on its ability to produce different melting profiles of differentially methylated DNA (as constructed using dilutions of methylated into unmethylated DNA). MS-HRM analysis of a 345 bp sequence containing 19 CpG dinucleotides within the 3’ end of the *ST3GAL4* CpG island and 5’UTR revealed no methylation in an ovarian cancer cell line (Figure 1).

***Figure 1. ST3GAL3* methylation analysis by high resolution melt analysis**

Two 5’ regulatory sequences were analysed for NM_006278, A) genomic co-ordinates -723, -235, ecnopassing 68 CpG sites and B) genomic co-ordinates -220, +125, encompassing the transcriptional start site and 19 CpG sites. Dilutions of epiTect methylated into unmethylated control DNAs were used as controls.

| **Table 1. Primer sequences for bisulfite sequencing / Methylation sensitive High Resolution Melt Analysis** | | | | |
| --- | --- | --- | --- | --- |
| **Gene** | **Primer (5’-3’) Forward Reverse** | **Genomic co-ordinates Accession number; position (bp)** | **CpG sites** | **TM (°C)** |
| ***ST3GAL4*** | | | | |
| **primer set 1*** | CGGCGTTTTTGTTTGTTTTTG ACGTACAAAACCCTACCCCCTC | NM_006278; -723, -235 | 68 | 59.2 59.1 |
| **primer set 2*** | CGGGGTGGGGTTGTTATAGTTT CGACCTCCCACTAACAACTCCA | NM_006278; -220, +125 | 19 | 59.8 60 |
| ***GATA2*** | | | | |
| **CpGI 1 primer set 1*** | GTTCGTTTTTTGGAGGTAGAGATTTT CGAAATTAACTCATCTCCAAACAAATC | NM_032638; -4,919, -4,668 | 23 | 58.4 59.3 |
| **CpGI 1 primer set 1 (alternative)*** | GTTCGTTTTTTGGAGGTAGAGATTTT CCGACAAAAACTATATTTTTCCAAACT | NM_032638; -4,919, -4,550 | 38 | 58.4 58.2 |
| **CpGI 1 primer set 2** | CGGGTTTTTTTGTTTTAGTTGTAGAGG CCGTATCTCTACCCAAATATACCCTAA | NM_032638; -4,507, -4,210 | 21 | 60.4 58.1 |
| **CpGI 1 primer set 3** | GGAGCGGTGATATTTGTATGTTTATTT CGAATATATCAAACAAATATACCACAAAAA | NM_032638; -4,157, -3,754 | 20 | 58.8 58.1 |
| **CpGI 1 primer set 4** | GGGTTCGTATATTTGTATTTTTTTGTGG ACCCTTTCAAAAAACTCACAACAAA | NM_032638; -3,803, -3,520 | 20 | 60 59 |
| **CpGI 1 primer set 5** | TCGATAATTTGGAAGGTAGAGATAAGG ACCGACAAAACTTAACCTCCAAACT | NM_032638; -3,519, -3,180 | 30 | 58.3 59.4 |
| **CpGI 2 primer set 6*** | CGGTGGGGGAGAGAGAG CGAACCCCAAAAATAAAAACCAC | NM_032638; -22, +406 | 45 | 59.1 59.5 |
| **CpGI 2 primer set 7*** | GATCGGAAATTTTGGTTATAGGGA CGACCCCATTCTTATTAAAATCCC | NM_032638; +422, +873 | 40 | 58.4 59.8 |
| **CpGI 2 primer set 8*** | CGTTTAGTGGGATTTTAATAAGAATGG ACCTAAAACTCACCACCAAATATTCC | NM_032638; +841, +1,127 | 21 | 58.5 58.7 |
| **CpGI 2 primer set 9*** | CGTTGGTTTTTAGTTTAATGGAGTTTT GTCCTCCAACCCTCTTCCCT | NM_032638; +3,721, +4,211 | 42 | 58.3 58.1 |
| **CpGI 2 primer set 10*** | GGGGGTAGTTGGTGGTTAGTTATTG AACTACCGACTCCTACACAAACATAAAA | NM_032638; +4,944, +5,278 | 26 | 59.8 58.7 |
| ***GATA3*** |  |  |  |  |
| **primer set 1*** | AGGGAGAGAGTTGAAAAGGGTATTT TCGAAAAATCCTAACCCTCCC | NM_002051; -6,064, -5,776 | 9 | 58 58.1 |
| **primer set 2*** | TCGGTTGTAGGTAAAGTTTTGGTTTT CGAATCCCTCCCCCCTAAA | NM_002051; -4,390, -4,012 | 33 | 59.3 59.7 |
| **primer set 3*** | TCGTAGGAGGTTAGGATTAGTTTTGTG CCGAAACCCTAAACACAAAACAA | NM_002051; -3,034, -2,693 | 24 | 59.1 59 |
| **primer set 4*** | TTGTGAGTAGGAGAAGATGAGGGTAG CGTTTAAATCTTCTTTAAAATCCTCACC | NM_002051; -1,941, -1,679 | 6 | 58.1 59 |
| **primer set 5*** | GGTAGAGGAGAGAAGAGGGAATAATG GCGAATCTCCAAACCACTCTCTA | NM_002051; -1,463, -1,030 | 34 | 58 58.5 |
| **primer set 6*** | ATGAATGGGGTAGGTTGGTTGTAT CTCCTCCCTCCCCTCCACT | NM_002051; -391, -179 | 24 | 58.5 59 |
| **primer set 7*** | TAGTGGAGGGGAGGGAGGAG CGTCCTCTCAACCCCTAAAAAAC | NM_002051; -199, +314 | 27 | 58.5 59 |
| **primer set 8*** | TTTGTAGGTGATTAGAGGAGGGATTT CCGTCACCTCCATAACCTCTACTATAC | NM_002051; +576, +965 | 27 | 58.6 58.6 |
| **primer set 9*** | GCGAGTATAGTAGAGGTTATGGAGGTG CCTCAAATCCCTCCCTACCTCC | NM_002051; +934, +1272 | 32 | 58.8 60.9 |
| Note: Genomic co-ordinates are given in relation to the transcriptional start site. * Indicates primer sets that overlap with predicted promoter sequences or 5’UTR. | | | | |

**APPENDIX 1**

**ST3GAL4 (11q24.2(+),**[chr11:126,226,096-126,284,531](http://genome.ucsc.edu/cgi-bin/hgTracks?hgsid=167689897&db=hg19&position=chr11%3A126226096-126284531), 58 kb

RefSeq: NM_006278

**CpG island: 718 bp,** [chr11:126,225,356-126226073](http://genome.ucsc.edu/cgi-bin/hgTracks?hgsid=167689897&db=hg19&position=chr11%3A126225356-126226073), 87 CpGs

cgctggatggtaagcttcctggggacagggaatgggtctgatagtttcct

tggagcccctggcgctcccggcactgggctgttgatacgtggggctccgg

aaacactgtgctgacctgtgaaagggactggcctaaggggctgtggattg

tcctgtagggtgacttctgaactgttcttcagcctgggcagttcccaggt

ttatttggggggatgcctgggtccaaagaaccagggtcaggggcccaggg

gtttcttcctggagacaggatgcctggcgccttcaataacggaaaggaaa

ctgagaaactttctgaggccaagataaagagtatgcaagtgaggggtgca

gagagagcagtttaggggcagcaggccccaaccgaagcagaggaagggcc

cacgtctgggcccaggggactgactggctgccttgcgtgcatggggcacg

ggaagcataggctcccggaacaggaccctgccccagtttccccagtgagg

gaaggacaccgttccccgtgggatagagcactgcaccctttcttgattct

cattgttcccgggagcgagcgccttggctgcgctgggcatacccaccctg

gcgccattcacaggcagtgcctgccctggccctgtgctcaccccatcagg

Promoter 1

**cctcctcttccgcctctctggcacccacggcgcccctgcttgcctctgtc**

**cgggtctcggaggcgcgaacaccatcccaagcctgcgaccgcgcggcatc**

**acccccgcccttccccctcctccaccccgccctttctacccctcccggga**

**gctcctgggagtcgggccgccgtgtgcgcaagcgtgtccagccccttccc**

cgcccccaacccgggcccgggtccccgcctcccctgcaggcggactcgcc

cgctcccaggccggacccgcgcccgggacagggacccggccgagtcgagc

cgtcgcgccagcgctgcgccgccggtcggtgcgcctagcggatcggagct

CpG island

gcgcgcggaaccgtgctgccccgccccgctccacccgtgagggtgagtac

gcggcggcggtgcgcgggggcccgcggggcggggcggggcggggagccgc

ggggtcctcggccgcctgaccccagccggcgccgcgcctcccggaggggg

tcgggccctgcacgtgggcgcagcgcgggtcggggtggggctgccacagc

cctgcggagctgctttccggggtccctttccctggaccagattttcgcgg

gaagccggatcctctccgttcccttggagtgagcaagcgggacagttctg

cggaaagtttccgcccccaatcccccagccctgcgcccggactgaagcgg

cggcccccacctccagcatcctcgagcgatggtttctcccacaactaccc

cggtcaggtccagcacttgggagctgactgtgctggaggtgacaggcttt

5’ UTR

gcggggtccgcctgtgtgcaggagtcgcaaggtcgctgagcaggacccaa

aggtggagttgtcagtgggaggccggctgtgcccagggtcctagggccac

gttatgggggtcgtcaggcctaggccactgtgcctcagggctgcatgggt

ggtgagcagtgggagggatgcgatggggggagtgggctcagctgtctgct

gtgggtgtttcctcggggacctggctggcagggacctcccggccagccca

gaactgccaacatgtctgccagcactgcccctccattcccagtcctgggg

ggaggctctgtctgggcagcttgaggcctgacctactgtggctgcttcct

ctgctccccagcctgggtcctgagggcctggcttaagggaactgctgcca

gaaggctgggagtgtaggcagtgttcgggtgcccagctgtgccctgtctg

acctcatggccacccacaagctggcaccccccacaggcctcttggggaag

cctgtactttccaccgccggaggcgggtaccgcgtggaagccaggctcag

gcggactcattctttttgaactttgggaacaggcctggtacaggctgagt

tctcacaagagacacacacccgtgtcactgtctcctgagctgtgccggac

ccgtcctacagactccacctcctctaggtcctggccctggcatctgccag

ggggattttaaatgagttgatatatgctaagtgctttgaaaggcgcctgc

acggagggagagctgtataaatattcactattggtaatatttctcccatg

ctgttactctccaaagctgcccgataattctagagtgggttccggggacg

tgcccgctgtttggggcctagaaatgaactcgggaggttgttttgcttgc

tgagagctggtctgggaatgccccttgaccgttccctgccctgctcctgg

cccactcccagactgctcctgtgcctggccccattgcgctgggtgggtga

tcctaagctgccccatgggccctgggctctgtcaagacatggggagcttg

ccaggagttggggccagatcccccagactccaagatggtaaccgaggttg

tgagggtgcacatggaaggaagagttagaagagtgaattggaaattgcca

gcttggactttgggcagagtgggtgtctttagcttggggatgggttgttg

ttcagagacaacagggccgccttctctgtgagctggcggcccaggctgag

gtgcctctgggctgccttccctacgggaagcagtgactggccctctgctt

ctcaggaagagagtgccgtgcccccgccagcctcccaacctcgttagact

cctagaatccggttttctggatagaactaagaatctgctcctttctggac

ttttaatcctggaaagggctcccttccccagcaggagcagagagaccaaa

ccccctttctgtgctccatcttgagctgtcattctgtcctaactccccac

tggccaccatctgcccttgctttgggggctaccccaggtctgacttgggc

acgttctctgggtctctcctcagagaggctgctcctggggttgtcccaat

ggaattgggaattcttctcagaatggctcaaaggtttgcaccatctgttg

gagggaagggatccctctatcttggactaatacaaatgtttccgtgcctg

ccaagttccagaccctgggccgatagggaaaggagctggctggggggcct

gcaggggatggagagggagcttgggccccaagtggggtctgaagtccaag

cctacagattgccatcaaggcagccgctagagcctagcccagctcctggc

ccctcggagatggaccttagatgctggagagcggggcagtccagcagtgc

agggtgggacaccccgagcaaacagcccctgcttcctgccccctcagttc

agtcgcactgggccggctggacgtttccctgcaacaggcccctgctggtg

gtttagctcccgctccgtgtttccctcacgctgggatcctccttcctact

caagacccacgaccttgacctgaagcttttggttgccgggaggaaatggt

gagtgtaggcagatttgggtgactcttgttattaaccccctcttctttgt

tgcccccttcccagaggcttctgtatgagtgtcctgagtggccctggatg

gactactggctgtccctggcctcagaatcaccccaacccctgccacattt

cggggagggaggtgtgtgaatgatgtcctttattctacagactgaggaag

tggtcagtgtagcttcacgtgagagcagcagcgtgcgatgcccaggccct

agccaagtgctggtcagctttctgactggagcccctgagccctgcagggg

ctgaattcccccaaaggttgcttgggttgttgcatttcggggttagaggg

tgtgggtgtccagagctgtgggtttttagtgttggctgagccctttacct

gggacctacagcctggccacaaccccatctctgttcccctgtaccccaca

ctgccctgtgataggatgtgataagatggtaggattgcctatactgaagc

cctgggtgccactgctggcccagcagggaggaggttgctgctgctcgggc

tgaagtgaggtgtgggtctggctgggcctccagtttcccacctgggcctt

gattgtgaggaaggcctggcctggctgcagaagcccagaagcacctgagt

aggagagttcctttgtcccacctgcagctcattcaagcctgtgcatgggg

gttggggtcctcaggatcttgctttcctgtttaggggaggcagccccaaa

gagtgctgggaccagtttggagagtgctaaggaatgctggtctgcagcga

ccctacttgtgctctgcgtcctctgccaactgcagcatgggtgaacatct

gtacatctgtccccataatgaaaatggcctcagcaaataacaaaaatatt

accatttagcaatcaggcacttattaaaagcctggcccaataaacttaaa

aaaaaaaaaaagatgtgctagacactttacgtcccttcatgtgagcttca

cacaaccctgtaattaagcccatttataaatgaagaaatggaggattgaa

ggattaagttacttggccaaggtcatacagctcgtaagcagctgagctag

**gactcaaatccaggcttgcttccaaaaccttcacttttcagcccagttct**

**caactgccccctggagtttgctcattttgccccatgaagaaacagatccc**

**Promoter 2**

**agctcaaaggcgtgaaggggtttgctcaaggccacagtgtattagtgtca**

**aaggtggaacacaaaccctgacctctgagtgctctcccgaaccccacatc**

**ccggccgggccgtaccctgagcacacgcttgtccgctttctcagctgggc**

ggggcggggcaggacaaggttcttctccctccctccttccctccttccct

ccttccctccttccccgtgggccctcccagcctccccagcccccacctct

gctgctttcccttggcaattgcttccatctggcctgcctgcagctcctat

tcctttggaaatgtggctgccgcccacatggctgcaaggcaaccacggcc

acacgtggggaaggggaggggccctatggggtctaagcacagggaaccca

gccaaagtggctgctcctggcgtctggcatcctgattcctgccaggcaga

agcaccttggctgcctggccctccttgatgcccgtcctgggctggctcaa

ggccagagaggctcccaaagacccaattctccttccaacatggtgagctg

ttttgcccacagacaagctccgcaccggcagccctccctgtctcttagag

gttgctcaggttgggaggagggattcctggcaggagagaattaggggcca

gccggggtataccctgggttcccactgggttctgatcatttggagggaag

tcatgcaagggactgacgagggcagaggctgagggatccgcggagttctg

ggcaggcgaggaggcagctgacttgctcatgactgtgggtatgcgccacc

acactaggctaatttttctattttttgtagatacagggttttgccatgtt

gcccaggctggtttttgtttgtttgttttgttttgtttttgagacagagt

tttgctcttgtttcctaggctggagtgcagtggcgcgatctcggctaact

gcaacctccatcttctggtttcaagttattctcctgcctcagcctcctga

gtagctgagattacaggcacccgccaccacgcctggctaatttttgtact

tttagtagagacagggtttcatcatgttggccaggctggtctcgaactcc

tgaccttgtgatccgcccacctcagcctcccaaagtgctgggattacagg

cgtgagccaccgtgcccagccgagcgtgtctgtcttgttcactggtgtat

tttcttaatgcctggaatgatgtctagcaaacaataagcactcaatagat

tcttgtgtgatgagtgatggaaatgcagctcaggtatgctgatttgtctt

tgctgcatctttactttgcttcctcctcccagggcaaagggaaaaagttt

cccttttgccagttgagctgtttgccagagaaaggtctggccccatctgt

gtgctgccaccttgggggtctctgttcagacctcagagaggcaggcagga

acccaggtgaggatgggcaggagcttgtcccctgaggagtgcatgcccca

ttccacccaccacagggtcggctggccacatttctctacccatcacccct

ccctagtaaccttcaggtccctgcagacaggtcctgacctcagctccaat

ccctatgtcagtcctctccttatttgaggcccaagaaaggtacccctacg

gtggaaccctcacttgggtcctgagagggagtgacagctttggaagagcg

aggggttcacccacatttgcctcctcctgaacagctccacttttattcct

tgtacatattgcgtttccacttcaatttttttttgaaactgtgacagagg

gtttcttggctttaatttttttttttttttgaaaacccccattgagttcc

agttgtgacccccctgtggttctcattttctggtagcatgccgtgtgggg

tctcacctcctcgcctttgcttctgctgccccctcacatggcttgggcat

tgttggtaatgaccttgctagactgcaaatgtccttcagtatcaactgaa

gccccagtcctgggaagcctccgtggccacccagcaccggcacttcctcc

cccttcctccccctgcgttctccactccctgcaggtcctatgcctgtgac

agggagggaaccctgtgctggggtgaccccagcatggacctcagtggggc

tcaggctgaaccaaaggtaggtggttctgggggtagtgcctgaggtggtt

gtatggagctgtgagatttgtattcctagaagcaggttggaggctctcag

cccttccctccatgcatggccttggatacagcttgcagcctgccactgta

gcctccatttccttctccgagaaattaacacttctttttttttttttttg

agatggagtctggctctattgcccaggctggagtgcagtggtgcgatctc

ggctcactgccatctctgcctcctgggttcaagtgattctcctgcctcag

cctcccaagtagctgggatcacaggtgtgtgccactactcctggctaatt

tttgtacttttagtagaaatggggtttcaccgtgttggccaggctggtct

caaactcctgacctcaggtaatctgcccacctgagcctcccagagtgcca

ggattacaggagtgagccaccgtgtctggccaacatttcttccttttttt

tttttttttttttttaagacggagtctcactctgttgcccaggctggagt

gcagtggcacaatctcagctcactgcaacctctgcctcccgggttcaagc

gagattctcctacctcagcctcccgagtagctgggattataggtgcccac

catcacacccggctaatttttgtatttttagtagagatgggggtctcacc

atgttggccaagctggtctcgaactcctgacctcaaatgatccacctgcc

tcggcctcccaaagtgttgggattataggtgtgaactaccgtgctccgcc

tggccaacacttcttaatgggataatgtttcagataacatccagtgcccg

gactccagcactctataaatgctggctggtgtgcttttctgggattccag

ggtggggtccagagactgaagagggtctgctttctggcccaggggaagag

gggcaggtggctcactaggagctgtcctggcatcctttgtccccgtccac

ttaccagttatgtgacctcgggcaggtcttgcactctggcactcaattag

catctgtgttaattggggtaagagtatctgccacgtctcacagcctggtg

atggacaaacaaaacaaacattttttaggctgggagaggcctcagaggtt

ggctgtcatattgcaaacgagggagcagagactcagaaaagtaacttagt

tgtttaaggtcacagagcacctctctccccacaggcagggagccggtaat

tgggctgagcctcaggagtaacccttctctctctgcctctccagccccta

ccttatgcctggcccattcagctggtctgttctccaggcccctaggactg

ggtgggctaagatgagtggtctagaaatccctccccagctgggctggact

gaatgaggggctggccgaggagcgggtgttgatggagtatgggagattgt

cctcacaggcagtgtgggccgtttctctagacctcctgcccccatctcct

tcctgtccctgtttgagccgtgctcttcttcttcaatccctttctgtctg

ccttctctttccacccctagatttctcaccatcttcagtgatccctcctc

cttcctttctgcctcctctgcattctctccttgcccttgcttctctgatc

tgccttttttctccagcctcagcattcaggttgtcctcatagtggaactg

ggactctgggggtggctgggttgggttgcctggaggccgggaatgttcag

gagagccttcattgggagcccatgggagcaggagtcagctcctcctgttg

gcagctccagcattttcgaacaaaaatggctcttccaggagttgagtttg

gcgaagagacagtgcctgcccaaagtatgcaatgcttttatctttagctc

agctcctgagccctcatgcaactctttggacatcttcctgcaaaggacgg

tgggatcttgtctttctggggcctcagggtctccaaagatgggctgacta

agcttccctagcctgggacctgaaccctccaccctcctctctcttgagct

ctttctcccaggggaagctgctcgaatgaggggagttggccaacacagtt

ttctactctcagaccccattgaggtgagcacacaacagtgttctgtgtga

gggtctggatacactccccaccctcccaggcccggcaccgtgtcgccctg

ccccagcccagagcttgcctctgttggctttgtgaccctcgctttgtact

gtggctttgttctgccttagggaccagctggtcccctgctgtggttccac

acagtggagactgatccctgaccactcattcagctgcctggcggccctgg

ggcaagtgtttgtcccccatctcccaaaatggcggggtgcaggggctccc

tgcccggagtcccaaccctgccaaggcagagggaatggggaacagctgcg

ctggcccattccggctccatatggatttggtttgacaacaaagctccctc

tgtgtgttcccaagccccttacataaccaggggctgtgggcagaggagga

aaggaggaggaggtggagatggggaccaggagacaccaggatgggatggc

tggaaaggacacagggaccgggaggagagggcaggagacgagggccagag

aggaggagagggtgggagcaggagggggaagtgctggtcttgacctccca

gttctgcctcctgcttcctggggaggggagggtggagtccagccaccgac

ttctgcttgtggatgctttgtcttcttttctttggacctttcccaccagt

cctggataggctgaggaggggccgtgggacctgtctggggtgaggtgagg

tcaggtgttgggctggatgtgggaggctgttccttggccgcagcctgtaa

ctgaataaatgccattaaaaggggaatccatttcctccctcactcctggc

ttccattgccttcccccaggccccagccctggttccagaatccctctgac

cactcctcttgtgctcaggagggcagaataactgcagcctttccctgtgc

cctgccaggctggtcccgggccccctctcagcccactcatccagggatgc

agctctggccttggcccacccccatcagagggcctgtggccctgatcacc

gcactccccactctgtgaccagacgttctctcctgagcatcgctgaccct

gcagctgtccaaatagagcaagaagtgaatgttctctttggggcacagct

ggactggcagccctgcagggtcaggtgagctgccagtcctgggtgtgttc

tcgggggtgggggtgggaggatcacgtcctgggctgagatcccttcagct

cctcctgggcaggaggaaacagccatgcaggggcggccaggaggactggg

tggcgggtggttctgggctctcccagcttcggttggggctgggaccctgg

gggacatgctctcctgggtgttcccaggctctgtgtctggcctgtgcctc

agtgtgtgggttttctggcaccaggcttccttgggaagagcctctggttg

tgcaccagccctgagggtggggctcatcccaggctgcaggggactgaggg

ccgagctcttccaccttgaggacaaggatggttcctcagggctgggagga

ggaggaggcgctggccaggaccttagattacgcagggcatggtgttgggg

cctcacagatgatggagaagccagagttcaggcacttggcatgacccctg

gcatcgccttcagttgatttagtgtctctgccctgggatattccatggga

ccctgggctggcttctgtgtgcagaggtgggtgtgggccatagcccagac

ctgttgaaccagttgtaggctctggaatcctctgtccccaggtagtgacc

aggccctgacaggccggtatctgtccctcccatcaccctcccagttatgg

gtgcagatgaccctgaaattgctaaagcctcagggcttgagcccagaaag

tgtgagcaggctggtggggcagctggaggaaggccttgcaggttcctgga

tttgggtgccgggtgggctgagaggacagtccctgtcactgggactgggc

acaggtgcagagaggctgagacgggcccagaggacagtgagtgtgtgttg

tgtgggcgccgggccaggaagtgccctccatggggcagcaggctctgcag

taccctcggccaaatggccacaggctgccctgggacactctccaggcctg

ctggggacacacctgtggggctcataggcgtggctgggtctcctctcagg

tgccagaatctttctgggagctgagcctggctgggcagagggcgaggagc

ccaggattcccctttccgtgactttggaggaacgaggttcctgagggctg

gcacagccactccctgccccttagaggctccgggctgcctggtgatagct

cccagggacctgcaagctgacaaacaaagtctatacgagccagagctggg

gtgggaggaacccagtgggcctgggggaggtaaagcagcagcagctgctg

tagctgagcccggcccacggccagccctgaggaatggaggaagagagtgc

cctggctgggggagcccctcaagccctggaagctgtatgtgggcatgggg

tggtgggggcgtgcagagaggaggaggactgagagctgggtgtgtgtgtg

cgcatgcctgtgtctgtgtgatctggttcccgggtgtctgagtagggacc

aatgtgtttgtgctggagaagcctgtgtaggttactgacatggggagggg

tggggcttgctttcctcaccccgcttcccacgtcttcattgagtcctcat

ctgggggccctgttccactctccttcccgtgtgcagggccctcccctctg

gcgagtctgcctctcactcgctcagcccctttaaccctgctggctcctaa

tgagacttcaggtgtggaccctcgaaccctcagctgcacaggtctggaaa

catccttgttcatcaagcaccaggctgaccatggtgtccgtctcagtgcc

cagcagatgttctggtgaggggaggcgggagtgcaaggtctgccgagtga

gggttccttccgggaggaggcagggtgagcgcagaaaccactgctccaaa

actcttaaaagcagagccgagagcctgaattcagaggctgtactgtgggg

cagggctggaatttgtggcacctgcagggctgaacgttgcctttgctgtc

cctgcttggggattttggggtactgggtttggatagagtgtgtcatctgc

agagggagcactgttctggaagtggactctgctcaggtgtggggggcctg

cctgtagggggcaccgtggtggagatggagcaccgctcatgacaggaggc

tggtgtctggtcaggctgagggatccgtgtgccagcccttctgggaaggc

tgaatggggcggaactggtcagggcctggcaccaactccaggaggtgcta

cacccagacagacagatggaaagccacctccctagaggcctcctccccct

ccccttctgcatgccaccacaccccagcagcgtctcctgaggctgggcca

tgggtgaatctgagtcgagggcagggcagagtgggagtggcagtgcctct

gcctgtctccctggccgtggcaggtcctttgtcacgtatggaccaggaga

ggtgagtatgattgtggtagtggtgggggcagtggctgaggacccctctc

tttgctgggctggggcagagcttgaatggagaggggctgccttgtgcctg

ggaagggaggaccaggtgtcgctggtaccttccaggtcctgaatgcttgt

tctgtgcctagcccctgctgaggctgcggacgttacaaaccaggatgtgt

tttgaggctcaggacacctctgaggacttgctgctgttacccccatttaa

aagacgaggactctgaagatctgaggtgcatagagtgtagggattaaaag

tagttctggctgaattttaaatcctggttccaccatttttgagctctctg

atcttgggacccttaatgtatttgctctgtgcctcagtttccccatctgt

aaatgagaatgaaagagtaatcccacatccattggttgaaaacttcaata

agattatgcatagcaggccgggtgtggtggctcacaccagtaatcccagc

actttgggaggccaaggcaggtggatcacttgaggtcaggattttgagac

cagcctggtcaacatggtgaaaccccatctctactaaaagtacaacaatt

agctggacataatggcgcgcgcctgtgatcccagctacttgggaggctga

gcaggagaatcctttgaattcaggaggtggaggttgcagtgagccaagat

cacgccactgaactccagtctggaccacagagtgagactgtctcacaaaa

aaaaaaaaaaagattatgcacagcacctgcagcacagccccagggatgat

ttttaaaagtgaatccagggccaggtgtggtggctcatgcctgtaatccc

agcacttggggaggctgagacaggattacctgacatcaggagtcagtgac

cagcctggccaatatggtgaaaccccatctctactaaaaagaaatcccag

attctgccatgccacagtgtccaagtcagtcacctgctcgcagtcggccc

tcaggccctgtggctcctgtttctgctgacttgtccagcctcacagtctg

gggaggccttggggtccccagcctgcgctgggacaggggtattattccgt

cagcccttctgtggaacaagtagggaaggagtgatgtgacctccgtggct

ctcctgggacacaggttagtcctgtgggtgacctcactttagagagggct

ggccagctgtgccggaattaggaagtgggtacagctggacaaaaggtggc

tggagccagctctacagaagccacacctgctcatgagaagggtatccttg

tgagtacaggggaaatggcatggagaaagacaaacactggtgggtggctc

tgccagcttcccctggggtgcttctggcgatgcccaggcctttgcacgct

cagatctttgcaccccactgttttaacttggagaggagaggaggaagtta

gctgctctgaatggtgttgctgctaatctctacagcactttcctgtaaat

taggaacggtaacacacacacccctcccccaccccgttggagaagccctg

caccagagcatacagtttggcaagtagggggagggctggagcccagcctc

acttgccaccctggccaggccgtgattgcccagggtgcatactgtggctg

gtgctctgccacctgctgtttctgtcctcagggtaggacacaaggagggc

aagtagggctagtcagtgagaggcacaggtgatgcgtgttctagggcttg

agatcagctgcttccatttcagtaattgtctttggaggtacgttgtttgc

tcaagcaacctcccttcaagatagtgcctttttctttctctctctctctt

ttttttgggggggggaggcagagttttactcttgtcgcccaggctggagc

acagtggtgtgatctcggctcactgcaacctcggcctcctggattcaagc

gattctcatgcctcagcctcccgagtagctgggattacacgtgtgtgcca

ccacgccctgctaatttttgtatttttagtaaagatagggtttccccatg

ttggccaggctggtctcgatctcctgacctcaggtgatccgctcgcctgg

cctcccaaagtgctgggactataggcatgagccaccgtgctggctgatag

tgccattttttaaaatccctatttacccagtggaaaacctgagacccgga

aaggtcattcacactcccagaagtgatggttggtccagaattcaggtcca

agtgtgagacttcaacatttacattccttattttttcttcttcctttttt

ttattttaatcaaattattacttcaaaatgtatcacactcatcagtttga

aacagttaagcagtacaaatgggaatatgagagaaagtcactctcccacc

cgccctcttgagaggcaatagccttgttttgcatccttctgagtactcta

ttcacataagatgtatctgaatatgcatgtaattcttttgtgaaagtgca

tttggagatcactgttttacacctgccttttacatcttaatattatgctt

tggagatcagttgacatcagcatgcatcgatttgcatcctatttttcaca

gctgcatagtgtttcgttgtgtgtgcctaattttttaaaacaggtgcctg

tgcatagatttcatgtttccatcttatgctgttagaaaaagtgctgcagt

aaagatcctaacagacaagtctctttgcaaatataggaaacttttctgtg

gaataagcttctggcacggaagccccggggtaaagggtattttgataaat

attgtgaaatgtcttcccagaaggtggcctggttttcacttccagcatac

atctgtgagaggtcctgcttcccacactcccccagcactgtgtagctcag

gacccttgttttttgctgttactatttgtgaaaaatggtatctcaaccac

accggaagttttaaatgtagataaatgtcttatgtgccatttatatttct

ctacagtaaagtatatattcatagccttagcttatttttaaattatgttc

cttcattgtttggggagtggggggtagacttgcatgagtatgttataggc

taagggaattaactgataagttaatatatgtacatcttgcaaatgcactt

cttagttttttactcttttaagtatgagcttgctcacttgctctctgtat

ctcacttaaactttatgtgagtgtattaatctttcattatttagcttctg

aatttatatttcttcaatctccattcctttttttttttttccttgaaatg

gagactcgctctgtcacccaggctggagtgcagtggcacgatctcggctc

actgcaacctctgcatcccgggttcaagcaattctcctgcctcagcctcc

cgatttgctgggactacagacatgtgccaccacgctcggctaatttttgt

attttttagtagagaccattttggtcaggcaggtctcgagctcctgaact

caatcaatctacctgccttggcctcccaaagtgctgggattactggtgtg

aaccactgtgcctggccctcgttcttttttttatatgaaaatatttcaaa

tatgtaaaaattaatataacagacctccatgtgataatcagccagattta

tcaagtgtgaaccttatcccatattagatattttttcctttaagaaataa

aatattacaagtatagaactaaagatgtgctactatgatctattctattc

ttccccacattcctttttttttttttttttttgagatggagtctcactct

gttgcccaggctggagtgcagtggcgcgatctcggctcactgcaatctct

gcctcccagatccaagtgattttcctgcctcagcctcctgaatagctggg

attacagacgcacgccatcacgcctgtctaatttttgtatttttagtaga

gacggggtttcaccatgttggccaggctggtctcaaactcctgacctcat

gatccgcccaccgtggcctcccaaagtgctgggattataggcgtgagcca

ccgcgcctggcctattctttcatcatacacataagcacgcactatttaca

aatatacacaaacttagtgctgttttctgattcatcctttatctatttgc

aacactttaaaaagttaatgttatgttttcacattgatcccagttgacct

gtatggattaatttcttcagttgacatggctatataatattcagttgcgt

gaatgtagcacagcttatgtagccatccctctcctgacgatagttaagtt

gtttccattttttgctattgtaacactgctaataaacatgcttgaatggg

gtgtcccgtacaagggagggttactcggggctgtgcccggaaatgcaatc

gccagattgtaaggtatgcacgtgttcagcctgactacatattgtgaagt

gctcttctaagcctttgtatcaactggtactcccaccagcaatgtgtgag

aaactatatttggttttaatctgtattccctggttactagtgagcctgag

catcttttctgtctattaggtgtttgaatttaatctgtgaattgcctttt

tgtgttcttttttattcctttgattgttttggtggtggttatgataaaat

gcacaagcacaaggttctgttgacctgttgaccgtcttaaccctttttat

gtgtatagttcagtggcattaagtacatttagaatgtgctaccatcccgc

catccagctccagaaatcttttcgtctcgtcaaactgaaacatcctcctg

ctagtcaatggctcatctccttttcccctcagtccttggcagccgccatt

ctgctttctgtgtctatgagtttgactgcttttgagtacctcctataagt

ggaatgatacagtatttgtccttttgtaactggcttatttcactttgcat

agtgtcttcaaggttgatccatgttgcagtgtatgtcagaatttcttttt

ttcttttcttttcttttctttttttttttttctgagacagagtttcgctc

ttgctgcccaggctggagtgtaatggcgcgatctcagctcaccacaacct

ctgcctcctgggttcaagcgattctcctgcctcagcttcccgagtagctg

ggattacacgcatgcaccaccacacccggctaattttgtattttcagtag

atacagggtgtctccatgttggtcaggctggtctcgaactcccaacctca

ggtgatctgcgtgcctcgacctcccaatgtgctgggattaaaggtgtgag

ccattcacccggccgtctttggattttatacagcaaaactccataatatt

tcctttacatgaatttcctttctttttcaggctgaataatattccattgt

acgcatagatcacattttgttcattcatccattgatggatacttgggttg

cttcccgattagctattgtgaataatgctgctatgaacatgggtgtacaa

atatcgctttgatattctggtttcagttctttggggtatataccccgaag

cacagttgtgggatcctatcttgtctcttgttttctctttgcttccatgt

tttttctcattgtttcgtgggaatactgatattatatattcaagtatttg

ttgattacatgcactgtacctgacttgtttaaagcctgatgtcttgagta

ctgtgcacccctgtgcctggctgagtagtggaggaactttgaaagggtct

gttctggagagggggtgctgctctggagaggcggtgctgctttggagatg

gattaatcaggaacttgggtgctgattctaggaggtactgcatctagaaa

gatgggaggtggggagtgcaacttccccaattttgcctttaacttcagat

ccttggggtgggtgcattgcttctcctgggatggaggggtgccggggagc

ccctgaccaggacctggagcaggtccacacaggcactggatttgtccttt

actttgggcccaggctgcactcttgattccacctcctcacactggcttca

gctttctcaccctcagaatggaattcaaagcctctgcccctcccaggcag

acaccaaggtatgattttcaagtggttggtgtcttgtgactggcagctga

taaacgggtgatacagtgtttctatacaggagtctgcagtgccaggctgc

aggggcggacgggagggggtcgtggtgggtagcctgtggagcctgagggt

gggaacagagagacttcttttgctgtaggtggaagcctgtctggctcctt

tccctcaaaggtcagacagcttgagagatgtttccatcctcccatgcatg

accctgaggcagttagctggtcagttactcactgtgctctctgccaactc

taatgagccaggaagcctccccgtgcccaggcgtgctgtgatcacagtgt

tcaatgatggggtgaggctgcagcgtccagccagggactgtgccgcaggg

gccagaagatatctgacctactgtcactgcctcttccactctgttccacc

tgcactggagcgatgctatccgatgaacagatccatttattaattcaaca

cgtttgtattgagagcctgctgggtgccgggcactattgtcaacatgggt

aatacagcagcaagcaaaacaagagaaacatctttgcaagcaggggaggg

ggcctgcagtgttgggtgtgtggagttgttgctgggaagtagagaagctg

agtaagagctggacaaagaggccaggcacagtggctcacccctgtaatcc

cagcactttgggaggccgagtttggcagatcacttgaggtcaggagttcg

agaccagcctggacaacatggggaaaccccgttcctactaaaaatacaaa

aattagctgggtatggtggtgcatgcctgaaatcccagctacttgggagg

ctgaggcacgagaattgcttgaacccgggaggtggaggttacagtgagcc

aagatcacacaccattgtaccccagcctgaatgacaaagcaagactttgt

ctccaaaaaaaaaaaaaaaaaaaaaaaatagagaaaaggaaagctggatg

aagagagatagccaagcccatgtctacggaaaagcattcttgactgagtg

aacgaaagtacaaagcctcagaggagggagcatgcctaggtagcccccta

gacctggtgaggctggtgcgtggggcagcctccatggagaggagttgggc

aaggagggaggaggtgctggccagtagagcacgtgtgtctctgtgcgtca

gagcaaggacccgggctctccccgtaagtgagaagagtccatggaggttt

tggggctgaggaggagttttacagatgaccggtctgttcttggcacttgc

aattagcggaccgtctgtatccgagtgttgatcgggtggattctaaccct

gtgggagaactccccagcccgtagcctgcgctcactgtacctggcagttg

tattaatgacgtcaaatgcacagtccagcttgagtcccctctagaacgtg

ggcagcggacactaagccgagaggaatcgctgtgggttggcagacactgt

ggagactgactcctggacaggctgtggaatgtgtggaagcaggtacagcc

caggcccgcgctgaagttcggcagggaagccagaggagtccgcagcccgg

gagacctggcctacagcggagagagtcagtccatgccagtggtgggatgt

ggctgcaaaaaatggtcggattcatggaagtctggtgtccatctggcagg

cgttagtggtcccacgaatacagctgtgtgccttgaacacatactgcaga

gcttccagagagcaccagggctgtgggggctagagactgtgtctcgtgcg

gaagcgtggctgaggaggggcttgacaggctcttcgtcactgtgcggagc

cttcgaaggacttggatgtgcttgagaccccttcccctaattgctgtgta

gggcaggggtggacatcccggggatcgtggagtctagggagccagtctgc

acgggatggttttagaagccgtaggatgtgggggttctggctgaagtagc

cagaattccagtgccagctccgctcctcacgagctgtacaacctgggccg

gttactgacccctttcccaccttagttccctggctctacaatggaggtaa

ttatcgtgttgaactcacagggtcgagatcatatttaatacttgcacagc

ccttagaagcatctagcacagtggtcctcagcatttggaaatgtctggaa

acattctgtcacacctggggagtgggatttccttctggtgtctagtgggt

agaggccagagatgctgctaagcgtcccacaatacacaggacagaccccg

caacaatgaactgtctggcccccaaagttagtggggcagagattgcagga

tcctgattccttttcttccttttttttttttttttttttgcccctggcta

tgagtaagagatcctggtttcgcacaaagtgaacgtgcagtacattgttt

cagttgttaattcgccatgcaatacatcgttagtcacgtattattgtttg

tcaaatgtgtataaaacatctgccacattcttggcagtttaatagaatct

agagatataaagaagttcctgaccacagagttcatagcctcacaggcaag

ataggaaaacacagaattatataatacgaaattacattatatcatagagc

atattctatagtaagttactccgattggcttttggtattaaaatgtcttc

agtttcagaggtcccaggatagtgggcaatatttgaaagccagtaaatat

aaaataaaatatattacctgtgtgaaagagaatttttcaggcacaataat

attttaaagagtttatttgtgtaagaagtgaattgggaaacagaaaaccg

aaagaggtttagtggcccaatgacagtttcagaggcaggtgtttggagag

tgaatgtaaaagcaaaatgaagaatttatttgatttaattggttgcaatt

acaaaattgctttgtttggtttaccttgttggaagtttcctagtcacata

accatatgttagttggctacttatgattggctgaagttttatttgtgtca

aacagaagcatttaccataaataaccccagttaagatttgattatgctgt

cttttaagcaaggttaaggctgttgttaaggcctcattggttttgtttgg

taaggaatttttcaggcctggtctccaaattttgctttaacaattccttg

cttctggttattctctcagcaagctgatagtgtgactagatgacacagta

ttactctctgttaccaccaccaatgtagtcactgggacaaagagtcaggt

agatgttgttgtcatcatcagtaggtaggttggggatgagaattccatgg

ccacagttagcagttacatagtcagtgttgctttcctcaagttgaatgat

cctcatggtaactgtttgatatttgagcagctgttggaaatcatttagaa

cccttgagagaatacaaggcaccaggggggtaaacataataattataagg

agaataatagccaaggattgaaaaattcctctgagcaaagattcccagga

gccaagacttaaccagctgagtgaatcaatggagaaggcagtgcccatct

aatgaaaaacccatgtgtgttctttaaggtattttaagttttcggtaata

cctgatttgttaacataaaaatgacactttttttttttgagatcgagtct

tactctgtcgcccaggctggagtgtagtggcgcaatctcagctcactgta

aactccacctcccaggttcaagtgattctcctgcctcaacctcctgagta

gctgggactacaggtgtgtaccaccacacccagctaatttttgtgttttt

agtagagatgggatttcaccatgttggccatgctggtcttgaactcctga

cctcaggtgatccacctgcctcagcctcccaaagtgctgggattacaggt

gtgagccaccgtgcctggccaacaccttctttttttttttttttttttga

gatacagtctcactctgtcttgcccaggctggagtgtgcagtggtgtgat

cttggctcactgcagcctctacctcctgggttcaagtgattctcctgtct

cagcctcctgagtagctgggaatacaggcgtgtgccaccacacccggcta

attttgtatttttagtagagacagggtttcacccaggctggtctcagact

cttgacctcaggtgatccacctctctcagcctcccaaagtgctgggatta

taggcgtgagccatgcgctcggccagcaacacctttctaccaattaggga

acaaggtctccctttttgagcagttaaagtagcaagaagtcttctattgt

acagtactactgaggccaattcattcatcccagattgtactgcttaagat

aattcgaagtgtcaattaaactttttcagaagttggtaaaatattaattg

atttttttcaattcactcaactgagagaaacgggctcttacagaagtatg

aaatccagaaccccttcgtaagactgtgtctgggatattatgattctctt

ttgtgaggggtcactttatgtacaaatgactctaaggatttgtatggcaa

ttgaagagtcatatctagtcattttaggtagaagatagcccacaccacag

atccagactggctaggtagaacacctttacatactttgttgctgcataaa

atgaataattcggtgttatcgatagaaagggtcaaagtgcatcctattac

ccacgagttgggggtgatacgaatgtcatggttattctccgtcacatctg

cgtctgcacagcgccacgttgaatggttatggtttcaattggagtaggaa

caaaaaacatttctatgaatttgataaaatgactgtatgccatcatcaat

tagggatgattggttttctttttatttgagacggagtcttgctgtgtcac

tcaggctggagtcagtggtgcaatcttggctcactgcaacctctgcttcc

cgggttcatgcgattctcctgcctcagcctcccaagtagctgggactaca

ggcaccaccaccacgcccagctatttttttgtatttttagtagagacagg

gtttcaccgtgttagccaggatggtctcgatctcctgacctcgtgatcca

ccctcctcggccccccaaagggatgactggttttctttccaaatatgacc

tgagcattgatctgtccaataagcaagactgttatgtacattatgtagtg

gatctattgaggcatacgattttcctgtagcatttccattgaaccaatgt

ctagttctagtaagttctctgatgatatggttgaggctttcatggtcatg

ttcagattttatatatgtgtctcatgtaggaatctagtcttcataatctg

tgtcaaacctaagaatttgagtacagagggattctggaacactgcccaga

tttggcccagttccattcatgttttctgtgcgtagagataagttaccatt

aatgacatcaatcgtagttgtaggccatccctttgcctctgttgcttctt

ccaagagctacactggttgatcataaatatcagccaactgccaagggaag

gataccaacccttttcataacgccattttcctgaattgctcactcaagtg

tgtatgggagcaggttctaatattagttgtgattctttccatttatctag

aggattacgtagccaccaattgctttgcttagtagtgaggatggcttcat

gttgagcagtagatcttagaggatattcagtgtggataatgaaattccct

aacgaaaataaaagtgtgaaccttttgtgctaaaacaaaacaaaaacaag

tctgagtcaatagaagggggccacatggagtcttgtttcattgtcttggg

aaagctgtgtctatttcatgatatcatctacttttatggaaggatttctc

ttgggtatccttaattttgtttttatattattattattattattgagacg

gagtttctctcttgttgcccaggctgcagtgcagtggcacgatcttggct

cactgcaacctccacctcccgggttcaagtaattctcctgcctcatcctc

ctgagtaactgagattacaggaatctgccaccacacctggctaatttttg

tatttttagtagagatggggtttcaccacgttggccaggctggtctcaaa

ctcctgaccttaggcgatccaaccaccttggcctccttaagtgctgggat

tacaggcatgagccaccacacccggcctgttatccttaattttaaatcac

atgtggattgacaagtccaaaactctaagagagctctcattagctgtgaa

acatgtacccaaggttcaagatcctgacattttgctacggtgagaatagt

aactgaaactggtgtagtgtctttcaatggggctcaagggcagttgttct

ctgctgtcttttacaaaagagctagtctcggccgggcacagtgacttgcg

cctgtaatggtagcactttgggaagctgaggcgggcggatcacctgaggt

caggagtttgagaccatcctggccaacatggtgaaaccccatctctacta

aaaatacaaaaattagccgggcatggtgacacatgcctgtaatcccagct

acttggaaggctgaggcaggagaatcgcttaaacccatgaggtagaggtt

gcagtgtgccgagattgtgccaccgcactccagcctgggtgacacagcga

gactgtatcaaaaaaaaaaaaaaaagatccagtttccagattccaggtca

cgtaaggtgggatcatcaataggcaggtcacagaaagcctccttcacctg

gtgggatatactttggcatcatacatcaagaccttgcagtattgagtcac

atcagagccaccagtagagtggataaatgaagttctgttatgaaaggcac

aagtcgttcagtatttattttgtgaagggccactttatgtgttcccctag

gggctgatgttcttgccataaaggtcggtgataattcttttggccaaggt

aattcagttgactctgttaatttagctaattttagttttgtgataccatt

tctgcattcagcctttccagaagagagagtgatgagggcaatgatagtgc

cgtcaggtctgtaaactcttatttggtggataacctgtcaaataaagcga

attgccctagcactagagacctcctcggcaggtaggtgggatttcatctt

tctggagcctgagcatctccaaagaagagctgccgcatggagcttccctg

actggtgacctccaccctcttctctccagagctctttgacccagaggaat

ctgcaggaatggagggagttggccaaagtagggagatttttactcagacc

atatccaggtgagcacacaacagccttttgggcttgggctgatgcgtgca

tttctgtctgtacatgcgtgcctggaagtgtgcagatgagtgtgtgagag

agggtaatggccatgcatccttgcatagacaagaacatggggtgcacttc

tgcatgtgcacagcccctccccaacccgggcctgtggtgctgtcctgccc

ctagcccagagctggcctctgttggctttgagaccctagctttgcactgt

ggctttgctcggccttagagaccagctgctcccctgctgtggtctctcat

agtggagactgatccctggccacccattcagctgcctgagggccctggga

ccagtgttcatccccccacctcccaaaacagaggtgtgtaggggttccct

gcctggagtcccaaccctgcaggatggagaaaatgggcaccagttactgt

gacccattccggctccatatggatttggtttgacaacaaagctccctctg

tgtgttcccaagccccttacataaccaggggctgtgggcagaggaggaaa

ggaggaggaggtggagatggggaccaggagacaccaggatgggatggctg

gaaaggacacagggaccgggaggagagggcaggagacgagggccagagag

gaggagagggtgggagcaggagggggaagtgctggtcttgacctcccagt

tctgcctcctgcttcctggggaggggagggtggagtccagccaccgactt

ctgcttgtggatgctttgtcttcttttctttggacctttcccaccagtcc

tggataggctgaggaggggccgtgggacctgtctggggtgaggtgaggtc

aggtattgggctggacttgggaggctgttccttggccgcagcctgtaact

gaataaatgccattaaaaggggaatccatttcctccctcactcctggctt

ccattgccttcccccaggccccagccctggttccagaatccctctgacca

ctcctcttgtgctcaggagggcagaataactgcagcctttccctgtgccc

tgccaggctggtcccgggccccctcccagcccactcatccagggatgcag

ctctggccttggcccacccccatcagagggcctgtggccctgatcaccgc

actccccactctgtgaccagacgttctctcctgagcatcgctgaccctgc

agctgtccaaatagagcaagaagcgaatgttctctttggggcacagctgg

actggcagccctgcagggtcaggtgagctgccagtcctgggtgtgttctc

gggggtgggggtgggaggatcacgtcctgggctgagatcccttcagctcc

tcctgggcaggaggaaacagccatgcaggggcggccaggaggactgggtg

gcgggtggttctgggctctcccagcttcggttggggctgggaccctgggg

gacatgctctcctgggtgttcccaggctctgtgtctggcctgtgcctcag

tgtgtgggttttctggcaccaggcttccttgggaagagcctctggttgtg

caccagccctgagggtggggctcatcccaggctgcaggggactgagggcc

gagctcttccaccttgaggacaaggatggttcctcagggctgggaggagg

aggaggcgctggccaggaccttagattacgcagggcttggtgttggggcc

tcacagatgatggagaagccagagttcaggcacttggcatgacccctggc

atcgccttcagttgatttagtgtctctgccctggggtattccatgggacc

ctgggctggcttctgtgtgcagaggtgggtgtgggccatagcccagacct

gttgaaccagttgtaggctctggaatcctctgtccccaggcagtcaccag

gccttgacaggcgggaatctgtccctctcatcacccccccagttatgggt

gcagatgaccctgaaattgctaaagcctcagggcttgagcccagaaagtg

tgagcaggctggcggggcagctggaggaaggccttgcaggttcctggatt

tgggtgccgggtgggctgagaggacagtccctgtcactgggactgggcac

aggtgcagagaggctgagacaggcccagaggacagtgagtgtgtgttgtg

tgggtgccaggccagggagtgccctccatggggcagcaggctctgcagta

ccctcggccaaatggccacaggctgccctgggacactctccaggcctgct

ggggacacacctgtggggctcataggcgtggctgggtctcctctcaggtg

ccagaatctttctgggagctgagcctggctgggcagagggcgaggagccc

aggattcccctttccgtgactttggaggaacgaggttcctgagggctggc

acagccactccctgccccttagaggctctgggctgcctggtgacagcttc

ggggacctgcaagctgacagagtctccaggagccagagctggggtgggag

gaacccactgggcctgagggaggtagagcagcagcagcagcagctgagcc

cagcccccggccagccctgaggaatgggggaaaagagtgccctggcttgg

gggggccctcaagccccggaagctgtatgtgggcatggggggcgggggca

gagaggaggaggactgagaagtgggtgtatttgggcaggtgtgtgtgtgt

gaccaagcttgcgggcgcctgagtatggactagtgtgtttgtgctggagg

agcctgtgtaggttactgacatgggagggccggtgtgagctgcagggacc

ggagagctgctggaaggggctgtctatgccttcaggctctgagtgtggat

gcagctgggcctgttcaggcagattcaccagaggggctactgcgcagagc

ctgggccctggacacctagaagggcccctcctcccctcgtcttccctccc

tcatggcccacctttactctgagggggtgggcttcctgcgggggacaaac

tggagagggactctcttgccgccttctctgttcctggggacaggctgctg

cagggtggagggcttgcttttcctgcccaccctcatctttgccgagtcct

cgtctgggggccctgccccactctcccttcccaatgcagggccctctcct

ctggcgagcctgccttgatactccccggctcaacccctttaaccctgctg

gctcctaatgggactaatgggatttcaggtgtggacccttggaccctccg

ctgcacagttccagaaacttgttcatcaagtgccaggctgaccatggtgt

ccgtctcagtgcccagcagttgttctggtgatgggaggtgggagtacaag

gtctgccgagtgaggcagaggaaaggggtttctgagcccagatctccggt

gctccaagacactcaaaaccagagctgagagcctgaattcagaggctgta

ctgtggggcagggctggaatttgtggcacctgtgtagggactccagggct

gaacgttgcctttgctgtccctgcttggggattttggggtactgggtttg

gatagagtgtgtcatctgcagaggcagcactgttctggaagtggactctg

ctcaggtgtggcgggcctgcctgtggggtcaccatggacaggaggctggt

gtctggtcaggctgagggatccgtgtgccagcccttctgggaaggctgaa

tggggcggaactggtcagggcctggcaccaactccaggatgtgctacacc

cagacagacagatggagagccacctccctaggggcctcctccccctcccc

ttctgcatgccaccacaccccagcagcatctcctgaggctgggccatggg

tgaatctgagtcgagggcaggacagagtgggagtggcagtgcctctgcct

gtctccctggccgtggcaggtcctttgtcacatatgggccaggagaggtg

agtgtgattgtggtagtggtgggggcagtggctgaggacccctctctttg

ctgggctgggacagagcttgaatggagaggggccgccttgtgcctgggaa

gggaggaccaggtgtcgctggcaccttccacgtcctgagtgcttgttctg

tgcccagcccctgctgaggctgcggacattaaataccaggatatgttttg

agactcaggacacctctgaggacttgctgctgttatccacatttaaaaga

cgaggactctgaggctctgagaggttaagtacctggtccaggtcacctgg

tcagtttctttcttttttttttttgagatggagtcttgctctgttgccca

ggctggagtgcagtggcgatctcggctcactgcgagctctgcctcccggg

ttcacaccattctcctgcctcagcctcccgagtaggggactacaggcgcc

caccaccacacccggctaattttttttatttttagtagagacagggtttc

actgtgttagccaggatggtctcgatctcctgacctcatgatcctcccac

ctcggcctcctaaagtgctgggatgacaggcatgagccaccgcgcctgac

ccacctggtgagtttctgagctccagacaactcatcccaaatttcatggg

tgcatagagtatagagattaaaagtagttctggctgaattttaaatcctg

gttccactattttcgacctgtctgattttggaacatttaatgtattcctt

ctgtgcctcagttttcttctgtgtaaatgagaatgaaagagtactccccc

atcaatttgttgaaaacttcagtaagattatgccaggagctgcagcacag

ccccagggatgatttaaaagtgaattcaggacagggcgtggtggctcacg

cctgtaatttcagcactttgggaggctgaggtgggaggatcccttgagcc

caggagtttgaggccaccctagacaacatagtaagaccttgtctcaaaaa

aaaaaagcgaatccagtggtgtcactctttgttatcaatgccctccagtg

acattcatgacacccaaacttagaatctgagcttttagggtggcctgtga

catctataccacgtgtccccgtggcctccctggcaccccctttccttcct

ggctgtctgccaccatatggaactttctgtgccttgcacccagccagctc

agagtcttgggcacttctctcagtctggaaccttctcttgcctaccccat

cttcccatggctgcccttcatgtaggcctccggtttatagccggctcctc

agagacatggttctatccccccagtggccaagggctggtggccctcactc

tctgcccctgtgccctgtgttagtggtcctgtggttgtcattgccccgtc

atctccaccatttcctgtttgccgtgtgtctgtgccccccaaggatgaat

gccatgagtccagcatgtcaccctgtttctgtcctgccacagtgctcgag

tcagtgccctccctgcttgcattcggcccacaggccttgtggctcctgtt

tctgctgacctgtccagcctcacagtctggggaggccttggggtccccag

cctgtgctgggacaggggtattattccagtagcccttctgtggaacaagt

agggaaggagtgatgtgacctctgtggctctcctgggacacaggttagtc

ctgtgggtgacctcactttagagagggctggccagctgtgccggaattag

gaagtgggtacagctggacaaaaggtggctggagccagctctgcagaagc

cacacctgctcatgagaagggtatccttgtgagtacaggggaaatggcat

ggagaaagacaaacaccggtgggtggctctgccagcttcccctggggtgc

ttctggcaacgcccaggtctgtgcacatccagatctttgcactgcgctgt

tttgacttggagaggagaggaggaagttagctgctctgaatggtgttgct

gctaatctctacagcactttcctgtaaattaggaacggtaacacacacac

ccctcccccaccccgctggagaagccctgcaccagagcatacagtttggc

aagtaggaggagggctggagcccagcctcacttgctgccctggccaggcc

gtgattgcccagggtgcatactgtggctggtgctctgccactgctgtttc

tgtcctcagggtaggacacaaggagggcaagtagggccagtcagcgagag

gcgcaggtgacgcgtgttctagggtctgagatcagctgcttccatttcag

taattgtctttggagctacattgtctgctcatacaacttcccttccagat

agtgccattttaaaaatcccatttacccaaaagaaaacctgacacctgga

aaggtcgtttgcactcacacaagtgatggttggtccagaattcacatcca

agtgtgagatttcaatattttttttcttcctcctttttttaaatcaaatt

ttatttgaaagggtatcacagtcaccagtttgaaaaaggtaagcaggcca

ggtgtggtggctcacacctttaatctttgcattttgggaggcagaggcag

gcgtgtcagttgagcccaggagtttgagaccagcctgttcaacgtggtga

gaccccatctctccaacatttacaaaactagctggtcatggtggtatgtg

cgtgtggtcgcagccactggggaggctgaggtgggagggttgcttcagcc

tggtgggtggctgcagtgagctgagattgggcctttggtcttcagtctgg

gctacagagcaagactctgtctcaaaaaaaaaaaaaaaattataaaggta

agcagtacaaatgggaatatgagcataagtcactctcattccagacctct

gacccccaggacttttctcttgagaggccatagcctttgttttgcatcct

tctgagtaaacctattcgtgtacagacatatctgggtatgcatgtaactg

ttttgtgaaagtgcatttggagatatggatatcgttgttttcacccatcc

taagttttgatgctatggatactatgttttggagattagacgataccagc

acgcataggtttgcagcctctttttcacagctccatagtgtttccttgtc

tgtgagcagatttcaagttgtttccatcttaacccctagaaaaagtgctg

ttgaagatcctgacagacaagtctgtagaaacataggaaactttgtagaa

taaagttctggccatgggccaaattccctctgagaggtagctctagtttt

tcttcctagtacatctatgagagctcaggtccctcgtgttttgctgttat

gatgaatgaaatatgctgtctccatcacgctggaatttgtagatgtagat

gaccatagtttcttatttgccattcatttctttacattaaggtaaatatt

catgtcctttggctatttttattttatttcttttttcttttcttttttaa

gacagagtctcgctctctctcccaggctggagtgaagtggcttaatcctg

gctcactgcaacctccacctcctgggttcaagcgattcttctgcctcagc

ctcctaagtagctgggattacaggcacctgccaccatgcctggctaattt

ttatatttttagtagaggtgggggttttaccatgttggccaggctggtct

cgaactcatgacctcaaatgatccatccaccttggccttccagaatgctg

ggataataggtgtgagccactgcgcctggccctttgcctatttttaaatt

aggtttcttgtttttttttttttttttttttctgatttacgtgagcacat

cataggctcagtgaatttactgatatgttaggtataggttgcaaatgtac

tttagtttttcagtgtctttttttttttttttttttttgagacggatttt

tgttcttgttgcccaggctggagtgcagtgcaatgacacgatcttggctc

actgcagcctccacctccttgggttcaggcaattctccagcctcagcctc

ttgagcagctgggatcacaggcgcctgccaccatgcctggctaatttttg

tatttttagtagagacggggtttcaccatgttggccaggttggtctcgaa

ctcgtgacctcaggtgatccacccgcctcagtctcccacagtgctgggat

tacaggcgtgagccaccgctccccggctcagttgtcttttaagtgtttca

tttgtcttctctctactcctccctctctctcttgcacttgctgtctgtct

cacttaaattttatgtaggtgaatatattcatctttccttgtttagcttc

tcaatttatatttcttcactatctctattaatatgaaaatatctcatcta

tataaatattaatgtaacagagttgcatgtgataatctgccaggttgaac

aaatgggaatattatctctcatatgacgttagatttttttcctttaagaa

ataaaatattgcaagtagagaaccaaagacatgcccctgtgacctctttt

attcttccccatctccccagaatataattatcacttaaagttgggtgtca

acctgtatttcattctttcatcatacacacaagcacacactgtttataca

cataaataatgttgtttttggacgcatactttgtttttgcaacaccttta

aacataagtttttacttttattattttatttaagataggatcttgctctg

tcacccaggctggagtatagtggcgcgatcatagctcgctgcaacctctc

atctcctgggctcaagtgatcctcccaccttagttagcctcctgaggaac

tgggataatcacagtcataagccaccaagcccagctaagttgtttatttt

atttttttattttttaaataaagatgaaatcttgctatgttatccaggct

ggtctcaaactcttgggctcaagcaatcctcctgccttggcctcccaaag

tgctgggattacaggcatgactttaaaaagttaacattatgggccaggcg

cggtggctcacgcctgtaatcccagcacactgggaggccgaggcgggtga

atcacgaggtcaggagatcgagaccattctggctaacgcagtgaaacccc

gtctctgctaaaaatacaaaaaaaaaaaaaaaattagctgggcgcggtgg

ggggcgcctgtagtcccagctacttgggaggctgaggcaggagaatagcg

tgaacccgggaggcagagctgcagtgagctgagatcacgccactgcactc

cagcctgggtgacagagcaagactctgtctcaaaaaaaaaaaaagttaac

attatgtcttcaagtttatcccaattgacccatatggattgatttcttca

gtttacatggctatatagtattcaggtagcacagtgtatatagccatggc

tgtactgatgatagtgtttccagtttgtttgtttttttttgctattgcag

cactgcctaataaatatcctggagtgggttgtacttatatacttcaaaat

ggaattaccagattgtaaaatatgcacatattcagcctcactagatactg

tgaagtgctcttctaagcctttgtgtcagtttgtacttctgccagcaatg

tggaaatgagatttggttttaatatgtattccctggtcacgagtgagcct

cagcatcttttcatatgtccaccaagtgtttgggtttaatctgtgaattg

cctttttgtgttctttgctttttttttttttttttttatttctttgattg

ttttggtggttgttatggtaaaatgcacaagcacgaggttgtatttacca

tcttaactctttctgtgtgtatagtgcagtggcattaagcactttcagaa

tgtgctaccgtgctaccatcccaccatccatctcccgaactctcttcatc

ttgccaaactgaaacatcccacccactagtcaagaactcctcatttccgc

ctccccccagcccttggcagccaccattctgctttctgtgtctgtgagct

tgactactttaagtatctattctaaatggaatgatacaggacttgtcttt

ttgggactggcttatttcacttcgcgtagtaccttcaaggttgatccata

ctggtagcagaatttcctttctttttcaggctgaataatattccattgta

tgcgtagaccacattttgtggatccgttcatccactgatggacgcttggg

ggttgcttccacattagctattgcaaagtcaataatgctgctgtgaccac

gggtgtacagatatctctttgacatcctggtctcaaatattttggggata

tacccagaagtgcagttgcttgatcctatcctgtcccttgcttttccctt

tgcttccttgttttttttttttctttttgttttatgggagtgctgatata

ttcaagtatttgttgattgcatgcattatgcctgacttgctcaaagcatg

atgtcttgagcacttagcaaccttgtgcctgctgagtggtggaacagttc

tgaaaaggtctgttctggagtgggggtgctgtttcggagagcgatgaatc

aggggcttgggtgctgactttaggaggtgcggctcttggaaagacagagt

gtgacttccccagtgtggtctccttccttttcctcaccacttctcctacc

taaccccaagaatgagtccttgagtctgtctcagtaggtggatctgcatt

cagtgcagggcagagcctgggtggcagtgcctctccttgcctccctggct

gctacaaggtcctgtgtagcatctggtgtggacccagcagccaggaggag

agctgagtgtgattggggtcgtggtggggccagtggcagtggatctctct

ctttgttgggctgggccagcgcttgaattgagaggggctgccatgtgtct

ggggagggcagaacccagcgtcactgacaccctccagatacagagttctt

gctctgtgtttggcccatacctaatgttgtgaagtggaaatactggggtg

tgttttgaggctcagaacacctgtgataacttggtcgtgtgtgtgtgtgt

gtgtgtgtgtgtgtgtgtgtgtatgtgtgtgtatgtgtatatataaaatt

attttccttaacaccactgacctgaaggcagttacacgtaatttacaaat

gaggaatctgaggctttgagattgtcaaggtcacctgaccagtgactgca

gaggctggattggcctgtgggcctgagtccgtaccccttttcactggctc

ctcctggaggccacatgtaccttgcgcttcctggggcttggtcctggggt

ttcatctcaggttgacccccgttaggaggaagtaagtaggaaggaaggag

atttcctgggtattagggtgtcctcaagcctgcagctctcctgggactgc

acagagtttactgtcggacatcagttctgatatggtgcagcagacatgga

gctggtaagtggttaagatcagtcggacatgacaacctccagttctgcag

aagccacttcatttgcctggggataaagcaggtccttgtgagctcatcga

catgcatttggcagaaagtgcgctggctctgccagctcccagtgtgagct

tctagcaagccccttgtgccttagatgcccaggtcttttcagaataagcc

tggcttgttttaacttgatgcaacttgctgctcacagtcatcttattgtt

tgctgatgaattcagacctccattcatttaaatatttgtcagttatctac

catgtgtcaggcattaaatttagtgctggagatactatgggacataagaa

gtccctgcttgctgggagtgcgtggtctaattggtacttgggacagtgtg

gtttaactgccacaaagttaataaagcagcatgatgtgacagagctggat

gtgggggagtgaggaagcttctctggactgggtggtctgaaccccatgca

gggaggaaactgtggctgggaggagaggaggccggaagagaggtctgggc

ttgggtcccaaccactgattccagatctctcaacatttgtaacacgacca

actcctgttcttttttttttttttttttgagacaggctagagtgcagggg

gacgatcatagcttactgcagcctcgatctcctggactcagggggtcctc

ctgtctcagcctcccaagtatctaggactacaggtgtgtgccaccacacc

cggctaattttatttttatttttatagagacggggcagagtctgcagtca

catgccttgcaagtagcagagacagggtttagtctcggtagccagtgcct

agggcacaggtcttggccagaggagagaaggctcaggtgaaccagtttca

tctgacctgcttgctaactcaaacatggctgcccaactgcgtgggctggc

aaggtatgtgaggtgggtagctgggtggcccgccccttgcaggcactggg

gcaggatatcagggggtccagaagaggttgggtttaggcagagtagaggt

gattgtgtgggccgggggagcaatgtcaccttgaatgtgtttccataccc

agggcccagatggctcaggaggagggggatggggacggggagagtgaggt

ctttttgactttgatgtgctttgactgcacatcagagtcaaacgagtcag

aataagcgggacccctccacaggcatctcagccctcctaatttagccttt

ctgaagtggagccttgggagccgatgaagcttaataaactctcctggtgc

ttccagtaggcacttgagggtggacaccaccgtcttcaggtcataggaag

tgtgagctagcaggaatcctggagattttctagcccaacagtgtccggtc

aaaatagaatgggagccagaaatacaagccacatgtaattttaaattact

tttaaaacatttaaatttagccacggttttaaaaagtaaaaataggtgaa

attcgttttaatattttaacccaatatagccaaaatcttggcatttcaac

atgggatgagcataaaataatgaaggtgatattttattctttttttgggg

tactaaatctttgaaatccggtgtgtattttccacttaaagcacatgtca

gtgtggactggccccattgcaaatactcagtggcctgggactggggtgac

caagtgggatgtggtggggctggtgcagtccaccattctcacagcaggca

tctggggtcaggggaggcagggcactggctgtgctggaagcaggggattg

gctgtgctggtgggaagccagattccagccccagacactggtacctttgg

ctttagatccttggggtgggtgcattgcttctcctgggagggaggagcgc

ccgggagcccctgaccaggacctggggcaggtccacacaggccctggatt

tctcctgtatcctgggcctggccctgctctcttgactcccacctcgtcac

agtggcttcagctttttcaccctgagaagggaattcaaagcctctgcccc

tctcaggcaggcaccaggatatgattttcttttttcttttctttttgttt

gagacaggatctgacttggtctggagtgtagtagcctgatctcagctcac

tgcagcctccacctcccgggctcaagtgatcctcccacctcagcctctga

agtaattgggattacaagcacatgccaccatgcccggctaatttttgtat

tttttatagagatggggtttcaccatgatgcccaggctgttcttgaactc

ctgagctcaagcaatctgccctcctcggcctctcaaagtgctgggattac

tggcatgaggcacggggccctgccctgggtatgattttcaagtggttggt

gtcttgtgactggcagctgataaacgggtgatacaatgtttctgtacagg

agtctgcagtgccaggctgcaggggcggatggtggggggtgggtgggtag

cctgtggagcccgagggtgggaacagagagtcttcttttgctgtaggtgg

aagcctgtctggctcctttccctctaaggtcagacagcttgagagatgtt

tccatcctcccatgcatgaccctgaggcagttagctggtcagttactcac

tgtgctctctgccaactctaatgagccaggaagcctccccgtgcccaggc

gtgctgtgatcacagtgttcaatgatggggtgaggctacagcgtccagcc

aggggctgtgccacaggggccagaagatatctgacctactgtcactgcct

cttccactctgttccacctgcactggggcaatgccatctgatgaacagat

ccatttattaattcaacacgtttgtattgagagcctgctgggtgccgggc

actattgtcaacatgggtgatacagcagcaagcaaaacaagagaaacatc

tttgcaagcaggggagggggcctgcagtgttgggtgtgtggagttgttgc

tgggaagtagagaagctgagtaaagagctggacggagagagatagccagg

cccatgtctacagaaaagcattctagactgagtgagcataaatcgcaaag

gcccagaggagggagcatgcctagctagccccctagacctggtgaggctg

gtgtgggttgggcagtgatacggtttggctgtgtccccacccaaatctca

tattgaattgtagctcccataattcccatgtgtcatgggagagacctggt

gggagataattgaatcatggtggtgttctcccccaagttgttctcctggt

agtaagtgtcaggtgagccgatggttttatgaggggtttcccttttcacg

tggctgtccttctttcttgcctgctgccatgtaagatatactttttttct

tctgccatgattgtcaggcctccccagccacgtggaactgtgagtccatt

aaacttctttttctttattaatttcccagtctcgtgcatgtctttatcag

cagcacgaaaacagactaacgcaggcagcctgtgttgggaggagttggca

aggagggaggaggtgctggccagtagagcacatgtgtctctgtgcatcag

agcaaggacccgggctctccccgtgagtgagaagagtccatggggttttg

gggctgaggaggagttttacagatgaccggtctgttcttggcacttgcaa

ttagcggaccgtctgtatccgagtgttgatcgggtggattctaaccctgt

gggagaactccccagcccgtagcctgcgctcactgtacctggcagttgta

ttaatgacgtcaaatgcacagtccagcttgagtcccctctagaacgtggg

cagcggacactaagccgagaggaatcgctgtgggttggcagacactgtgg

agactgactcctggacaggctgtggaatgtgtggaagcaggtacagccca

ggcccgcgctgaggttcggcagggaagccagaggagtccgcagcccggga

gacctggcctacagcggagagagtcagtccatgccagtggtgggatgtgg

ctgcaaaaaatggtcggattcatggaagtctggtgtccagctggcaggca

ttagtggtcccatgaatacagctgtgtgccttgaacacgtactgcagagc

ttccagagagcaccagggctgtgggggctagagactgtgtctcgtgcgga

agcgtggctgagaaggggctcgacaggctcttcgtcactgtgcggagcct

tcgaaggacttggatgtgcttgagaccccttcccctaattgctgtgtagg

gcaggggtggacatcccggggatcgtggagtctagggagccagtctgcac

gggatggttttagaagccgtaggatgtggaggttctggctgaaggagcca

gaattccagtgccagctccactcctcacgagctgtacaacctgggccggt

tactgacccctttcccaccttagttccctggctctacaatggaggtaatt

atcgtgttgaactcacagggtcgagatcatatttaacactttcacagccc

ttagaagtgtctagcacagtggtcctcagcatttggaaatgtctggaaac

actttgtcacacctgagaagagggatttcctactggtgtctagtaggtag

aggccagagatggctgctaaacaccccataatacacaagacagaccccgc

aacagtgaactctctggcccctccaagtcagtggtgcagagattgagaga

tcctggtttcgcgttttgagcgtgcagtacatttcagttgttagtggaac

atgcaatacatctttattcacatgttattacttatttgtcagatgtttgt

aaaacatctgctacattcttggcagtttaatagaatctggagatacaaag

aagttcctgaccacagaggtcatttaggcaagctaggaaagcacagaatg

atataacacaaagttacattgtaccataaagcatattctatagtacactt

aactctggtgggctcttggtataaaaatgtctccaatctcagaggtcatg

ggatagtgggcaagacatgaaaaccttgtataatataaaattttgttacc

tctaagtgctgaaattaaggtataaacaacgtctcatgggaaccaaaaca

tcagtcatttggaagtataagtcaagccttcgtaggagaggaaccatttc

agcccgatattaccagagtgggatggtcccagcgagcgggatgtggaggg

ttaggatgttatcttagtctgttttgtgctgccattaccaccaagtaggt

aatttataaggagcagaagtttatttggctcacagttctggaggctcgga

tgtccaagatctagaggacacatccggtgagggtcttcttgctgcattgt

ctcatggtggatggaagaagggcaaggtggagagagagagcaggaagggg

cctaattgtccttttataaggaacccactcctgtaataatggcattaaac

ccattcatgagaacagagcccttgacccattcatgagaacagagccctca

tggcctactcttatgagaccccacctcacaacattgttaacatttaggat

caagtttctagcacatgaactttgagggacacattcaaaccatagcattc

tgccccagtccccaaaatggatgtccttgtcacatgtagaatacatttgc

ttcttacctataatcccaaattcttatttgtttcagcaccacattaaaag

tccaaagtccacagtctcatctaagtcagctgtgggtaaaactcaaggta

tggttcatcctgaatcaaattcccctccagtcatgggctgtgaaatcaag

caagttgtctgcttccaaagtacagtgatgggacaggcataggacagata

ttccattccaaaagggagagataggcaagaagaaaggggcaactggtcca

gacaactccagaacccagcagggggaacaataccaaatcccaacttgaca

ataatcttctttgactccatgtcctgccttccagacacactcgggtaggg

acttgggcccccaaggcctccggcagccctgcttccctggccttgctggg

ctcattccatgcagcagctctcaccggttggagtctcatgcttgcagctc

ccccaggttgctgttgcacactggtagctctgcagttctggggtgtcagg

atggccttactcccacagctccactaggcttcctggggactctcagcagc

tcagactccacagttctgctgggcattgccctacaggggctccctggtgg

tggctctgaccctgcaaccagtctctgcctgggtcccgagtctgtccgca

atatcctttgaaatctagttggagggagccaggcccatagcccctgcatt

ctgtactctggtggcattaggaccatgtgggatgccaccaagactcacca

cttgcaccttctagaatggtaggtcaagctgcacctgggcccatttaagc

catggctggggaggcagaggagcactgtgcccaaatgtgggagcagagac

ttgaggtggccctggacagtgagccacaggtcctgagggctcccaagacc

tggcttttgacatagttccttccctcagattttggcactgccggagctgt

gatcacaggtcatctctgcagtacctttgggatcattcttattttcttga

tgaatagcctctggcttcattcttattgtactaatctccttatcaaactg

tggctctggccaatactaatctccttatcaagtggtccatactaatattc

ttattaaactatcaaacggtaacatgcctgcacccttgctttcctctcct

gaaagttctttcattctctactacagggccggtctcagaatccttccagt

ctctaagttctgtttcgcttttcattataaatttctttctttctttcctt

ctttttttttttttttttttttgagatggagtctcactctgttgcccagg

ctggagttcagtggcacgatctcagtttactgcaacttctgtctcctggg

ttcaaaccattctcctgccttagcctccctaatagctgggatttcaggtg

tgtgccaccacacccagctaatttttttgtatttttagtagagatggggt

ttcatcatgtcagccaggctggtctcgaactcctgacctcaaatgatccg

cccgcctcagcctcccaaagtgctaggattacagacgtgagctactgcgc

ccaaccctgcttttatttataagtttcatctttaaattatttatttcttc

tcaagttttacagtatacagttaaaataagtcatacagcaaccaaaatgc

tttgctgcttaggtatttcttccaccaaatagcctagtttatcactctta

agttctgcctgctatacagtttgaggacctggacacaatgtagccacatt

ctttgctgctttgtaacaaggatgactgttatgccagtttccagtacctt

aatcttcagatatatctgagacctcatcagaatggccttcacgatcgata

tttctaccaccattctgatcacaaccacttagtctaagaagtttcagact

ttccctacagatcttctaatcccgctttattgcctgcctgcctccctccc

tcccttccttctttcctctctgctctctctctcttttcttcctttccctc

ccctcccctgctttcctctcccctctccacttcttggcaccaattttctg

tcttagtccattttgtgctgctgtaacagaataccacagattgagtatat

tataatgaatagaattttatttggctcatggttctggaggctgggaagtc

caagatcaaggggctgctctggcaagggccttcttgctgcatcatcccat

ggtggagagtggaatggcaagacagatcaagaggggacctaactggcctt

ttataaggaacccagttccacagtaatggcattaacccatccatgagagc

agagccccatgatctaaacaccttttattaggccctgcttatactgttgc

actggggatcaagttgcagcacttggacttcgggcaacacattcaaacca

tagcaggcaccttaataaaggtggaaaagggatgtggcagctacagtggg

agggaaaaggtggctcccttagtgtgcttgggaggagggtaagagatgcg

tttgcagttctcatggccgcagactgaaggggcctggttacctaagggac

cttggcttcatgtaaggagtgcggagtgagtgaagcaagagggtgatttg

atccatatcatttcttagaaggataccatgttggcagtttcgaagaaggg

gcatgagtgaggaaacagttgtggtgaagagaccagttaggaagcctgta

acttggggtgggatctggaaccaggctgtggctccggaagaggagctgga

gggctgcactgtgaagtatttctcagagtctagacaatgtgaccttcaca

ctggggctgagttttgtaaaatccgacatccccagtttcacctatgatgc

caggcctgtggtaaggagctttgcaagcatttgctagaatatgagcaaat

ggaggtagaactgataatatttagtaaccatggggggatgaaagagggag

gtgttgggagactgggttgataatatgggggttgggagtgaggagagagt

ccagctttgaacatcttcttccatttgaggaatttaagatcatccagcta

gaaaattagaaatactaggatggagtgtggaagagaaattagtgctgaaa

ataaagagctcgaatcatcagtgtatgaatagtagttaaacctctgaaaa

tgagtgaacttgtcccctgaaaatgtgtggcaggggagaagcgaggttca

aggacagtgcccccgagaatgccagcatttaaagggtggaccctcggtgg

ctcacgcctgtaatcccagcactttgggaggctgaggtgggcggatcgct

tgaggtcaggagtttgaaaccagcttggccaacatggtgaaaccccgtct

ctactaaacatacaaaaattagccaggcatggtggtgggtgcctgtatcc

cagctactagggaggcacaagaatcacttgaacctacaaggcagaggttc

cagtgggttcgtgccacttcactccagcctgggtgacagagggagactcc

ctctcagaaaaaaaaaaagaagaagaagaaaaaaaaaaaggtgggccccg

gcagcagagcctactgtcccggaggcaggaggaaaacctgaagggaatct

tgaccttgacctagaagccaaggaggaggcatggccaacaacatcaaagg

cctctgagcagcgcagtgaagactgacaatagacctttcttaagtttggc

aattagaaggtcattggcctcagcaagagcaatttcagaataaaaacaag

agtggagggagtggcataggggtcgggggacagagtaggaagtgagggtg

tagtagaggggaagaagtttgatcactgagggaaggagggagatgcaggg

gtagcttgaggaggagatagagcagagagaggtttgctgtttattttagg

aagaggagacttgagcatgtttataagtcagagaatagagaaggagaggt

gggagacacagggaaaagaggagtcattgattgggaactattctgggaag

gtgaggtggatctggtgtctaggtggaaggatttgggggaaagaggggag

gtaggagagtctagacgtagacagcaggcagacaggggctccaggttttc

cctacagaactgaggacagtgatggccgagcgtggtggctcatgcctgca

atcccagcactttgggatgccaaggtaggtggatcacttgaggccaggag

ttggacaccagcatgggcaacatggagaaaccccatctctactaaaaata

caaaaattagccgggcatggtgacacatgcctgtagtcccagctactcag

gaggctgaggcacgagaatcatttgaacctgggaggtggaggttgccgta

agctgatggcaccactgcactttagcctgggcgatagagcaagactgtct

gaaaaaaaaaaaaaaaaaagaagaagaagaagaactgaggacagtaagag

taagtggtcgcttgtgatcacagaaccagcaagtaaaccgcgagcttctg

cccctctggtcttcccagaccccttccccggaacattaagccatatcgct

tttatgggtccaggggctctgaggggctgggcatcatactgtgcccctgg

gggcatatctagttctgccccacttggttgggcgggtacctgctgagtac

tcactgtgtgttaggctgtgaatgctggggcagcaggaggcatggtttct

gcctccactgggccttatttcttcaccttgtcccccagttctgcccttgg

gtaagttctgtggcagaactgaccagctcccttcggccagggacatcttg

gttcctggcagagcaccaagaagagcagattgtcttcctacccgggggca

cctttgcccagcaggtcgggtgagctggcctcttggtgagaggctggtcc

ccttggacatgagtggattcgatgcctgctttcccacccaggtactctac

tgtggctttgctcctgccctttatgctcagagctagaccagcaatgtggg

ggagctgctggggaaaggcgccatgttgtcttgaggtagcagccagcccc

ggaagccctggaatgcctggggccactgagcaatcctcaccctcagcccc

acaaggggagggcgtggctcgtgaaattcctgattgggtatagcccagca

agccccaagactcgcccagtttgcttagcgcaacactgacgctgttttgg

aatgaagacaagacaatgtagaactttactggggaagaggtcgaagaagg

gctaattgtcttgttgttcaagactggttagcgctgctcacctctgacgc

tgctcacctctgacgccaaaggtttttccctggaaggcagccctggagga

**tacctgtgtatctgtctttcacaaaaggtgaaatgagctctttgtagtgt**

**ttcccgcccaggctgcattttagaatcacagaggaggtacggcgctgatt**

**tgttttactattccctattcatgcctgggccccacccctaaccaattaaa**

**taaacatctcggagagtggggcctagactttatatgtttaagctcctcag**

Promoter 3

**gtcattgcattgtgcagccaatgctgtgacctatgaatacaaaggaagat**

**gaaacgcaccctggtcatgcaaggaagatggagagagggagcagggaggg**

**tgagagaggctgcagttcgtaaggtcctgaaatgttagagctggagggat**

**cctggttcggagcactgcaactcaaaagagtagttctagccatgcagcag**

gccccattagggagctggttagaatgcaaatccccgcctgccctgcaccc

catctggcaccctctgagtcacggcgtgcacatcgaggtttgagaagtgt

gctttcaggtcacccacagatcatccagaccagtggttctcaaccttggc

tgcactttagaatcacccagcgggcttcagcgaattccagtgctgggcta

gatgaattaacccagaatttctaggtgttgggacccaaatactcatttaa

aaaattttttttgtttaagtacttcaggtgagtctcttcttcagccataa

tcaagaatatcaggcttgaccagtgcttctcaaacttggacagatagatg

aatcgccagggtatcgtgtttaaatgcagattctgattttataggtctgg

aaggggccaaagaatccacttgtctgggctctgggatgcccatgggactg

atctggccttttttgagcagtgggaataggctgacttctcagtttgacag

caaggaaactgcaggtcagagagggaggtgtgatcgtccacggtaaccaa

cctgatatgcattcagtactttctgatgtactctactgtgtgccaagcag

catgctacatgtgggactagagctgcgaacaggacagacccaattccaac

cctcacagagcttatatcccaactggggagacagacagaaagaaaacaag

taactaagctgatgacagatgggaaaagctatgaaggcgaggagggccat

gtgtagggaacgactcagagattgctgtggggtgaggggtaggtaaggca

cgcccctttcagaagagctgtcgggggagttactaggacaatacctttaa

ctggggactcctgctgattacaaactgatcacttttctttactggacagg

actaggcttcatcccctctggggtttggggatggtgcttctgactctcct

gaggcagtctgaccctggtccctgcctggctgtctcctctgactcaagac

tctgatccttcagtgtttgtgggttcagagcagagcctgggaatgagccc

gctggggagcaggacactgactccaccataggacagggctcaggatcatt

ggttaatggtttgtaaaatggaccaggcctcatccagatgctgtccccag

cccctatgaaaacatggaaagtttccactggtgcacaaacactagtgctt

gcctccctttcctgatcaaccgtcggactcacatgggctgtttgtgaaaa

tgcctgtcccaggccatactcaggctgaccaaggtggtctggtctccagg

gaagggccgtgaaatgtatttttaggaagtggtccaaaggactcttctga

tccagatcatcaggaagcatggcgtggatggaagaaactggcacttggca

tctggtgcctctgtgtggctgtagaagctacagaaatgtcttctgcagtg

actgcagcgtggccctggctgagctggtgcttgtgaggaagcctctgctt

gtgtgcaggggtgaacagcagctcaccccttcccatgcacagtcctcacc

ttactaccttcacccttactggaagcatctcttgacttctctttggccag

ccagaccctcccagctccctgtgctccccatttaaacaccttccctccct

gagcttagcccatggctgggtggggaggcgtgagcactgtttgcatttgg

tcagggcctagaagggatcaaggaggcctcatgaggaccatgtcatagcc

ccaccctcactccccacccctgacctgagcaaacctgtttggctggattg

atgatggagaggcaggcagtgaccagaccaagagacgccttggatggtgg

agaataggtgaccctctgttgggtaaaagaggactcaagccctcacacag

tgagttggctaactttttgtctcaacagtgggcgggggtagctgtctgcc

tcctggagctggcccttcctgggtcaaaatgcagaaggagatggtgagaa

gaaccacaccagagaggctggagtaggccagcctggcttgggaccgagtc

ccgagtccctggctggaccctcggttttcccttccgtggcagggctcacc

tggatccttaatgccgcccttggaggagttaggaggatcctggatgagaa

aactcaccctcaggatgattgccccagggagcagcttcctgctttctggt

ggaagggaggggcagacagtgggtgtgtcctgctccagtgtctaggcagg

5’ UTR

agagtttgtgaagctgaccggacacctgtggctcttatttcctaggtggc

ccgaggcagccgggatgacagctctccccaggaatcctgctgcctgctga

Exon1

gaaacATGGTCAGCAAGTCCCGTGAGTGTCATCCGAGGGCTCCCCCACCC

TGGAGGACAGGCCTCAGAAGCCGTCTTCAGCAGGATCCTGGGACCTCTGG

GGGCTGTGGAGGGACAGACAGGGAGCCAGGGGCCCTTCTCTTCATCTTGA

AGGACAGTGGGTACAATCAGGGTCAAGCCCTCAGCCAGGGCCAGGAGAGG

GCCAGAGACTGCTTCTGTTGAGTTAGGGGTCGGAGGGACTCAGAAGGGGG

CAGGTGGGAAGGTGGACGGGGGTTGTACCTGCCTGTTGCTGCCTCTAGCT

Exon2

CCTCTCTGCATGTGTCCTGCAGGCTGGAAGCTCCTGGCCATGTTGGCTCT

GGTCCTGGTCGTCATGGTGTGGTATTCCATCTCCCGGGAAGACAGGTACA

TCGAGCTGTGAGTTCACCTTCCATGTCCTTCCAGTGGCTCTTGTCAGGGA

CAGGGCTTAGGGATGGAGCATCATGGAGCGGGGGACCTAGTAGGGCAGGA

AGGTTCCAAGAGCCGGCACATGACCTCATCCCTTCAGCTGCTGGTACGGA

GTGTTTCCATGAGGGTGGGTGGGGGTAGGGCCTGGGATGTCTCACTGGGC

CCTCACCCAGGGAGTGCAGGGGCAGGAAGACCTGGATCCTCAAGGACTTG

GGTTCCAAGTGGAACTTAACTTGAGATGATTCCTCCCCGGCACCTTGGGA

CCTTCATGCCGTGGGAGAAGGCTTAGGCTGCCTGGAACATGGGTCCCTGG

GTCTGACTGGGGCTTCTGCCTCCTGTCCTTTTTTCTCTCCAGTTTTTATT

Exon3

TTCCCATCCCAGAGAAGAAGGAGCCGTGCCTCCAGGGTGAGGCAGAGAGC

AAGGCCTCTAAGCTCTTTGGCAAGTAAGTACTTAAGGATGAGGAGGGTAG

AGCAGGGCATGGAGCGAGCTGGGATTGAGGGTTTCACAGTGTGGGGAGTA

GATGGAAAGAGCAGCTGTGTTGGTGGACATGGGTTAGGTGAAGCCAGGGA

GGGAAGAGAAGGCCTTATAATATTAGTCATGGGCCTAACCCTACTTCTGG

CATCAAGATTAATTCTGTTCTCATCCCCACCCGGCTTTCACCTCTGTGCA

GCTACTCCCGGGATCAGCCCATCTTCCTGCGGCTTGAGGATTATTTCTGG

Exon4

GTCAAGACGCCATCTGCTTACGAGCTGCCCTATGGGACCAAGGGGAGTGG

TAAGTTCCTACCCGGCTCGTGGGAACCATGGGCTCACCCTGACCACGTTG

**Promoter prediction tools:**

Promoter scan results:

Promoter region predicted on forward strand (promoter 1)

Promoter Score: 54.89 (Promoter Cutoff = 53.000000)

Promoter region predicted on forward strand (promoter 2)

Promoter Score: 82.77 (Promoter Cutoff = 53.000000

Promoter region predicted on forward strand (promoter 3)

Promoter Score: 71.27 (Promoter Cutoff = 53.000000)

TATA found at 48431, Est.TSS = 48461

**For methylation analysis:**

**Bisulfite modified methylated DNA**

gCGttatttataggtagtgtttgttttggttttgtgtttattttattagg

Promoter 1

**ttttttttttCGtttttttggtatttaCGgCGtttttgtttgtttttgtt**

**CGggtttCGgaggCGCGaatattattttaagtttgCGatCGCGCGgtatt**

**attttCGttttttttttttttttatttCGttttttttatttttttCGgga**

**gtttttgggagtCGggtCGtCGtgtgCGtaagCGtgtttagttttttttt**

CGtttttaattCGggttCGggttttCGttttttttgtaggCGgattCGtt

CGtttttaggtCGgattCGCGttCGggatagggattCGgtCGagtCGagt

CGtCGCGttagCGttgCGtCGtCGgtCGgtgCGtttagCGgatCGgagtt

CpG island

gCGCGCGgaatCGtgttgtttCGtttCGttttattCGtgagggtgagtaC

GCGgCGgCGgtgCGCGggggttCGCGgggCGgggCGgggCGgggagtCGC

GgggttttCGgtCGtttgattttagtCGgCGtCGCGtttttCGgaggggg

tCGggttttgtaCGtgggCGtagCGCGggtCGgggtggggttgttatagt

tttgCGgagttgtttttCGgggtttttttttttggattagattttCGCGg

gaagtCGgatttttttCGtttttttggagtgagtaagCGggatagttttg

CGgaaagttttCGtttttaattttttagttttgCGttCGgattgaagCGg

CGgtttttatttttagtattttCGagCGatggttttttttataattattt

CGgttaggtttagtatttgggagttgattgtgttggaggtgataggtttt

gCGgggttCGtttgtgtgtaggagtCGtaaggtCGttgagtaggatttaa

aggtggagttgttagtgggaggtCGgttgtgtttagggttttagggttat

**Primer set1:**

F1: CGgCGtttttgtttgtttttg (TM=59.2C)

R1: CCCGACTCCCAAAAACTCC (TM=58C)

Size = 139 bp; 11 CpG sites

- Methylated working at AT 65C, unmethylated very weak at this temp, trying extra magnesium, improved strength of bands slightly.
- HRM didn’t work, this set needs to be redesigned.

New R1: ACGTACAAAACCCTACCCCCTC (TM=59.1)

Size = 488 bp; 68 CpG sites

**Primer set2:**

F2: TtttCGggagtttttgggagt (TM=58.6)

R2: CGCAAAACTATAACAACCCCACC (TM=60.4)

Size = 409 bp; 62 CpG sites

- PCR did not work at all.

**Primer set3:**

F3: CGgggtggggttgttatagttt (TM=59.8)

R3: CGACCTCCCACTAACAACTCCA (TM=60)

Size = 345 bp; 19 CpG sites

- Primer set 3 works well for both methylated and unmethylated template at AT 65C.

**MGAT5** (2q21 +; [chr2:135011830-135206468](http://genome.ucsc.edu/cgi-bin/hgTracks?hgsid=167755531&db=hg19&position=chr2%3A135011830-135206468)) 194 kb, 16 exons

No 5’ CpG islands predicted in UCSC Human Genome browser within 10 kb +/- TSS.

No CpG islands were found using methyl primer express or CpG island searcher (5KB upstream of TSS)

No articles in PubMed on MGAT5 promoter

RefSeq gene: NM_002410

cacatatcataccatcctcctatttaaacgtatacaactccatggctttt

ggtatgtccacagaattatgctcctatcaccacaattttagaaccttttt

gttaccccctagaaaaaccctatacaccttagctatcacctctcatttcc

tccattctccataccccagccctaagcaagtactaatgtattttctgcct

ttacagatttgcctattctagacatttaatatcaatggaatcatgcaata

tgtgtttttttgttactttttttgcttagtataatgttcttaagagtcat

ctatgtgtagcataatcagttcattcctttttatagccagataatattta

attgcatggatatggcagttttgtttccatttcctcagttgatgcacatt

aggctggttttacttttgtttattatggataatgctgctgagtatggata

ctgctgctgagtacttctgtgtacaagtttttgtgtagacacgtattttc

**atttctcttgggagagttcctagaagcagaattgtttggatcatacagta**

**actccatgtttaactatttgaggtactgccagattatttcccacactggc**

Promoter 1

**tgcgccatttgatattcccaccagtggtagatgagggttccaatttctcc**

**acatcttcaccaacacttgttcttatctgtatgtccttatgagtgtgaag**

**ttagtatctcattgtggttttgatttgcatttcctctaatgactaatgat**

gttgagcatcttttcatgtgctttttttttctttctttcaggagaattga

cacatttctttgccagagatctctaaccatctttataaaggttgaagatg

attttgttgtgttgaggcgtcagtgggaaggaacaaggtgattgctactc

ttttagaaagtaggaggatagaggggatgcatattgaggtggctaattct

gcaaacagacttggtgcaaacttttttctggaagtgaattgatgaagtca

cggccatgagctgacattactctggttggcaacaaggtcttctgagatcc

actgtatagtcccactgtgcccattggatgacttagctagaggacacagc

cgttagaaggtctggaggtaggcatcttcttagctccagcaacaacttct

tgatcactgttagtctagggcttcctcattaattgtctggtcattcctga

tgatcttcattgtcattttgtggcaccaagactatgaggatggctgttca

ttttcctcctcattgtccacctgttactgtgagagataggggtgtctcct

gaataatgcttttgaaaagtcttaagttctgtcatcttaataggaattgt

agagatgcattctgtgattctctgtgtgtgctgtgtgtatactgatattg

tttctattatttcaagctctaagtaccactggagaagagaagtttcctgg

cacttctatagtcacttaaaatctgggccctcactaatcagaactgtact

ctgaactccgcatagctagggattttcacttcaaatttgcatgtctctga

actgggataattttacctaggctgagattttttggccatctctttgcgcc

tctgatacaccatctggatgtgtctgaccttctccaaggcaagggcagag

aagaagccttctaccttctaaggaagctattgttcagttcctagcccttg

atctttcctttgttttaactccatagcttgagcagcctattcagtagggt

taattgaggaagtagagtggggtttcctgtctgaaagtggacagctaatt

cctaccagtgattcgggctctttactaaaatagtgaaagaaaaatcacct

tttttgatgtagcacctcattggcttgctctaggaaaattccttcagccc

tggttcaaaattaagtaatgagtaatgagtatgggttatcaaaaaaaaaa

aaagccaattcccttggagctaggtgcagcacctagttcgtaatgtggct

gtcagagtgtaattctgatggggtggaaactccatcttgttcactgttca

gtcaccagggcctgatggccgctcatgctcaatatagacttggcgcggag

cggagtggaggaaggaaagagggcaggtgctagttggctggcctgcagtt

agaagggctgaataaagtgctgtagtgccctaggcgacatattcatttag

cagatactttattgtgccactgtggtcagtgcctttctctgagaaggtgt

gtgtgtggggaggggctccctaaaggatgtcatagagtcccacatagaaa

ctttgaacaatccaaggtagacaggtgtttttaactcataatactccttt

attccctgttttaaaaatttttttaaatttgatacaataattatacataa

taatggagtaccatgtgagattcaatccacatatacattgtgaaatgatc

aaattaggatagttagcatgcacatcacccccaaataattattacttttg

tggtgagaacacttaaaattgtctcttttagaaatatacgttattattaa

ccatagtcaccttgctgtgcaatagaacaccagaacttattcctcctaaa

tgtaactttttacccattgaccactccctcctcatccccctctctcctcc

ccacccctggtaaccactgttctgttatctcctatgatagcaacttttta

gcttctgcatgtgagattgtacggtagttgcctttctgtgcctggattat

ttcatttagcataatgtccttcgggtatatccctgttgctgcaaaagaca

ggatttctctctctttttctggttgaatagtattccattgtcagagaagt

gttgtaagactaggaaaggaacactgcaggctggagccctggggaaagtg

gtctgaggcaggtggtgggactagagctggggtctggcaaacaggctggg

tttgattgtcagcataatagagagcactcatgtgccagctgggtgggagg

agcagccgagtgaagaaggggaagcctctcaggaagcatgtgcagggttt

atggtaatgagcagaccagcaggtacgtagtgggagaggggtgtgatggg

gcagaggaacttacgttatgatagtacaagacagaggttgagcctcattt

taataggcattgtggtgggtgttgaatagtgatggaatgtatgggtctgg

aatcaggctgcctggtcaagggctctgaaacatgagtgtgcatcagaatc

acctcgaggcttgttaaaggataggctgtggaccacatctcctcagttgc

tgattcagtgggtgtgggtggggcctgagaattcacatttctcactggtg

atgctgctgttactgagtttgggaccacatttggagaaccactggtctag

aattgagaggttggcaaaccttctctgttaagaggtagatagtaaatatt

ttaggccttctgggctacaaagagtatctgttacatattttttattgctt

ttcatgacccattaagcatatatatatcattctctgccatatacaaacag

gctgttgggggagtgaggatgatgtagggaaggtggggcatggtttaata

acccctgggccatgcctagatgatcagtcctctgccacatagctggctga

cctttgccaagttaatcaccttttacctttattttctcatgtttctaata

aaacagagacgataatattcatacttcttaccatatagaacttctgagga

ttcagtgagcaaagccacaaaagatggtatgtcacaatatctgggatata

gctagaatttataatttatttttactctgttgataggcaatgggaaaaca

gtaagaggcagaccaacagtgatccagggctctgaaagctaattgcttca

agatcctgctaccattttcttttgggccgcttgcaaagaagaatcctttg

actgaagcatgtatgtacactctgaagtacagcctgggttagtctcttat

aagggatcggatcattgctcagcctctcccttgagtggcacttagaaaat

ggcgctattcgtaagctgactggtattgggcccaggactctggctgaagg

ggtgggcatgctggtaaccatttgcaacctatgctcaggtcctacttgtt

gggaagccctgattgagaagagtggcctggtctgtgctggcattagatag

gatctggctgcattaatattgaaactactctgccttttaatgtctcattt

tgcctcatggtgggagtgaaagtgagaaccacagaaaatctgcctgccag

gtgttccacatttcttgtgctacagcatgcaagtgagcagtgaggtgtac

cttttcctcatgtagctgggaaagcaatacccctgcttgtacctctggca

tatcttctctgtgctggtgcacctagagaggttgcctggtggccctgaga

gagccatctcatcactaaacactgatggtgaaagctggccatgctcaaat

aagatgtagcaatctacctcttctttgtctagttacccccaagggggcat

ccactttcttgctcacctcaccagttgcatgttctagtccttgccagaag

cacataataatgactttgtaagcttaagttacaggcacacaaaagggcct

gatggtgatatgactccaccctccccgtttttgctgacattccgccaaat

atccttctgtctcctccccaccttgcaaaacaaacttcctgttttgaatt

tggtccaggctggaacagccccactacacctgttaacacacgcagacgca

cacttcccccttcataattgcttagcttcttgttgcctagccagatttcc

cctcagcttacagttcctgaatcataagatattgaaccagcaaatttaag

agttgacattttacttagaggtattcaagtgaaaacatggcttctggttt

attttgctgtattgtgccatgaccacttggctaattcttctcctccttca

5’ UTR

cagcagaatggaagtgaggaaaggcaaccagctgacacaggagccagagt

gagaccagcagactctcacactcaacctacaccatgaatttgtgtctatc

ttctacgcgttaagagccaaggacaggtgaagttgccagagagcaATGGC

TCTCTTCACTCCGTGGAAGTTGTCCTCTCAGAAGCTGGGCTTTTTCCTGG

Exon 1

TGACTTTTGGCTTCATTTGGGGTATGATGCTTCTGCACTTTACCATCCAG

CAGCGAACTCAGCCTGAAAGCAGCTCCATGCTGCGCGAGCAGATCCTGGA

CCTCAGCAAAAGGTACATCAAGGCACTGGCAGAAGAAAACAGGAATGTGG

TGGATGGGCCATACGCTGGAGTCATGACAGCTTATGGTAAGCACTGTTTC

TGGGACCTCTCCATTAAAGTGTGCCTTGGCCTTGAAATGGCCCTAGAAGC

TCCCAGATATGGTCTTGTCATGGACTGAATGTCCTTTGCCACTGCCTTCA

TAAGCAGTGAATTGCACACAGAGAGGCATCTTTAGGAAATTAAAGCAATG

CCATTTTGGGGGTTCTGAGCTGGTGTATGCAGACACACTTTCCCCTGGAG

TCAGTGGGTTAGTATAGTTAGGGCTCTATTGGTTGTGTTTGCAGCTAGCT

**Promoter prediction tools:**

Promoter scan

Promoter region predicted on forward strand (Promoter 1)

Promoter Score: 91.20 (Promoter Cutoff = 53.000000)

**GATA1; chrX:48,644,982-48,652,715) +; NM_002049; 7.7 kb; 6 exons**

No 5’ CpG islands predicted in UCSC Human Genome browser within 10 kb +/- TSS.

No CpG islands were found using methyl primer express or CpG island searcher (10 KB upstream of TSS)

**GATA2 (**[chr3:128198265-128212030](http://genome.ucsc.edu/cgi-bin/hgTracks?hgsid=167818196&db=hg19&position=chr3%3A128198265-128212030) (-))

**NM_032638, 13.7 kb, 6 exons (5 coding)**

2 5’ CpG islands:

[chr3:128205496-128212274](http://genome.ucsc.edu/cgi-bin/hgTracks?hgsid=167818196&db=hg19&position=chr3%3A128205496-128212274), 514 CpG dinucleotides, 6.7 kb in size

[chr3:128215213-128216905](http://genome.ucsc.edu/cgi-bin/hgTracks?hgsid=167818196&db=hg19&position=chr3%3A128215213-128216905), 137 CpG dinucleotides, 1.6 kb

gaacactctggaacaagaagagagtcattgtggctgcagtgaggaagaag

gaaatcaggtctgagaggtcagtaagggccactacataggttctaagagt

ttggcttttattctaaattcagtgggatgcgttctcagaaacgatttttt

tgctgccaactaaactgtagcctttggcagtttgcccccgtctataattt

cctgcccatccatccatccatccatccatccctccaaacatcctttcaag

aaacacttatggagcacctactgtgtgttagtctggccctggggaacagc

agtgacaaagcaaagcccctgctcccagagagcccgccttctggaggtag

**agaccctcaccccacctcttctgcacgggcaccggctccgcggaccccac**

**ccgagaccccgccgccgccaggtggcgctctgcgcctgcgcttgggcagc**

**agccgggggtcgccgggctggggccggggccggggccggggcaggggagg**

Promoter 1

**ggcggccggggagtgggggcggggcccgcgtcgcaggttgtgtgactttg**

**ggtttgacctgcctggagatgagctaatcccgccgtataatagactctgg**

**ggagatagggaaaatggctgcggcgctatctgcgtcgccatggggaccat**

cgcgcgcgcgcgggcggggcgcgagcctggaaaaatacagcctttgtcgg

cggcgcgtgtcactaacggcgcggggacctcaggcggcgggctcgggctt

ccttgtcccagctgcagaggctctggggctcggacgcccgggtgtcccca

aactccgcggacggcccctcttccttccacactcgcacattccgttttgg

tcaagaaagtctcccgtgacacagcacgttccaccccgccgctctggcgt

ctgtcacaccggcacacaccattccctgtcgcttccccagcccggagtcc

acccacgctggcacacggacaggccccgacgactgcccttccccgtgccc

CpG island 1

ctctctcagctcaccagggcacacctgggcagagatacggcaggacacac

acagcctcacgcagacccaagcacaccccaagcacgcccggcgggagcgg

tgacacttgcatgctcatcccggtaggcacaggtagcttgtgtgccccga

aacacaacgtagggtgggcacttgcatgcacacagcacatccaccctagt

ggcctccggcgtgctcccctaaacagccacctccagggcagacacagtga

ggcactcgcaggatgctcatccgctgcactcggacacgcgggcttgcatt

cccattcctccaccacgtgtccagccaccctgggccgcatgcctgcccac

aagcttagtgcagcaccgcctcctgtgtgaaccctccacccgcctcatcg

gccgcctccggacacagcacagacgcctgccatgcgtgtgcatggatggg

ctcgcacacctgcacttttttgtggtacacttgtttgacacacccgcaca

gttgcatcggcgttagcccatgcactggcacacccacgtgtccatccaaa

cacgccgggcacaccggggcgcacactcagtggccccggcaggcaggcga

gtggtacgggcacggggagcccgcgtgcccccagcagagggctatctgcc

tgcccatcagcgctcggatctgcggtggtaaatgatgcattcgatggcgg

gcgcatttgttgtgcgctctctgaaagggctccgataatctggaaggcag

agataaggaacaccatttattccgcggcactttggaaacaattcccagcc

ctgtacaaccccattctcgggcagcctccgggagccgggcagataacgac

tggctattcattatcttcgccgggaacaaagattagccggccgagatgaa

aaattaccccggacagcggcgcagccccgcgcggagccccctgcccgcca

ccaccgcccgcgccttggtgtcccgccctgcccgcggcccgcagctcatg

cccagcccatcctcgcccggcgcccggcctcccgcgcttccttcgagctt

ggcggctaagccctgtcggcctttccatctcagtctgtccatcccatcct

gcccatctttcctttgcgtccccactcttctctctggggcccttctcacg

tccctcttctcgcattggctctttgcgcctctctttgaatctctctattt

ttgtcttatctctctgtcctctcttatctctctcctgtctctgtccatgt

ctctgtggttatcaccttctttgcttttattcccatctttctgacccttt

ctgccactttttagttgtttatttctgtctgtctccaagtgtccccaagc

tccaggcctgtccccaccagtgcctcttcctcattgtttttttaaaatct

gtctttgggcctcccatctctttccccactccttgtctctctggatcttt

ctgtcactgaatctccatttctcttcatccaccccaccctgagggcccca

catctccaggcctcatacctcctgccctctcaaggctgtgaacctggcca

cagaccagagactggaaggctccagccagctctggcctggccagcccagc

tccgcccagtaggcaggaggcaggtggggtggggaggtgcaaggcctgga

accccctttccaaggaaacaagacagaagtagaaaaggccctgtaggcac

ccaggccctgctcctgtgcttcagacaagctcgcccatgcccctccccag

tgaggcccagggccttgggatcgggtggtcccaatgaggacacctcatta

gagcagttattcagcatggcgctggcgacagcagataagccttatcagct

catctcccctcccacagacagacagaaagtgcccgagcaaatggtaccct

agagctccactggcggcctgccctgctggttaaggaaggtctggatggtg

tgtgatcctgcaggatgcacaccccgcgtgtcacccaggacacacttttc

tacagtccacagaacagggcactgctggccacagaaacatgcatttcaaa

gatgtagtcatatacacagttgcccacaacccagatacgcagacacaaac

acccaccgcaacacacatcaattcatacatggagctgtacgtagacatgc

agacacactcggatacatctcctcctccccaccatcagttcaaagataca

atgatatacactgatacctatgctgttaaagacagatggacatgcacata

gccaaatgcatgttcaaatacacaaacacaaacacacgccccaacagcca

tcaattaataaagagaattatacatggatatccagacacatgcaacacac

cacacacacacacctacacatacaccagttcaaggacacatgcagatacc

caggtacaaactattaaagacacacacaggctaggcgcagtagctcacgc

ctataatcccaggactttgggaggccgaggtgggaggattgctttagtcc

aggagtttgggaccagtctgggcaacatagtgagactgtgtttccacaaa

aataaaaataaaaataattagccaggcacggtggcacatgcctgtggtcc

cagatactcaggaggctgaggtgggaggatggcttgagtctgggaggttg

aggctgcagtaagcgctgattgaaccgctgcacttcagtctgggtaacag

atcaagaccctgtctcaagaaacaaaacaaaacaaaacaacaacaacaac

aaaaatacacacagtaacacatgcctagatacatgcacatacacacgcaa

acacacactccaaaacacacccattcatagacatgccacacactgctaca

gacacatgggcaaacacctggaaatccccacccctcattacatgagtctc

tgacatgatgaccccacagacacagagccaaagggacgcacaaccatttc

cacagacacactctcttgtgccaaattccagggtcatgaaatagccacac

tgtgacctactgcccaatacacaaactctcaggaacccacaagaagaaat

gaacatactcatacacccagttttgcagacactcctgtgtgctcctaagt

gcacacagacacacctgggcacacaggacatagactctgagccacatgca

tagaacacacacacaatgcaatagtcaccaccagatttggagaaagacct

atggacagcaggggtcacaaagaccaggggacagaaacactcctctgggg

cacacctgggcacgggagcacagatttcccagtccttccaagcccaagca

ctttccctctgggctcaaggtccccaggccactttcactctccctgatct

gctggaatgggaatcaggggtgggggtggggggacgctgactggttgaat

cccttagtaagaactttggcagcacaaggactggactgtctttgcccaca

aacccaggggccttctggggatgaggactcccacactcactttctccccc

cctcctgactcccacttctgtgaacaagccctctgcacacttccttgctt

cccccattgtgagagtctgccccaaggagatgggccagggggacaccaga

cagtggacttctaaagtgctcagggggtcccccagcgttaggaactggag

ggggggtgggacagggagaggggctgcgcctgcacttagctaaacaagcc

tggcagtgagggcgtcctgagtgtgagcccgagtgccgagttgcatctga

ttgtatggccatgtgtgtcggtgtgacccaggccgtctgtctgaggatgt

cctcgcggccacagctgtgtgcacatgtgcagggatgtgtgcgtgtccgt

gtgagcactcctggaagtgggttgaagacagaggagcgggcgtctgtgta

ccattatcagtgagtgcgtgtgctcacagctcctgagggtccccggggag

agtctcagtaaagacgtgtcccaagctttctgagcaagcatccctgaggt

caggggcgcgcgtgtgcgctccggagagggtgggagcggcctggagtgtg

gatgcacagggtgtgcgcgccctgagtgcctagcggcccttggccggcca

ggcggctggacctggtaggccgggtgcagagccccgggtaccctcctgcc

CpG island 2

ccctgcggcgctcccgcccccgtcgccaccccctgcccccccgcaaagtg

atgtcgaaataaaaagccgcgttgcggccggtgggggagagagaggcagc

gtgagcgccaggaaggtagcgaggccagcgtcgccccgggactcgctgct

caagtctgtctattgcctgccgccacatccatcctagcagggccccgtcg

cccaccaggcggacaaaagcggtccgctgaacaccatgcggccgctcggc

5’UTR

gtgccgcccaggctctgctggtgagcgccgccaccccgcgcccaggtccc

**gcgagcccgcctgccgcgcacctcgccctgctcccagctctactccaggc**

**cccgtccgcccgggggcgccgcccaccgcgcctcgctcggtgagtttctt**

**ccacttccaccttccctgggcccggcccttcccgcccccggcccggcccg**

Promoter 2

**cctggcacccgaatcgcttggtccgtttgccctgtggcccctacctttgg**

**ggctcgccttggccctgccagagaccggaaaccctggttatagggacttt**

**aaccagagggagtatttggttacctgggcacagcaggcccgcctcgggcc**

tcttgtcttcccatttctcggagccacaggccacgagaccctggcattcc

cagagcctcgcaaagcacccccgcctcccggcccccgaactggggccttt

gtctcggccgcccccaatccccaggccaccgcggcggatgcgtccgagcc

gggccgccgagcggcggctgcacctgccggctggtcccgtgcgccggctt

**ttcgcggcttaacccagctcgctcctgcttgcgcccccgcgcgctgcgcc**

**ccgcagccctttatcctgtgtgactggggtgcgtgtgggggagagcgccg**

**ggtctggaagtctccccccgccccaagccggagtcggaatccgtttagtg**

Promoter 3

**ggatttcaataagaatggggccgccgcgggcttgaagatctgccgccggt**

**aggcgtagacggcgttctggatctgtctgaaaacggggcatttaaaatta**

**ttctgtggcggccaggcttgaactccgcgttgctccaaccacgaggggaa**

aggccccgcgttcagggaccccgtgtgtggcgaacgctcctttaccggaa

aacaaggaaggaaattcgttgttttggaaaaagcctcctctcccgaggtc

gggaacatctggtggtgagctccaggtctacgcaggcaccccgtgctagg

attcgtttatgagcaggcaggattcgagaaccaggtagggcccgtgcggc

agccgaggcgtctggggagcgtttcccagccgggctgacaaattgcagac

aaattgtgcctaacgaaacggatttaccagttgtcaggccgccggccccg

gccgctccaaataaaccgtggctgttcgtggaggagggagaagcacggcc

cgattgtctccgggtctcagcagggttccgcggggagcctgccaggcttg

aaggtaggggtcagaagcgatatagaatctcggaggcgcctgggtccagg

gtgccgagacacctaggacgtgggggccacagactctacgattcccaaag

acacagaacagtaatgaggtgggagagcctgcactgatgccgagggaggg

agccttctgctttaaggggctggaattgaacctcaaggagcaggaggggc

cgttatagcagtagtcccccccttgggaacccccccggagggatggctgc

tggcctgagatctaatgccccggctttaagggaacttctgaacccatctg

ctaaggcaccccacttcctccccgtacccctcaaggtttattgccagtgt

ggggctgggaggccgctgggttgcgaattaaatttctctatggaaggtag

tcccttagcaaatgggtttccttgacaccccacccccagccccacaccgc

gggccaattagctgcccataaggaaaaggcgagaagaattaggttacaaa

gggagggcaaacttatggtcccaaaggggccgcctcggatgagctaactt

taaacaaagggctcagaggggggggggggctggacggccggggagacctg

ggcatctctgtgtccccacctggcacccgcggcttagtagaggcctgaga

agcactctagaaccgggcaccagatctgctacctccccagctcccaggca

gaagcacccaggtcaaatggtggcgatcgccgctgtgagttctcgggcca

aaagggtccttcgaaaattctgcttcctggctgaccttctccagtcctca

gagaaatcttgttcccaagtaaggaaagtgacagcttcttaatgtgatca

aaggcagcgccagcatttccaactatactcccgaacgaacaaagtactga

aaaagggaacgcgtccctctaaaggtgttttggggaccccaaagttccag

cccataaattggagtaaatctgctctcaccaggcctggaacagcgcctca

agaccccagcagattctgggggctgcgttgaccctccccgggagtttgtt

tggggcccaaggtgggaggaccatgtcttcggcctaatggggaggggccc

ggttggtgtccctcggtctgcctggcacacacagacattgtcgagcgcgg

gtccctctttattggccagctgggcgccctgctacttggcgtcgcatttc

tctctcccaggcgggttcgtttccgccagagaatgcagcagtcccgcatc

ctacgcaggacctgcaaccgaggtggaggcttcggtcaagccggctcctg

cctgcgttgtcgaggaaggcaaccccaaggcctgaaaggacctggcagaa

ctcctgtttcctttttttcctctacaccggattgcgggcaaggaggctgg

ttcgggtctcccgaggcccctgctcaagcactccttaaccgtcctgctaa

gcccctctgtgcggcgattttctgagctgccgagcggggtaattaaatcc

cctccctcgctccgctctgcgtcaggcaggcggcagcacagggctgacgt

ttgggcaggggactcagccaggctggccacctccactaccgcagtggccg

ggaccctgccgcggaggggttagacgccgagctcgctgcgctgaaactgg

gaatacacacggaacggggagggggagggtaatttttaccgcgccggtgg

gagaaaaaggcgaattacctgctttcccgagggacgcgcgtagccacttc

cctgagagccgcggcaccgatcgcggccgggcgggaagcttccgctcggt

cctggcgttcacagcccagcggccagctgctggtctgcctcccgcgctgg

gtcccagggttcgcgctcgagcggggcagctttgccggacacgggggatt

atccctggggacgcggtgtctttcagagggtcttgctagtctccggagac

gccaaataggctcgagctccgcggcgatctcattttacgagttgatgaag

aatcgtaagctaaaggatgggaaaagttgagagacagacggacggagaga

cagtgggccccggcgggaccgcacgcgttgaggggaacgccaaccgggag

gcacggagactgctcacctgcccggcctggccgcggaggcccggcgccaa

gggcctcgcgctcggcctcccgccccctgcgcggcttcccgggctggcgc

cggcctccgctcccgcagagtggagttccgagcagaccgggctccgcgcg

ctccagcgtggaggggagcgggaggcttagcaggcggctcgggcaggcgg

**gtcccccaagggcacgagacgcgctggttcccagcccaatggagctctgc**

**gccccccagccccgcgctttacctgcgctgaggcctcggacagacaaacg**

Promoter 4

**gacgccagacgcctaggcaggaggaggcctcagcctgagcccgcggcccc**

**ttggcgctgccctgaactggcctgggagggggtgacgggggcgcgcccgc**

**ggagctgggcccagccgggcgcccccggagccgaggggaccgagggcttt**

**cctccctcctcggattattaaaaagttcatttcctggcgaatcgggtgac**

gtcaggggctcggcgtcgcggtggcggggccgcccggccggagaagccgc

ctccagttacccaattaccgactgtcaatcccgccgcccctcccccactc

tcccgggggtggccgggaccccagccctcctcctgcccccgacccacctg

ggggccctctggacatctaccccgggagcctcgggcccaacagggaagag

ggctggaggacgctgttgagtccccccagtactcggcacctgtctaggtc

ccccaaaatgcctttgtcctggacctccctcctcggcccggggctccctt

cgagcctccgtctccccagtctgtacaatgggagggaggaagacttgtgc

gcccggcccacacgaaccatagagccgatctccgggctagaagtgagtgg

ggagcacttccaggtgacttagaagacggagacctcagaccaccgcctcc

ccctcaccagaggccacctcggggaccccccccggaggaaaaaaaatgcc

acctcttgcccgggggcgtctccctccagctggcgccggcgccagtccgg

gtctccacggcctcgccccaggcaattgggcccgttggcctgcgaaggcc

acgcccggggaggggtgccccctccccctttctggagccaccggccgggc

cacctccactgggtcaagcacagccctgagcggccgcgtgtccgaggccc

aggtgccctctagagccctgtagttcctgcccctctctgcccctctcggc

tcctgctgttccgccgctgtcgtccgaaccatcccaacccccagtccacc

cagacagcgcccgagctaggggagggaacggtctgggtaggtaactgcgc

tcggactgaccacgttcagcggtgaaggagcgtggcggggttagggtctc

ggggagaggccatccagagggtgtgcggcccgggctcctggggagggggc

agttggtggttagttactgctagggaggcccagagcatcgagggatcccg

**gagtgttcgcaagaggggctgcaggggtcgggccttggggtgagggtccc**

**ttagtggtggggcgcgttggcagcaggggccgccaggagcgcgcagggag**

**ggggcccgccggctgaggggggccggcccgcgggtcagtcccggagtcca**

Promoter 5

**gcggttcgggaattgcggacgcagccaatgggaggcggaggctgggaggc**

**gcgcggcgttgattggctggcttgggcttcttaggcgtgcgcggcccccg**

cttcatgtctgtgcaggagtcggcagctggcgccagggcggccggaggat

gccgaggggccggagccgggagggcccgaggccgaggcgcactctacccc

cagctcctaccctgtaagccccgccagcctccggacgtgctgtccctggg

cccgtcgccctcggggctcccgccggaactccttcactctcagaggccga

gtccctcccctccccacggctgcgtgtgtaagtttggggtttgagagccg

gctgggggcctggggcgctcctagctttgaggggactctggggggactta

gggcggggggccaggctgcgggcgactgctttggtgtgctttgttgagag

gtcctgattgtgccgcttagtactgcgctcagggggctgcgtgtgcatgt

tttcggggttgagatcagtatgtgtatctggcgccaagtgtgggtgtgtg

cgtgtcgctgggatcaagtgccacactgggtgcccgggcgcctgtctcca

acttttgagtctgtgttcatgtgtttgtctaccgggcgggggggctcagt

gtgagtgtctgactgaagtgctgctctgtgcgtttgttggggtcactggt

gtcgggacccgtccccgggcgtgaccccatgtgcacgggtgtgtgattct

ggagccgcgggtcaccacgtgagtgtgcgtggctgaaccccctcccccgc

cttcctttcgttttgagccttgggctttcctcccacccgggactggtgct

ctttctcgccggatctgggctggggctccgtggcgtgcgggacacctcgt

ggtgggactttggggggtgtcaggcgctggcggcacgcctcactccccct

tcctcgcgcagggccgttgccgtctgcacccagaccctgagccgccgccg

ccggcc**ATG**GAGGTGGCGCCCGAGCAGCCGCGCTGGATGGCGCACCCGGC

Exon 1

CGTGCTGAATGCGCAGCACCCCGACTCACACCACCCGGGCCTGGCGCACA

ACTACATGGAACCCGCGCAGCTGCTGCCTCCAGACGAGGTGGACGTCTTC

TTCAATCACCTCGACTCGCAGGGCAACCCCTACTATGCCAACCCCGCTCA

CGCGCGGGCGCGCGTCTCCTACAGCCCCGCGCACGGTGAGCACTGGGCCC

GTGGTGATGAGAACCCAGGCGCCGCGCGCCAGGCGAGGGAGGGGAGGAGG

GCCCGTCTGCTTGCTTCCCGGATGTAGGATCCGCAGGAATCGAGCTGCTG

AAAAATTGGGGCGGGAGAGGTGGGAGCAGCGGCCGATTGGGGAGGGTCTG

GGACCCACAGGGTTTCTGCCCACTTCCAGCTGGCCTGTGAGGGTTCCCTG

TAGGGTCTGTCCGGTGGGGTTCCTTCTATGCCACTTGTCCTTCAGCTTGG

ACTGACATTCCTGATTATTACCGGTGGGGTATTATGTTTCTGGCTTTCTT

TGGGGAGGGGATGACTGCTGGTTCTGGGAGTCGTGATCTCAATGTCTGTC

AGGGGCGTCCCTAGCTCTGCCTACCCTGATCTTTCTGCCCACCCTGATCC

TCTCTCTCTTTGCCCGCAGCCCGCCTGACCGGAGGCCAGATGTGCCGCCC

ACACTTGTTGCACAGCCCGGGTTTGCCCTGGCTGGACGGGGGCAAAGCAG

CCCTCTCTGCCGCTGCGGCCCACCACCACAACCCCTGGACCGTGAGCCCC

TTCTCCAAGACGCCACTGCACCCCTCAGCTGCTGGAGGCCCTGGAGGCCC

ACTCTCTGTGTACCCAGGGGCTGGGGGTGGGAGCGGGGGAGGCAGCGGGA

GCTCAGTGGCCTCCCTCACCCCTACAGCAGCCCACTCTGGCTCCCACCTT

TTCGGCTTCCCACCCACGCCACCCAAAGAAGTGTCTCCTGACCCTAGCAC

CACGGGGGCTGCGTCTCCAGCCTCATCTTCCGCGGGGGGTAGTGCAGCCC

GAGGAGAGGACAAGGACGGCGTCAAGTACCAGGTGTCACTGACGGAGAGC

Exon 2

ATGAAGATGGAAAGTGGCAGTCCCCTGCGCCCAGGCCTAGCTACTATGGG

CACCCAGCCTGCTACACACCACCCCATCCCCACCTACCCCTCCTATGTGC

CGGCGGCTGCCCACGACTACAGCAGCGGACTCTTCCACCCCGGAGGCTTC

CTGGGGGGACCGGCCTCCAGCTTCACCCCTAAGCAGCGCAGCAAGGCTCG

TTCCTGTTCAGGTAAAGGCAGGTGCTGGGGACTTCGTGGAAGAGGGGAGC

ATTTGCGTTTTTGTGGTGGGGAGCTGTGACTTGGGAGAGGTGGCAGTGTT

GGTTTCCCAGATCTGGGGATGGGTGATGTCTCGCTCTAATAGCCCCCAGC

AGATGTTTGAGACCCCCGGGTCAGGGAGAGGAGACTCTAAAACTTTTGCC

ATTTTCTTAAGTGCTTCCAGTGTACCCCCAAAGTTCAGTTCCTTGCCCCA

Promoter predictions (within 10kb upstream of TSS):

Promoter region predicted on forward strand (promoter 1)

Promoter Score: 54.76 (Promoter Cutoff = 53.000000)

Promoter region predicted on forward strand (promoter 2)

Promoter Score: 59.93 (Promoter Cutoff = 53.000000)

Promoter region predicted on forward strand (promoter 3)

Promoter Score: 90.59 (Promoter Cutoff = 53.000000

Promoter region predicted on forward strand (promoter 4)

Promoter Score: 70.21 (Promoter Cutoff = 53.000000)

Promoter region predicted on forward strand (promoter 5)

Promoter Score: 55.01 (Promoter Cutoff = 53.000000)

**Methylation analysis**

**Bisulfite modified & methylated**

agtgataaagtaaagtttttgtttttagagagttCGttttttggaggtag

**agatttttattttattttttttgtaCGggtatCGgtttCGCGgattttat**

**tCGagatttCGtCGtCGttaggtggCGttttgCGtttgCGtttgggtagt**

**agtCGggggtCGtCGggttggggtCGgggtCGgggtCGgggtaggggagg**

Promoter 1

**ggCGgtCGgggagtgggggCGgggttCGCGtCGtaggttgtgtgattttg**

**ggtttgatttgtttggagatgagttaatttCGtCGtataatagattttgg**

**ggagatagggaaaatggttgCGgCGttatttgCGtCGttatggggattat**

CGCGCGCGCGCGggCGgggCGCGagtttggaaaaatatagtttttgtCGg

CGgCGCGtgttattaaCGgCGCGgggattttaggCGgCGggttCGggttt

ttttgttttagttgtagaggttttggggttCGgaCGttCGggtgttttta

aatttCGCGgaCGgttttttttttttttatattCGtatatttCGttttgg

ttaagaaagtttttCGtgatatagtaCGttttatttCGtCGttttggCGt

ttgttatatCGgtatatattattttttgtCGtttttttagttCGgagttt

atttaCGttggtataCGgataggtttCGaCGattgttttttttCGtgttt

CpG island 1

tttttttagtttattagggtatatttgggtagagataCGgtaggatatat

atagttttaCGtagatttaagtatattttaagtaCGttCGgCGggagCGg

tgatatttgtatgtttatttCGgtaggtataggtagtttgtgtgtttCGa

aatataaCGtagggtgggtatttgtatgtatatagtatatttattttagt

ggttttCGgCGtgtttttttaaatagttatttttagggtagatatagtga

ggtattCGtaggatgtttattCGttgtattCGgataCGCGggtttgtatt

tttatttttttattaCGtgtttagttattttgggtCGtatgtttgtttat

aagtttagtgtagtatCGttttttgtgtgaattttttattCGttttatCG

gtCGttttCGgatatagtatagaCGtttgttatgCGtgtgtatggatggg

ttCGtatatttgtatttttttgtggtatatttgtttgatatattCGtata

gttgtatCGgCGttagtttatgtattggtatatttaCGtgtttatttaaa

taCGtCGggtatatCGgggCGtatatttagtggtttCGgtaggtaggCGa

gtggtaCGggtaCGgggagttCGCGtgtttttagtagagggttatttgtt

tgtttattagCGttCGgatttgCGgtggtaaatgatgtattCGatggCGg

gCGtatttgttgtgCGttttttgaaagggtttCGataatttggaaggtag

agataaggaatattatttatttCGCGgtattttggaaataatttttagtt

ttgtataattttattttCGggtagttttCGggagtCGggtagataaCGat

tggttatttattattttCGtCGggaataaagattagtCGgtCGagatgaa

aaattatttCGgatagCGgCGtagtttCGCGCGgagttttttgttCGtta

ttatCGttCGCGttttggtgtttCGttttgttCGCGgttCGtagtttatg

tttagtttattttCGttCGgCGttCGgtttttCGCGtttttttCGagttt

ggCGgttaagttttgtCGgtttttttattttagtttgtttattttatttt

gtttattttttttttgCGtttttatttttttttttggggttttttttaCG

**Bisulfite sequencing / MS-HRM assays GATA2 CpG island 1**

Primer set 1

GATA2F1: 5’-gttCGttttttggaggtagagatttt-3’ (TM=58.4C)

GATA2R1: 5’-CGAAATTAACTCATCTCCAAACAAATC-3’ (TM=59.3C)

Product size = 251 bp; 23 CpG sites

Alternative primer set 1

GATA2F1: 5’-gttCGttttttggaggtagagatttt-3’ (TM=58.4C)

GATA2R1: 5’- CCGACAAAAACTATATTTTTCCAAACT-3’ (TM=58.2C)

Product size = 369 bp; 38 CpG sites

Primer set 2

GATA2F2: 5’-CGggtttttttgttttagttgtagagG-3’ (TM=60.4C)

GATA2R2: 5’-CCGTATCTCTACCCAAATATACCCTAA-3’ (TM=58.1 C)

Product size = 297 bp; 21 CpG sites

Primer set 3

GATA2F3: 5’-GGAgCGgtgatatttgtatgtttattt-3’ (TM=58.8C)

GATA2R3: 5’-CGAATATATCAAACAAATATACCACAAAAA-3’ (TM=58.1C)

Product size = 403 bp; 20 CpG sites

Primer set 4

GATA2F4 5’-GggttCGtatatttgtatttttttgtgG-3’ (TM=60C)

GATA2R4 5’-ACCCTTTCAAAAAACTCACAACAAA-3’ (TM=59C)

Product size = 283 bp; 20 CpG sites

Primer set 5

GATA2F5 5’-TCGATAATTTGGAAGGTAGAGATAAGG -3’ (TM=58.3C)

GATA2R5 5’- ACCGACAAAACTTAACCTCCAAACT-3’ (TM=59.4C)

Product size = 339 bp; 30 CpG sites

**Bisulfite modified and methylated**

agttttagtaaagaCGtgttttaagttttttgagtaagtatttttgaggt

taggggCGCGCGtgtgCGtttCGgagagggtgggagCGgtttggagtgtg

gatgtatagggtgtgCGCGttttgagtgtttagCGgtttttggtCGgtta

ggCGgttggatttggtaggtCGggtgtagagtttCGggtatttttttgtt

CpG island 2

ttttgCGgCGttttCGttttCGtCGttattttttgttttttCGtaaagtg

atgtCGaaataaaaagtCGCGttgCGgtCGgtgggggagagagaggtagC

GtgagCGttaggaaggtagCGaggttagCGtCGtttCGggattCGttgtt

taagtttgtttattgtttgtCGttatatttattttagtagggtttCGtCG

tttattaggCGgataaaagCGgttCGttgaatattatgCGgtCGttCGgC

5’UTR

GtgtCGtttaggttttgttggtgagCGtCGttatttCGCGtttaggtttC

**GCGagttCGtttgtCGCGtatttCGttttgtttttagttttattttaggt**

**ttCGttCGttCGggggCGtCGtttatCGCGtttCGttCGgtgagtttttt**

**ttatttttattttttttgggttCGgtttttttCGttttCGgttCGgttCG**

Promoter 2

**tttggtattCGaatCGtttggttCGtttgttttgtggtttttatttttgg**

**ggttCGttttggttttgttagagatCGgaaattttggttatagggatttt**

**aattagagggagtatttggttatttgggtatagtaggttCGtttCGggtt**

ttttgtttttttatttttCGgagttataggttaCGagattttggtatttt

tagagtttCGtaaagtattttCGtttttCGgttttCGaattggggttttt

gtttCGgtCGtttttaatttttaggttatCGCGgCGgatgCGttCGagtC

GggtCGtCGagCGgCGgttgtatttgtCGgttggtttCGtgCGtCGgttt

**ttCGCGgtttaatttagttCGtttttgtttgCGttttCGCGCGttgCGtt**

**tCGtagttttttattttgtgtgattggggtgCGtgtgggggagagCGtCG**

**ggtttggaagttttttttCGttttaagtCGgagtCGgaattCGtttagtg**

Promoter 3

**ggattttaataagaatggggtCGtCGCGggtttgaagatttgtCGtCGgt**

**aggCGtagaCGgCGttttggatttgtttgaaaaCGgggtatttaaaatta**

**ttttgtggCGgttaggtttgaatttCGCGttgttttaattaCGaggggaa**

aggtttCGCGtttagggatttCGtgtgtggCGaaCGtttttttatCGgaa

aataaggaaggaaattCGttgttttggaaaaagttttttttttCGaggtC

GggaatatttggtggtgagttttaggtttaCGtaggtatttCGtgttagg

attCGtttatgagtaggtaggattCGagaattaggtagggttCGtgCGgt

agtCGaggCGtttggggagCGttttttagtCGggttgataaattgtagat

aaattgtgtttaaCGaaaCGgatttattagttgttaggtCGtCGgtttCG

gtCGttttaaataaatCGtggttgttCGtggaggagggagaagtaCGgtt

CGattgttttCGggttttagtagggtttCGCGgggagtttgttaggtttg

aaggtaggggttagaagCGatatagaatttCGgaggCGtttgggtttagg

gtgtCGagatatttaggaCGtgggggttatagattttaCGatttttaaag

atatagaatagtaatgaggtgggagagtttgtattgatgtCGagggaggg

agttttttgttttaaggggttggaattgaattttaaggagtaggaggggt

CGttatagtagtagttttttttttgggaattttttCGgagggatggttgt

tggtttgagatttaatgtttCGgttttaagggaatttttgaatttatttg

ttaaggtattttatttttttttCGtattttttaaggtttattgttagtgt

ggggttgggaggtCGttgggttgCGaattaaatttttttatggaaggtag

ttttttagtaaatgggtttttttgatattttatttttagttttatatCGC

GggttaattagttgtttataaggaaaaggCGagaagaattaggttataaa

gggagggtaaatttatggttttaaaggggtCGtttCGgatgagttaattt

taaataaagggtttagaggggggggggggttggaCGgtCGgggagatttg

ggtatttttgtgtttttatttggtattCGCGgtttagtagaggtttgaga

agtattttagaatCGggtattagatttgttatttttttagtttttaggta

gaagtatttaggttaaatggtggCGatCGtCGttgtgagttttCGggtta

aaagggtttttCGaaaattttgttttttggttgattttttttagttttta

gagaaattttgtttttaagtaaggaaagtgatagttttttaatgtgatta

aaggtagCGttagtatttttaattatattttCGaaCGaataaagtattga

aaaagggaaCGCGtttttttaaaggtgttttggggattttaaagttttag

tttataaattggagtaaatttgtttttattaggtttggaatagCGtttta

agattttagtagattttgggggttgCGttgatttttttCGggagtttgtt

tggggtttaaggtgggaggattatgttttCGgtttaatggggaggggttC

GgttggtgtttttCGgtttgtttggtatatatagatattgtCGagCGCGg

gtttttttttattggttagttgggCGttttgttatttggCGtCGtatttt

ttttttttaggCGggttCGttttCGttagagaatgtagtagtttCGtatt

ttaCGtaggatttgtaatCGaggtggaggtttCGgttaagtCGgtttttg

tttgCGttgtCGaggaaggtaattttaaggtttgaaaggatttggtagaa

tttttgtttttttttttttttttatatCGgattgCGggtaaggaggttgg

ttCGggtttttCGaggtttttgtttaagtattttttaatCGttttgttaa

gtttttttgtgCGgCGattttttgagttgtCGagCGgggtaattaaattt

tttttttCGtttCGttttgCGttaggtaggCGgtagtatagggttgaCGt

ttgggtaggggatttagttaggttggttatttttattatCGtagtggtCG

ggattttgtCGCGgaggggttagaCGtCGagttCGttgCGttgaaattgg

gaatatataCGgaaCGgggagggggagggtaatttttatCGCGtCGgtgg

gagaaaaaggCGaattatttgttttttCGagggaCGCGCGtagttatttt

tttgagagtCGCGgtatCGatCGCGgtCGggCGggaagttttCGttCGgt

tttggCGtttatagtttagCGgttagttgttggtttgtttttCGCGttgg

gttttagggttCGCGttCGagCGgggtagttttgtCGgataCGggggatt

atttttggggaCGCGgtgttttttagagggttttgttagttttCGgagaC

GttaaataggttCGagtttCGCGgCGattttattttaCGagttgatgaag

aatCGtaagttaaaggatgggaaaagttgagagatagaCGgaCGgagaga

tagtgggtttCGgCGggatCGtaCGCGttgaggggaaCGttaatCGggag

gtaCGgagattgtttatttgttCGgtttggtCGCGgaggttCGgCGttaa

gggtttCGCGttCGgtttttCGttttttgCGCGgtttttCGggttggCGt

CGgttttCGttttCGtagagtggagtttCGagtagatCGggtttCGCGCG

ttttagCGtggaggggagCGggaggtttagtaggCGgttCGggtaggCGg

**gttttttaagggtaCGagaCGCGttggtttttagtttaatggagttttgC**

**GttttttagtttCGCGttttatttgCGttgaggtttCGgatagataaaCG**

Promoter 4

**gaCGttagaCGtttaggtaggaggaggttttagtttgagttCGCGgtttt**

**ttggCGttgttttgaattggtttgggagggggtgaCGggggCGCGttCGC**

**GgagttgggtttagtCGggCGttttCGgagtCGaggggatCGagggtttt**

**ttttttttttCGgattattaaaaagtttattttttggCGaatCGggtgaC**

GttaggggttCGgCGtCGCGgtggCGgggtCGttCGgtCGgagaagtCGt

ttttagttatttaattatCGattgttaatttCGtCGttttttttttattt

tttCGggggtggtCGggattttagttttttttttgttttCGatttatttg

ggggttttttggatatttatttCGggagtttCGggtttaatagggaagag

ggttggaggaCGttgttgagtttttttagtattCGgtatttgtttaggtt

ttttaaaatgtttttgttttggattttttttttCGgttCGgggttttttt

CGagttttCGtttttttagtttgtataatgggagggaggaagatttgtgC

GttCGgtttataCGaattatagagtCGattttCGggttagaagtgagtgg

ggagtatttttaggtgatttagaagaCGgagattttagattatCGttttt

tttttattagaggttatttCGgggatttttttCGgaggaaaaaaaatgtt

attttttgttCGggggCGtttttttttagttggCGtCGgCGttagttCGg

gtttttaCGgtttCGttttaggtaattgggttCGttggtttgCGaaggtt

aCGttCGgggaggggtgttttttttttttttttggagttatCGgtCGggt

tatttttattgggttaagtatagttttgagCGgtCGCGtgttCGaggttt

aggtgttttttagagttttgtagtttttgtttttttttgtttttttCGgt

ttttgttgtttCGtCGttgtCGttCGaattattttaatttttagtttatt

tagatagCGttCGagttaggggagggaaCGgtttgggtaggtaattgCGt

tCGgattgattaCGtttagCGgtgaaggagCGtggCGgggttagggtttC

GgggagaggttatttagagggtgtgCGgttCGggtttttggggagggggt

agttggtggttagttattgttagggaggtttagagtatCGagggatttCG

**gagtgttCGtaagaggggttgtaggggtCGggttttggggtgagggtttt**

**ttagtggtggggCGCGttggtagtaggggtCGttaggagCGCGtagggag**

**ggggttCGtCGgttgaggggggtCGgttCGCGggttagtttCGgagttta**

Promoter 5

**gCGgttCGggaattgCGgaCGtagttaatgggaggCGgaggttgggaggC**

**GCGCGgCGttgattggttggtttgggttttttaggCGtgCGCGgttttCG**

**ttttatgtttgtgtaggagtCGgtagttggCGttagggCGgtCGgaggat**

gtCGaggggtCGgagtCGggagggttCGaggtCGaggCGtattttatttt

tagtttttattttgtaagtttCGttagttttCGgaCGtgttgtttttggg

ttCGtCGttttCGgggttttCGtCGgaatttttttatttttagaggtCGa

gttttttttttttttaCGgttgCGtgtgtaagtttggggtttgagagtCG

gttgggggtttggggCGtttttagttttgaggggattttggggggattta

gggCGgggggttaggttgCGggCGattgttttggtgtgttttgttgagag

gttttgattgtgtCGtttagtattgCGtttagggggttgCGtgtgtatgt

tttCGgggttgagattagtatgtgtatttggCGttaagtgtgggtgtgtg

CGtgtCGttgggattaagtgttatattgggtgttCGggCGtttgttttta

atttttgagtttgtgtttatgtgtttgtttatCGggCGggggggtttagt

gtgagtgtttgattgaagtgttgttttgtgCGtttgttggggttattggt

gtCGggattCGttttCGggCGtgattttatgtgtaCGggtgtgtgatttt

ggagtCGCGggttattaCGtgagtgtgCGtggttgaattttttttttCGt

ttttttttCGttttgagttttgggttttttttttattCGggattggtgtt

ttttttCGtCGgatttgggttggggtttCGtggCGtgCGggatatttCGt

ggtgggattttggggggtgttaggCGttggCGgtaCGttttatttttttt

ttttCGCGtagggtCGttgtCGtttgtatttagattttgagtCGtCGtCG

tCGgtt**atg**gaggtggCGttCGagtagtCGCGttggatggCGtattCGgt

Exon 1

CGtgttgaatgCGtagtatttCGatttatattattCGggtttggCGtata

attatatggaattCGCGtagttgttgtttttagaCGaggtggaCGttttt

tttaattatttCGattCGtagggtaatttttattatgttaatttCGttta

CGCGCGggCGCGCGttttttatagtttCGCGtaCGgtgagtattgggttC

GtggtgatgagaatttaggCGtCGCGCGttaggCGagggaggggaggagg

gttCGtttgtttgtttttCGgatgtaggattCGtaggaatCGagttgttg

aaaaattggggCGggagaggtgggagtagCGgtCGattggggagggtttg

ggatttatagggtttttgtttatttttagttggtttgtgagggttttttg

**Bisulfite sequencing / MS-HRM assays GATA2 CpG island 2**

Primer set 6

GATA2F6: 5’- CGgtgggggagagagagG-3’ (TM=59.1C)

GATA2R6: 5’-CGAACCCCAAAAATAAAAACCAC-3’ (TM=59.5C)

Product size = 428 bp; 45 CpG sites

Primer set 7

GATA2F7: 5’- gatCGgaaattttggttatagggA-3’ (TM=58.4C)

GATA2R7: 5’- CGACCCCATTCTTATTAAAATCCC-3’ (TM=59.8C)

Product size = 451 bp; 40 CpG sites

Primer set 8

GATA2F8: 5’- CGtttagtgggattttaataagaatgg-3’ (TM=58.5C)

GATA2R8: 5’- ACCTAAAACTCACCACCAAATATTCC-3’ (TM=58.7C)

Product size = 278 bp; 21 CpG sites

Primer set 9

GATA2F9: 5’- CGttggtttttagtttaatggagtttT-3’ (TM=58.3C)

GATA2R9: 5’- GTCCTCCAACCCTCTTCCCT-3’ (TM=58.1C)

Product size = 490 bp; 42 CpG sites

Primer set 10

GATA2F10: 5’- GggggtagttggtggttagttattG-3’ (TM=59.8C)

GATA2R10: 5’- AACTACCGACTCCTACACAAACATAAAA-3’ (TM=58.7C)

Product size = 334 bp; 26 CpG sites

**GATA3; 10p14 (+); 20.5 kb in size; 6 exons (5 coding)**

Two transcript variants: transcript variant 1 (NM_001002295.1)

Transcript variant 2 ([NM_002051.2](http://www.ncbi.nlm.nih.gov/entrez/query.fcgi?cmd=Search&db=Nucleotide&term=NM_002051&doptcmdl=GenBank&tool=genome.ucsc.edu))

Two CpG islands:

First CpG island is 6,955 bp, contains 509 CpG sites

a second one in the first exon

tggtgcaggggccggagtcctgggtgctatagcggtgtggctgagcggcc

ccctgggacggaggagaggtcgggtctagaagtagcaagcccctggctag

gagtcactgatgccactgggggcagggtaagatgtccaaagcccgggaga

cccagggacgccggctgggctgcctccaccccttcttttcctcccgtggc

acccacctgtccccccacctgccaaggccaggagggagggagagagctga

aaagggcacttttcctccagggtgactccaattcctttccactcaccctc

**aaggcgagttccttctctcccacccaacccggggctccccccaactgcca**

**ctcgcctcacgtgtgagttgttaaacccgaaagcagaaatcccctgccct**

**ctcccttcctgtctccccctttccgctgcacccctacctacccaattttt**

**Promoter 1**

**atctcgctacaatcaggggttttctcaaccggggaaaaggtggtagtggc**

**ggtgggagggtcaggatccctcgaagtggaggccggggccgggctggggg**

**cggagtgcgggggcggggggaatctcccagcgcatctcctcttttttgac**

tctccccacggctcgcggctctcctggcctgggcgggtttccttccgagt

tttaaacggtgatcgatgaataagcaaaacaaacaaacaaacagaaaaaa

aaagaaaaaagaaaaagaaaaaagaactaaaggaaggaaaagaaaaaagg

gtaaatcaaatcacataccatccccaaaacgatagctcatttcacgggga

aggtagactcctggcccggggcgcgtgggcgtgggagccgcgaggagagt

tgccccttggacgcacccgcctccatcatcttcccattgccagtgagttt

tagggtccctctttctctgctttcccccaagttccctgctctctgggttt

tgtttgaagagagccagccttgctgggccgggatcctcaaccagtattgc

ccactgcccgactgccttttcttcccgtttaggtcttgctccccgctctc

cgcatttcagctcgcaagggaaagaaaacgggggcccgagtggcagagac

**CpG island 1**

agagataatatgtatttaacctgcgcggtccggggcgccctgccgcagcg

ggctccccgcggtgtggcccgcgcagccggggccagcggcggcgcctgga

gccgtgtccccggcataaccctcccgcccggcagccggcgagctgtttat

acagctttacctgtgctatagtcctatccagtagaaacccttgcgcggcc

agggtaacctaatccaggggccgaggcggaggagggggcggtgaggctgg

ctgggttggagacggaggtgtgcgcgctccaatacccgggatctctcctt

tgcccctcctcccaccatagggaaggaaatatggacatggttcgggtgac

agaaatggggacccggggctaaggaggaaggaggccagttttcacggccc

ggtcctccggcttctctcctttgaacaggcgctggggacctccgcggcgg

ccacagctggacgcgcgccgcgaaaggcgcggcggtcccgcgggcgcccg

gcgtgggctagagaggccggccctggcctggctcaggtcgccctcgcccc

gctttcatcttgccccagtcgccctttacaattcccacaccgaaggcaaa

gcaagcaaggaaagaagccggcgcctccccaagcggaccctcccttccgc

gcagcctcggccactcttccagagcgccccggtgcgtactgcggcgggcg

cagggaatcgaattaaacgtctctgggcggttattgtctttctctaattg

ctccaacaatggcctctattaaaatggagacacccaattaaaggccccgc

cgggccgctctcggctgccggcaaagctctggcccccggcctgcgacgtg

**cagaccgcggggccgcggacccggctggtgcggggtactggcgccggcct**

**cgacacctttggcctccacttcctccccagactcacagcgacagcggccg**

**Promoter 2**

**cttcacaccgtttttattgaccgatcgcagcccagcaagattgatcgagc**

**tggaatgggaagggacttctcctcccccaggcccagctcgccagggcctc**

**gggccgtgctgcagtttctggcctttggtgtcgctccccgccccccagcc**

**ccgcagattccc**ggcttcttttctgtctgcgcggccgggaccgcccaggc

aggcgccggggctccggggctccggggggagggactcggcggctcggctc

ggctccgcttctttcttctgcctgcaaatatttgctgcctcgctggaaat

ccgacgatttcgcgcgcgctctgcttgcaaagtctttaagtaaacacgct

caaatgaccgccccgggcggcccgaggcacgctctctccccctccgcggg

attagtaactttaggacttcgaccccggggctccgctttgcctgttaccc

aggtcgggcagcgcgcgggcgcccggggccgcttctcccggcacctcggc

cccgcggagctcgcctggaagcgccggttgcctggctctggtgggtggcc

cggccgcgagcatctccgcgccctgggtctgtagccctagggctgagccc

cagaaagccgcgcctccgctctgcgccatggcctctgctttctgtacccg

actgggtttccccacttccactgggacagggtctcttcgcccctgggagg

gcgggtgggagggacttgcacgtgggcatgtgtttgggggtgtgtgtggt

agcttaacccctaccgagtggagaaagtccggctccatttctcacctttt

tcccaagaggacacacataccccgctcaacttgacgcctaaagcagccgc

ttccaggcctggggtttcggataggccaagccggctgctgccgacccgca

cgccgccccgggccccggctcctctctaggcggtggccgcgccgtcgcct

gctcggctgcgggatgcttgctcccaaggtcccaggcgtccaggttgatc

gccagccccttaccctggtgagggaggccttgcgccctgcagaagccccg

ggccctgcggcggcggcggcggcatcctcctggtcgcctcctcgctttcc

tccacccgagctccgccggctgctccttgggaggaaggggactcgctaga

ccaggccgaggcccaggtacctgggagggggtcgcgtcaggtctgcggga

aggcgctcaggcttctccgcaggaggccaggatcagctttgtggcggtgg

**cacagggagaatcaggaccacctttcccccttacccccggccaggatccc**

**Promoter 3**

**cctttccctcccccgcccgccaagggaggaggaagcccggcctgctcttg**

**ccggcgaggcgggtcaggccggcgcctggcctggcccggacccccgcagc**

**ccggctctccgcctcaggccacgcgccgcagcctctccagcctcgctccc**

**ttcccccttccttccccgtgggtcccgaggccgacctcggccctccctcc**

tcggcatccccctcccatccttttcccttccccgctgccctgtgctccgg

gctccggggctgggcccgtgacgtcaaacccagtgtggcgtcggcgagac

tggccggcgcgggccatcaaaagagcaacgtcctctctcccaattaccca

ctgtcagtccgggaactggggcgggccggctgggaattaaatgctaaata

ccccctaccggctggctccattacccgggacatccagccccaggcccccc

acccctctctgccagcctcaaaccacccagaccaagaagggccttcgaga

tcttttatttttctaaaggtgggggttgcccttctccatccccggccagt

ccgacttggtgctcgcgattgaatttaaacgaataatccctacttcccca

tccaaaattagcggataggcgcccttgcaccggaaggcctcctcaccagt

cttttctttttgtctttgaaataattcttagcctcagaagtcagcatttt

caagacctggcatccgcagcgtttctgacgcggggacttctctcttcggc

tctgaggtctccgcgcgcagaacccatggcagcttttggtcttacgcgtg

gctgtgagctgggctcacgctgcgtggcgcatccacccctcgattgacgc

gcggagaagcatttttcattttttctttcagcttgtatccttaaatctta

atcccgggcttcttagcaaaaatgcgtgtgcattcgtttctccttgttta

tggagaggtttccggtgaatgttagacagcaa**ctaaataattaaaaggtt**

**tagagagcgctgtgagcaggagaagatgcgggcagcctggctggcccagg**

**Promoter 4**

**agagacgagtggtcagagaatgaaaggaaaatcgatgcctcttaactggg**

**ccgcctaataacgggagaccaagtgggctcaggagaaacgtggcgggggg**

**ggggggagctttctttaaaaaattggggttctttctttctgtcctatata**

**tggaaattcagccctgatttcaaacaaacatcagatttaagaaggtgagg**

actccaaagaagatccaaacgcgagaccaggaagattcacgtttccacta

cttcttccaatacatttcactaatctttctacagggatagtgttttaaaa

cacgacgaccctcacacaccaaggtgaagttgaatattgttgttgttcct

tcaccgcatgaaaggaatccttcagaacttactttcagggacggcttctt

ctatctcgaatttttgcatttgtagggaattttatggggtagaggagcga

agagggaacaatgtctccaattcaaataaaaccccaaattaagctctcag

ccccatcggtgggatagcctgcgggggaacccact**ccctctcctggcagc**

**attgccctgggggcgagcagagagtggatttggagtcttcttttcccgcc**

**Promoter 5**

**tgacccctgctcgcccgagcagcattttcgggccagcggctgcagccacc**

**cgggcgccgctaggggatgcagtggctcaaaggaccgagcgggcggtgca**

**ggttggaacccgcggggcggaccaatcgcggctcggccacagcctcgccc**

**gctgattggtccctccaggccccgcccccgctcgccccgcccctctcgct**

ggggcgcctcggagccgcgtgccctccgccccggggtgcccattgcgcag

agcgtggcctggagacccgcgagccggggaaggtcgccgtggagtcccga

ccagaggccggggttggggtcggtgcagaccgagggctggtttccttgac

tgtgggagaaacgccgggagccggagtaagtagggctccgggcggggcga

aaggaaaagttgggtccctagagtgaagaccgagttctttctgtccgtct

acactgagcgtactcggggaatgagttagagccagtctcttcctcccctc

cccccttctcatccctcactgttgccactcaagtcaaaagcacacattga

ttacaaatattaggtctggaaagggcagctgcaacagctgaagcgtgttc

actctgggggcttgagagcgcagaaggctcgggaaagaggtgacaatgac

aacaaaattgacgcggacgctccagtcaaaggcatctcccctttatccga

tgactcaccctcttaggaagtcggcccgagaggcaaatctcaaaatacct

tgacatgaaacattttgtttttctgatcaatttaacgcgcacgtttcccc

acatcgatgcgctctcccaaacaccctgcattagatcctaataatgatcc

atgcgtgcctattttttaaaagtctgaaaaagaaa**attctgcccatcgaa**

**atgaacttcatgaatggggcaggctggctgcaccgggacggaatcgtcca**

**Promoter 6**

**cccgacccgaatgaattggcaggagccgcggccacatttaaagggccaga**

**gcgcgcgttccctcccgtccgcccccaagccccgcgggcctcgcccaccc**

**tgcccgccgcccctccgccggcggccgccctctgcggcgcccctttccgg**

**tcagtggaggggcgggagg**aggggcgggggtgcgcggggcggggggagaa

gtcctggagcgggtttgggttgcagtttccttgtgccggggatcctgtcc

cctactcgccagcgccaggctcctcccccccggcgcggatgacactagaa

cctccttaagttgcgtcgcgccacagctgtctgcgaacactgagctgcct

ggcgccgtcttgatactttcagaaagaatgcattccctgtaaaaaaaaaa

**5’ UTR (Noncoding exon)**

aaaaaatactgagagagggagagagagagagaagaagagagagagacgga

gggagagcgagacagagcgagcaacgcaatctgaccgagcaggtcgtacg

ccgccgcctcctcctcctctctgctcttcgctacccaggttggtactggt

gactttttttttttttaagtttgattttttgcccccaaccacttgggagg

acctaaatcaattttaaaaactcaactctcctcttttggaggttttctag

gggctgagaggacggtcccgggaccggtgtccccgagggagggacttgcc

ctccaagtcgtaacagtcagccctgggacttgccctccaagttgctcagc

cagccccggctcccgcgagccgggctgcagggacgtccccgagagccctg

cgggctccgcggccgtgtccccgcgctcccgtgcgggtctcgggtgcgct

gggcgggcgggcggcgcgaggggaggttgtgccactccagcaactcaggg

gctcatccaggtctcccattctctcccttgcaggtgacccgaggagggac

tccgcctccgagcggctgaggaccccggtgcagaggagcctggctcgcag

aattgcagagtcgtcgcccctttttacaacctggtcccgttttattctgc

cgtacccagtttttggatttttgtcttccccttcttctctttgctaaacg

acccctccaagataatttttaaaaaaccttctcctttgctcacctttgct

tcccagccttcccatccccccaccgaaagcaaatcattcaacgacccccg

accctccgacggcaggagccccccgacctcccaggcggaccgccctccct

ccccgcgcgcgggttccgggcccggcgagagggcgcgagcacagccgagg

**First coding exon**

ccATGGAGGTGACGGCGGACCAGCCGCGCTGGG**TGAGCCACCACCACCCC**

**GCCGTGCTCAACGGGCAGCACCCGGACACGCACCACCCGGGCCTCAGCCA**

**CTCCTACATGGACGCGGCGCAGTACCCGCTGCCGGAGGAGGTGGATGTGC**

**Promoter 7**

**TTTTTAACATCGACGGTCAAGGCAACCACGTCCCGCCCTACTACGGAAAC**

**TCGGTCAGGGCCACGGTGCAGAGGTACCCTCCGACCCACCACGGTGAGTG**

**CGCCCGGGGTGCCGGGGCTCCCGCCGGCCG**CTTCAGCCGTCCCGGCTCGG

GGAGGTCGGGAGGGACCTGAGGGCGGGGAGAGGTCAAGCGAAAGCCCCCA

TCTGCCGTTCCTGGTTCATTTACAAAAAAATTGGGGCCCGGAAATGGGCG

AGGAAGGCCTCTGGCTCGTCGCGAGAGTGTGTTTTGAAAGAGTCGCAGCA

GGCGCCTCTCCCGGCTGGTGGCCCTGGGGCCTGGCGCTCACCTGGCGGGC

GCCAGGCCGCAGCCCTCCGTCTCTGCGCGGCTGCAGGTTTTGGGGTGGGG

GGTCTCGGGATGTCCCCAAGCGCGGGGTGCCCTGGCTCTGCCTCCGCGGG

TCCGGGCTCTCTGGGCCCGGTAGCCGGCGCCGGACAAGCACGGCTGGAAC

CCGGCTCTTCCAAAAAACCTGGCGTTCCCTGTTACCCGCTAGCTCTTTCT

AGGCGGGTGGGCGGGGTGGAGGGGGCCCTCTGCCAGCGTCCCTCAATTCG

CACATTTTCAGAAAGGCCCCAGAGACCTATTTATTCACACCCTTCTCCTT

GACCTTTTCCCAGAGCTAGTGCCTTTGGTTTTTAGACAGGTCTCTTACCT

CCTGGCTTCAGGAATGGAATTCTGATGCTGAAGGGGTTTGGGGGGGAAGA

CCCCTGTCTTAAGTTTGAGGGATCTGAGATTTCCCAGATTCCCGGCTGCA

CAGAATTTTCTTGCGTCTTTGCTTTCAAGTCGCCTCCTTGCCTGCAACTC

TTGCCTTACTCTGTCTCTGGGTACTGCCCTCCATTAGCCTCCCACCTAAG

ATGTAGGATACACCCCCGTTTAAATAAAGGCAATTCCAGTACCACCTCTT

TCTCCCTTTCACCTGGAGAAGTTCAGGAGAGTTCTGAAATGTAAAAAAAG

AAGACCAGGCCTGTGTAGTTTGAGGAAAAAAGATCGAACACTTTCCAGCT

CTAAGTTTGTTCCTAAAGAAGAAACAGGGGTAAAACATCGGGCAGAAAAA

GTGTGGGGCTTTCTGAGTCCAGCCAGACCGAATTCTGCCCTGATTCCCCT

ACTCAGAGCCTGCCTTGACACGGATGACATAGCCCCTCCGGCAGCAGGCG

TCCTCTACCCTGCTGTCGCCAGGTTTTAAACAATCGTTTCTGCGGATGGG

GCGCTCTTTGCCCACGTTCTGTGCGGATCAGCAGTTAGGTGGAAATGCGG

TAGAGGCAGACTTAATATTATTTACTATACTACTTCCTGCATAATATGAA

TCTTGACCCTTGGTGTTCAGAGAACTCTCTTTCCCTCTCCACTCCCCTCC

CCTTCCTCTCCCTTAAGATTTTAGTTTCAGTATTGTTCTTAATGGTTGAA

TCGAATGTCAAATGCTGAGGCAGGTACTGGCTTTAAAGATCAAGAAGTGT

GTGGATTTGCACTTGCTTTTTAAATGTACCCCTCAGAGTGTATGAGTTAC

AGCTACCTTAAGATTGTGCGTTTTAAATGAAGGCTAATCAGACTCTGTGT

CTGAGTCATCTTTCATTTAAAATATACACATCAACTGGAATTTTGTGTCT

TAGGGGCTCAGGTGAAAATTCACCCATGGCACTGGTGGGATCATAGCTTT

ATTGAGGGTGCATGGGGCTTACGTTATCTCCTTCTCCTTTAGGATTTGGG

AGGGTGAGAAAAGCAGAGAAAATGGCCATCCCAGGGTCCAGCCTTGGGAA

CTTTCTCCAACAGCCCGAGCAATGAAAACGTCCCTGCAAATCCCATTTTA

GGCCTTTTGCGGGCAGCCTGGCCGTTTCTGAGCAAGCACTGGGTTAGGTT

TCCGGAAACTAACCCTGAAAGTCTCCTGACTTCTGTCCCAAGGCCTCTCC

TTCCTGTATTTGGTGCTGGGAGCTGGGCCTGACTCCAGGGTCGTTTTCTG

GTGTGGAGCAGTTGTGTGGCCGGGCTCCATCCTTCAGGCCTCCTCACTCA

CAGCCGGCTCTCTTATCAGGCTGGCTGGGATTAAGTCCGAGTCAGGGAAC

TCAGGGCCATTGAAGGAAACCCATAAATCAAGTCAGGTTCTAGAGAGAGA

CCTTGGGGAATGTGATTTAGGCGGTGTATCTGGAGAGGCCAAATAAGCAA

AGGGGCCCTTCTCCCCCTCTGCAGCAACCTCTCGGGTGTCAGCCACAGGC

CCTTCATTCTGCTACATTTGATGGGACATCCCTGTGGGAGAGATGGGTGA

AGGATTCTGTCCCCAGCCTGACCCCCAGGTGTGCCAGGCAGGTACTCCGG

GGACCGCCAGGATGAGAGAGTGGGCCTGAGCCCGGGCTTTTGCTGAAAAG

GAGGCCGATGCGAGGTAGAGATTCCCCAGGTGTCCCTGACGGCCTCCCAG

GGCCACACTCACCCTCCTTCTCTCTCCTGCCCTTTCCCCGTTGCCCCACA

GGGAGCCAGGTGTGCCGCCCGCCTCTGCTTCATGGATCCCTACCCTGGCT

GGACGGCGGCAAAGCCCTGGGCAGCCACCACACCGCCTCCCCCTGGAATC

**second coding exon**

TCAGCCCCTTCTCCAAGACGTCCATCCACCACGGCTCCCCGGGGCCCCTC

TCCGTCTACCCCCCGGCCTCGTCCTCCTCCTTGTCGGGGGGCCACGCCAG

CCCGCACCTCTTCACCTTCCCGCCCACCCCGCCGAAGGACGTCTCCCCGG

ACCCATCGCTGTCCACCCCAGGCTCGGCCGGCTCGGCCCGGCAGGACGAG

**CpG island 2**

AAAGAGTGCCTCAAGTACCAGGTGCCCCTGCCCGACAGCATGAAGCTGGA

GTCGTCCCACTCCCGTGGCAGCATGACCGCCCTGGGTGGAGCCTCCTCGT

CGACCCACCACCCCATCACCACCTACCCGCCCTACGTGCCCGAGTACAGC

TCCGGACTCTTCCCCCCCAGCAGCCTGCTGGGCGGCTCCCCCACCGGCTT

CGGATGCAAGTCCAGGCCCAAGGCCCGGTCCAGCACAGGTAGGAGCCAGC

TCTTCCCTGGAGCCTTTTCTCCTCCCTCCTCCCCTTTTCCTCAATCCAGG

GCCGCACCCAGAGGGACCCCTCAGGGGAGCCGGGGTGTCCCAAAGCCTGC

TGAGATGCCATTCTTCCCATTCTTCCTGCCGGGAAGGCACTGCATGTCCT

CCCATCCTAGCTCACGCCTGGCCTCGGAAGCAGAGGGGAAGTGGGATCTG

ATTTAAAACCCCCCAATGAGCTGGGATAGGAAAAAAGACAAACAAAAACA

AAGTCATGCCCTCTTCTGCCTGTCGCAGGACTTCTGGATTGGGCTGGTAA

CCTTTAGTCAGTTTCCAAGACCAAATGGAAGGCCGAGGGAATAATTCTTG

AGTGTTTCATGCTAAAACGAAACCTCCTCAAGCCTAAACAAACACAGGTC

CCTCTATGACCCCTTTGGCCTCTGTCATTCTGACTGTTCCTCTCGCTCAG

CTCAAAGGGGACCACGCAGTGAGGACAGTCCTTTATTAGACGTGTAGACA

Promoter predictions (within 10kb upstream of TSS):

Promoter region predicted on forward strand (promoter 1)

Promoter Score: 201.50 (Promoter Cutoff = 53.000000)

Promoter region predicted on forward strand (promoter 2)

Promoter Score: 53.06 (Promoter Cutoff = 53.000000)

Promoter region predicted on forward strand (promoter 3)

Promoter Score: 53.65 (Promoter Cutoff = 53.000000)

Promoter region predicted on forward strand (promoter 4)

Promoter Score: 55.86 (Promoter Cutoff = 53.000000)

Promoter region predicted on forward strand (promoter 5)

Promoter Score: 56.48 (Promoter Cutoff = 53.000000)

Promoter region predicted on forward strand (promoter 6)

Promoter Score: 57.80 (Promoter Cutoff = 53.000000)

Promoter region predicted on forward strand (promoter 7)

Promoter Score: 55.99 (Promoter Cutoff = 53.000000)

**Bisulfite modified and methylated:**

atttatttgtttttttatttgttaaggttaggagggagggagagagttga

aaagggtattttttttttagggtgattttaatttttttttatttattttt

**aaggCGagtttttttttttttatttaattCGgggttttttttaattgtta**

**ttCGttttaCGtgtgagttgttaaattCGaaagtagaaattttttgtttt**

**ttttttttttgttttttttttttCGttgtatttttatttatttaattttt**

**Promoter 1**

**atttCGttataattaggggtttttttaatCGgggaaaaggtggtagtggC**

**GgtgggagggttaggatttttCGaagtggaggtCGgggtCGggttggggg**

**CGgagtgCGggggCGgggggaattttttagCGtatttttttttttttgat**

tttttttaCGgttCGCGgtttttttggtttgggCGggttttttttCGagt

tttaaaCGgtgatCGatgaataagtaaaataaataaataaatagaaaaaa

aaagaaaaaagaaaaagaaaaaagaattaaaggaaggaaaagaaaaaagg

gtaaattaaattatatattatttttaaaaCGatagtttattttaCGggga

aggtagatttttggttCGgggCGCGtgggCGtgggagtCGCGaggagagt

tgttttttggaCGtattCGtttttattatttttttattgttagtgagttt

tagggtttttttttttttgtttttttttaagttttttgttttttgggttt

tgtttgaagagagttagttttgttgggtCGggatttttaattagtattgt

ttattgttCGattgtttttttttttCGtttaggttttgtttttCGttttt

CGtattttagttCGtaagggaaagaaaaCGggggttCGagtggtagagat

**CpG island 1**

agagataatatgtatttaatttgCGCGgttCGgggCGttttgtCGtagCG

ggtttttCGCGgtgtggttCGCGtagtCGgggttagCGgCGgCGtttgga

gtCGtgttttCGgtataattttttCGttCGgtagtCGgCGagttgtttat

atagttttatttgtgttatagttttatttagtagaaatttttgCGCGgtt

agggtaatttaatttaggggtCGaggCGgaggagggggCGgtgaggttgg

ttgggttggagaCGgaggtgtgCGCGttttaatattCGggattttttttt

tgtttttttttttattatagggaaggaaatatggatatggttCGggtgat

agaaatggggattCGgggttaaggaggaaggaggttagtttttaCGgttC

GgtttttCGgttttttttttttgaataggCGttggggattttCGCGgCGg

ttatagttggaCGCGCGtCGCGaaaggCGCGgCGgtttCGCGggCGttCG

gCGtgggttagagaggtCGgttttggtttggtttaggtCGttttCGtttC

GtttttattttgttttagtCGttttttataatttttatatCGaaggtaaa

gtaagtaaggaaagaagtCGgCGtttttttaagCGgattttttttttCGC

GtagtttCGgttatttttttagagCGtttCGgtgCGtattgCGgCGggCG

tagggaatCGaattaaaCGtttttgggCGgttattgtttttttttaattg

ttttaataatggtttttattaaaatggagatatttaattaaaggtttCGt

CGggtCGttttCGgttgtCGgtaaagttttggttttCGgtttgCGaCGtg

**tagatCGCGgggtCGCGgattCGgttggtgCGgggtattggCGtCGgttt**

**CGatatttttggtttttattttttttttagatttatagCGatagCGgtCG**

**Promoter 2**

**ttttatatCGtttttattgatCGatCGtagtttagtaagattgatCGagt**

**tggaatgggaagggatttttttttttttaggtttagttCGttagggtttC**

**GggtCGtgttgtagtttttggtttttggtgtCGtttttCGttttttagtt**

**tCGtagattttCG**gttttttttttgtttgCGCGgtCGggatCGtttaggt

aggCGtCGgggtttCGgggtttCGgggggagggattCGgCGgttCGgttt

ggtttCGttttttttttttgtttgtaaatatttgttgtttCGttggaaat

tCGaCGatttCGCGCGCGttttgtttgtaaagtttttaagtaaataCGtt

taaatgatCGtttCGggCGgttCGaggtaCGtttttttttttttCGCGgg

attagtaattttaggatttCGatttCGgggtttCGttttgtttgttattt

aggtCGggtagCGCGCGggCGttCGgggtCGtttttttCGgtatttCGgt

ttCGCGgagttCGtttggaagCGtCGgttgtttggttttggtgggtggtt

CGgtCGCGagtattttCGCGttttgggtttgtagttttagggttgagttt

tagaaagtCGCGttttCGttttgCGttatggtttttgttttttgtattCG

attgggtttttttatttttattgggatagggttttttCGtttttgggagg

gCGggtgggagggatttgtaCGtgggtatgtgtttgggggtgtgtgtggt

agtttaatttttatCGagtggagaaagttCGgttttattttttatttttt

ttttaagaggatatatatatttCGtttaatttgaCGtttaaagtagtCGt

ttttaggtttggggtttCGgataggttaagtCGgttgttgtCGattCGta

CGtCGtttCGggtttCGgtttttttttaggCGgtggtCGCGtCGtCGttt

gttCGgttgCGggatgtttgtttttaaggttttaggCGtttaggttgatC

GttagttttttattttggtgagggaggttttgCGttttgtagaagtttCG

ggttttgCGgCGgCGgCGgCGgtatttttttggtCGttttttCGtttttt

tttattCGagtttCGtCGgttgttttttgggaggaaggggattCGttaga

ttaggtCGaggtttaggtatttgggagggggtCGCGttaggtttgCGgga

aggCGtttaggttttttCGtaggaggttaggattagttttgtggCGgtgg

**tatagggagaattaggattattttttttttttattttCGgttaggatttt**

**Promoter 3**

**tttttttttttttCGttCGttaagggaggaggaagttCGgtttgtttttg**

**tCGgCGaggCGggttaggtCGgCGtttggtttggttCGgattttCGtagt**

**tCGgtttttCGttttaggttaCGCGtCGtagtttttttagtttCGttttt**

**ttttttttttttttttCGtgggtttCGaggtCGatttCGgtttttttttt**

tCGgtattttttttttatttttttttttttttCGttgttttgtgtttCGg

gtttCGgggttgggttCGtgaCGttaaatttagtgtggCGtCGgCGagat

tggtCGgCGCGggttattaaaagagtaaCGtttttttttttaattattta

ttgttagttCGggaattggggCGggtCGgttgggaattaaatgttaaata

ttttttatCGgttggttttattattCGggatatttagttttaggtttttt

atttttttttgttagttttaaattatttagattaagaagggttttCGaga

ttttttatttttttaaaggtgggggttgtttttttttattttCGgttagt

tCGatttggtgttCGCGattgaatttaaaCGaataatttttattttttta

tttaaaattagCGgataggCGtttttgtatCGgaaggtttttttattagt

tttttttttttgtttttgaaataatttttagttttagaagttagtatttt

taagatttggtattCGtagCGtttttgaCGCGgggattttttttttCGgt

tttgaggttttCGCGCGtagaatttatggtagtttttggttttaCGCGtg

gttgtgagttgggtttaCGttgCGtggCGtatttatttttCGattgaCGC

GCGgagaagtattttttatttttttttttagtttgtatttttaaatttta

atttCGggttttttagtaaaaatgCGtgtgtattCGtttttttttgttta

tggagaggttttCGgtgaatgttagatagtaa**ttaaataattaaaaggtt**

**tagagagCGttgtgagtaggagaagatgCGggtagtttggttggtttagg**

**Promoter 4**

**agagaCGagtggttagagaatgaaaggaaaatCGatgttttttaattggg**

**tCGtttaataaCGggagattaagtgggtttaggagaaaCGtggCGggggg**

**ggggggagttttttttaaaaaattggggttttttttttttgttttatata**

**tggaaatttagttttgattttaaataaatattagatttaagaaggtgagg**

attttaaagaagatttaaaCGCGagattaggaagatttaCGtttttatta

ttttttttaatatattttattaatttttttatagggatagtgttttaaaa

taCGaCGatttttatatattaaggtgaagttgaatattgttgttgttttt

ttatCGtatgaaaggaattttttagaatttatttttagggaCGgtttttt

ttatttCGaatttttgtatttgtagggaattttatggggtagaggagCGa

agagggaataatgtttttaatttaaataaaattttaaattaagtttttag

ttttatCGgtgggatagtttgCGggggaatttatt**tttttttttggtagt**

**attgttttgggggCGagtagagagtggatttggagtttttttttttCGtt**

**Promoter 5**

**tgatttttgttCGttCGagtagtattttCGggttagCGgttgtagttatt**

**CGggCGtCGttaggggatgtagtggtttaaaggatCGagCGggCGgtgta**

**ggttggaattCGCGgggCGgattaatCGCGgttCGgttatagtttCGttC**

**GttgattggtttttttaggtttCGttttCGttCGtttCGtttttttCGtt**

ggggCGtttCGgagtCGCGtgtttttCGtttCGgggtgtttattgCGtag

agCGtggtttggagattCGCGagtCGgggaaggtCGtCGtggagtttCGa

ttagaggtCGgggttggggtCGgtgtagatCGagggttggtttttttgat

tgtgggagaaaCGtCGggagtCGgagtaagtagggtttCGggCGgggCGa

aaggaaaagttgggtttttagagtgaagatCGagttttttttgttCGttt

atattgagCGtattCGgggaatgagttagagttagttttttttttttttt

ttttttttttattttttattgttgttatttaagttaaaagtatatattga

ttataaatattaggtttggaaagggtagttgtaatagttgaagCGtgttt

attttgggggtttgagagCGtagaaggttCGggaaagaggtgataatgat

aataaaattgaCGCGgaCGttttagttaaaggtattttttttttattCGa

tgatttatttttttaggaagtCGgttCGagaggtaaattttaaaatattt

tgatatgaaatattttgtttttttgattaatttaaCGCGtaCGttttttt

atatCGatgCGtttttttaaatattttgtattagattttaataatgattt

atgCGtgtttattttttaaaagtttgaaaaagaaa**attttgtttatCGaa**

**atgaattttatgaatggggtaggttggttgtatCGggaCGgaatCGttta**

**Promoter 6**

**ttCGattCGaatgaattggtaggagtCGCGgttatatttaaagggttaga**

**gCGCGCGttttttttCGttCGtttttaagtttCGCGggtttCGtttattt**

**tgttCGtCGttttttCGtCGgCGgtCGttttttgCGgCGttttttttCGg**

**ttagtggaggggCGggagg**aggggCGggggtgCGCGgggCGgggggagaa

gttttggagCGggtttgggttgtagtttttttgtgtCGgggattttgttt

tttattCGttagCGttaggtttttttttttCGgCGCGgatgatattagaa

tttttttaagttgCGtCGCGttatagttgtttgCGaatattgagttgttt

ggCGtCGttttgatatttttagaaagaatgtattttttgtaaaaaaaaaa

**5’ UTR (Noncoding exon)**

aaaaaatattgagagagggagagagagagagaagaagagagagagaCGga

gggagagCGagatagagCGagtaaCGtaatttgatCGagtaggtCGtaCG

tCGtCGttttttttttttttttgtttttCGttatttaggttggtattggt

gattttttttttttttaagtttgattttttgtttttaattatttgggagg

atttaaattaattttaaaaatttaattttttttttttggaggttttttag

gggttgagaggaCGgtttCGggatCGgtgttttCGagggagggatttgtt

ttttaagtCGtaatagttagttttgggatttgttttttaagttgtttagt

tagtttCGgttttCGCGagtCGggttgtagggaCGttttCGagagttttg

CGggtttCGCGgtCGtgttttCGCGttttCGtgCGggtttCGggtgCGtt

gggCGggCGggCGgCGCGaggggaggttgtgttattttagtaatttaggg

gtttatttaggttttttatttttttttttgtaggtgattCGaggagggat

ttCGttttCGagCGgttgaggatttCGgtgtagaggagtttggttCGtag

aattgtagagtCGtCGtttttttttataatttggtttCGttttattttgt

CGtatttagtttttggatttttgtttttttttttttttttttgttaaaCG

atttttttaagataatttttaaaaaatttttttttttgtttatttttgtt

ttttagtttttttatttttttatCGaaagtaaattatttaaCGattttCG

atttttCGaCGgtaggagtttttCGattttttaggCGgatCGtttttttt

tttCGCGCGCGggtttCGggttCGgCGagagggCGCGagtatagtCGagg

**First coding exon**

ttatggaggtgaCGgCGgattagtCGCGttggg**tgagttattattatttC**

**GtCGtgtttaaCGggtagtattCGgataCGtattattCGggttttagtta**

**tttttatatggaCGCGgCGtagtattCGttgtCGgaggaggtggatgtgt**

**Promoter 7**

**tttttaatatCGaCGgttaaggtaattaCGtttCGttttattaCGgaaat**

**tCGgttagggttaCGgtgtagaggtatttttCGatttattaCGgtgagtg**

**CGttCGgggtgtCGgggttttCGtCGgtCG**ttttagtCGtttCGgttCGg

ggaggtCGggagggatttgagggCGgggagaggttaagCGaaagttttta

tttgtCGtttttggtttatttataaaaaaattggggttCGgaaatgggCG

aggaaggtttttggttCGtCGCGagagtgtgttttgaaagagtCGtagta

ggCGtttttttCGgttggtggttttggggtttggCGtttatttggCGggC

GttaggtCGtagtttttCGtttttgCGCGgttgtaggttttggggtgggg

ggtttCGggatgtttttaagCGCGgggtgttttggttttgttttCGCGgg

ttCGggttttttgggttCGgtagtCGgCGtCGgataagtaCGgttggaat

tCGgtttttttaaaaaatttggCGttttttgttattCGttagtttttttt

aggCGggtgggCGgggtggagggggttttttgttagCGttttttaattCG

tatatttttagaaaggttttagagatttatttatttatattttttttttt

gatttttttttagagttagtgtttttggtttttagataggttttttattt

**Bisulfite sequencing/ MS-HRM primers**

Primer set 1

GATA3 F1: 5’-agggagagagttgaaaagggtattt-3’ (TM=58)

GATA3 R1: 5’-TCGAAAAATCCTAACCCTCCC-3’ (TM= 58.1)

Amplicon size = 288 bp; 9 CpG sites

Location: -6,064, -5,776

Primer set 2

GATA3 F2: 5’- tCGgttgtAGgtaaagttttggtttT -3’ (TM=59.3)

GATA3 R2: 5’- CGAATCCCTCCCCCCTAAA-3’ (TM=59.7)

Amplicon size = 378 bp; 33 CpG sites

Location: -4,390, -4,012

Primer set 3

GATA3 F3: 5’- TCGtaggaggttaggattagttttgtg -3’ (TM=59.1)

GATA3 R3: 5’- CCGAAACCCTAAACACAAAACAA-3’ (TM=59)

Amplicon size = 341 bp; 24 CpG sites

Location: -3,034, -2,693

Primer set 4

GATA3 F4: 5’- ttgtgagtaggagaagatgAGgGTAG -3’ (TM=58.1)

GATA3 R4: 5’- CGTTTAAATCTTCTTTAAAATCCTCACC-3’ (TM=59)

Amplicon size = 262 bp; 6 CpG sites

Location: -1,941, -1,679

Primer set 5

GATA3 F5: 5’- ggtagaggagAGaagagggaataatG -3’ (TM=58)

GATA3 R5: 5’- GCGAATCTCCAAACCACTCTCTA-3’ (TM=58.5)

Amplicon size = 433 bp; 34 CpG sites

Location: -1,463, -1,030

Primer set 6

GATA3 F6: 5’- atgaatggggtaggttggttgtat -3’ (TM=58.5)

GATA3 R6: 5’- CTCCTCCCTCCCCTCCACT-3’ (TM=59)

Amplicon size = 212 bp; 24 CpG sites

Location: -391, -179

Primer set 7

GATA3 F7: 5’- tagtggaggggAGggaggag-3’ (TM=58.5)

GATA3 R7: 5’- CGTCCTCTCAACCCCTAAAAAAC-3’ (TM=59)

Amplicon size = 514 bp; 27 CpG sites

Location: -199, +314

Primer set 8

GATA3 F8: 5’- TTTgtaggtgattAGaggagggattt-3’ (TM=58.6)

GATA3 R8: 5’- CCGTCACCTCCATAACCTCTACTATAC-3’ (TM=58.6)

Amplicon size = 389 bp; 27 CpG sites

Location: +576, +965

Primer set 9

GATA3 F9: 5’- GCGagtatagtAGaggttatggaggtg-3’ (TM=58.8)

GATA3 R9: 5’- CCTCAAATCCCTCCCTACCTCC-3’ (TM=60.9)

Amplicon size = 338 bp; 32 CpG sites

Location: +934, +1272

**REFERENCES**

1. Wang Y and FC Leung, *An evaluation of new criteria for CpG islands in the human genome as gene markers.* Bioinformatics, 2004. **20**(7): p. 1170-7.

2. Irizarry RA, C Ladd-Acosta, B Wen, et al., *The human colon cancer methylome shows similar hypo- and hypermethylation at conserved tissue-specific CpG island shores.* Nat Genet, 2009. **41**(2): p. 178-86.

3. Prestridge DS, *Predicting Pol II promoter sequences using transcription factor binding sites.* J Mol Biol, 1995. **249**(5): p. 923-32.

4. Wojdacz TK, T Borgbo, and LL Hansen, *Primer design versus PCR bias in methylation independent PCR amplifications.* Epigenetics, 2009. **4**(4): p. 231-4.

5. Wojdacz TK, A Dobrovic, and LL Hansen, *Methylation-sensitive high-resolution melting.* Nat Protoc, 2008. **3**(12): p. 1903-8.
