# Supplementary material for: 5-AZA-dC induces epigenetic changes associated with modified glycosylation of secreted glycoproteins and increased EMT and migration in chemo-sensitive cancer cells
Source: Clin Epigenetics. 2021 Feb 12;13:34. doi: 10.1186/s13148-021-01015-7 (PMC7881483; doi:10.1186/s13148-021-01015-7)
Supplement: Supplementary file 5 — Additional file 5: Figure S1: 5-AZA-dC treatment increases resistance to cisplatin in chemo-sensitive and decreases in chemo-resistant cell lines. % viability of A2780 and A2780cis cells treated with 1 μM 5-AZA-dC and 1 μM 5-AZA-dC in combination with 1 μM cisplatin. * = P-value ≤ 0.05 or ** = P-value ≤ 0.005 (T-test). Figure S2: Glycosylation changes of cell glycans on A2780 and A2780cis chemo-sensitive/chemo-resistant pair. (A) Representative UPLC chromatograms produced from secreted N-glycans of ovarian chemo-sensitive- chemo-resistant pair (A2780, A2780cis) and their separation into 39 peaks. (B) Plotted peak areas from the cell N-glycans of these cell lines. The glycans in each peak (GP1-GP39) and features are listed in Table S2. Significant changes (p < 0.05) are starred: * = P-value ≤ 0.05 or ** = P-value ≤ 0.005. (MANOVA). Heatmap histograms indicating fold changes in 5-AZA-dC treated compared to untreated cells were created using Hierarchial Clustering Explorer HCE 3.5 software. Blue indicates decreases, and red indicates increases. The shade of colour corresponds to amounts of the decreases/increases. Figure S3: Secreted glycans differ in chemo-resistant comparing to chemo-sensitive cell lines. Plotted peak areas of GP13 and 14 from the secreted N-glycans of chemo-sensitive and chemo-resistant ovarian cancer cell lines. Significant changes (p < 0.05) are starred: * = P-value ≤ 0.05 or ** = P-value ≤ 0.005. (MANOVA). Figure S4. Representative Western blots of EMT markers in the 4 ovarian cancer cell lines and 2 TNBC cell lines post-5-AZA-dC treatment (T) compared to non-treated (UT) controls. Figure S5: Migration results are not attributable to proliferation. Migration (A) and proliferation (B) or the representative 1 uM 5-AZA-dC treated relative to the untreated A2780cis cells. Figure S6. Western blot analyses of the senescence markers p16, p21 and Rb and of cellular apoptosis (PARP cleavage) markers, post-5-AZA-dC treatment. Representative Western blots o [file 13148_2021_1015_MOESM5_ESM.pptx]

## Slide 1
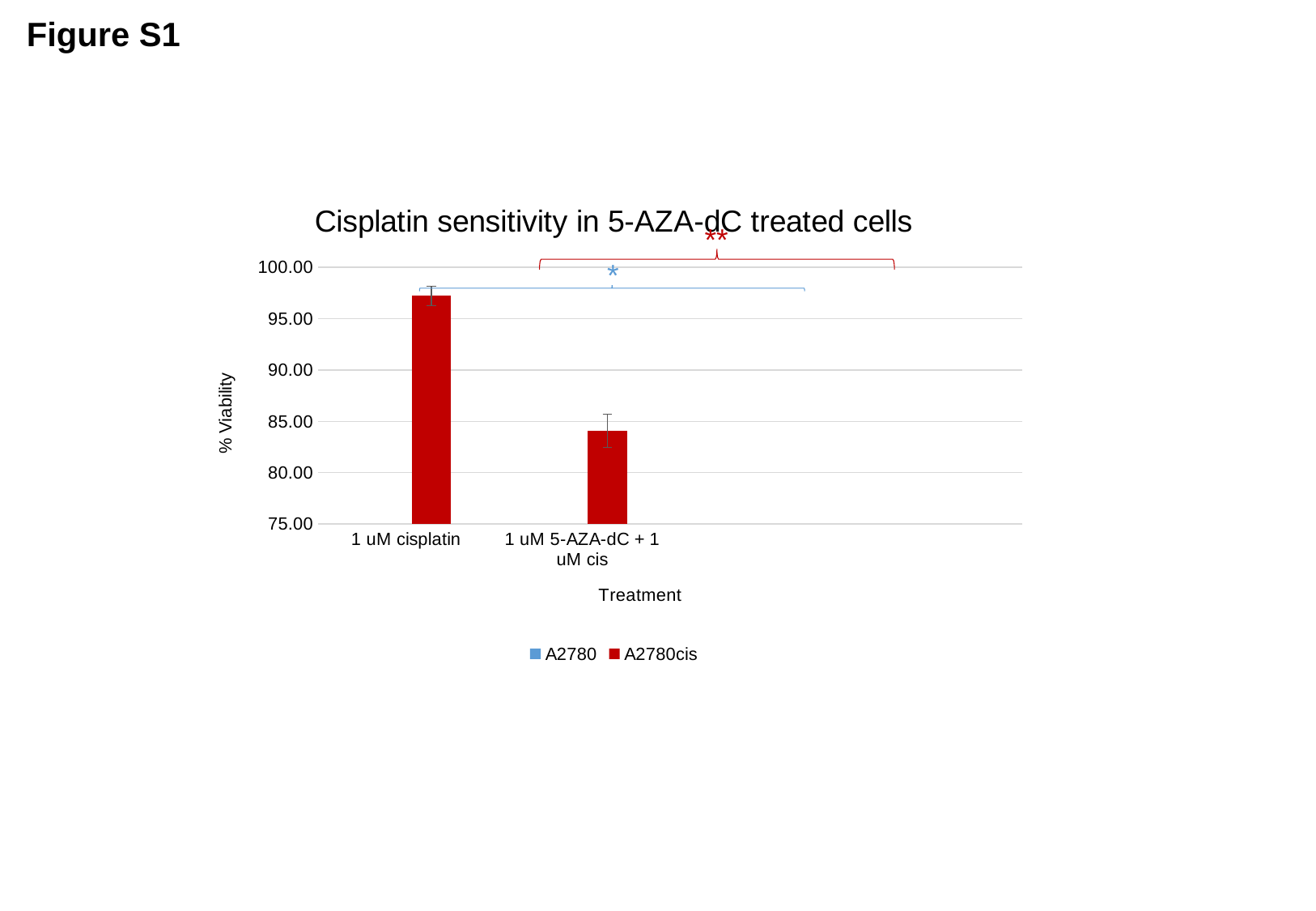

Figure S1
### Chart: Cisplatin sensitivity in 5-AZA-dC treated cells
| Category | A2780 | A2780cis |
|---|---|---|
| 1 uM cisplatin | 80.87 | 97.19999999999999 |
| 1 uM 5-AZA-dC + 1 uM cis | 89.03333333333335 | 84.06666666666666 |**
*

## Slide 2
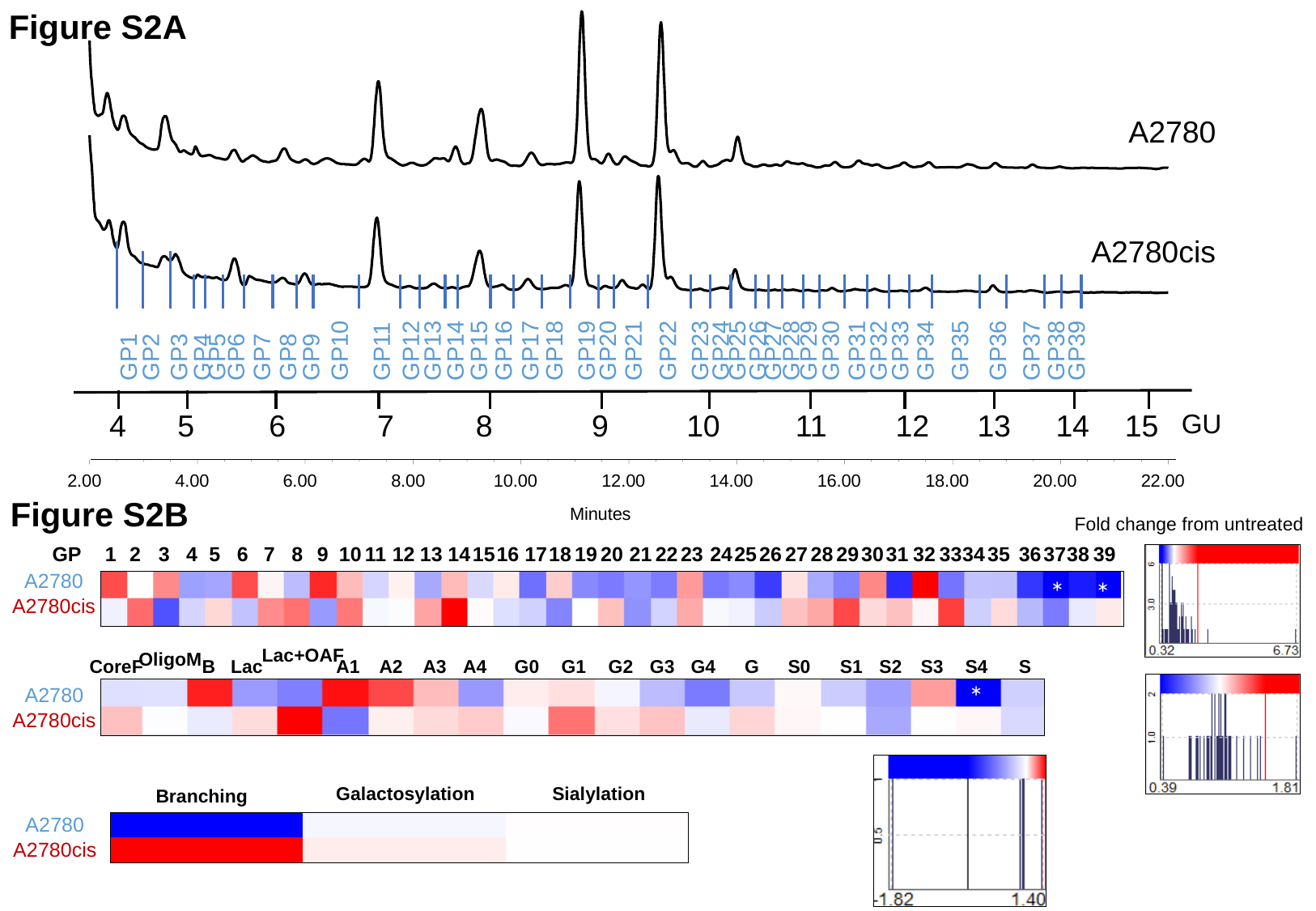

Figure S2A
A2780
A2780cis
GP10
GP12
GP13
GP14
GP15
GP16
GP17
GP18
GP19
GP20
GP21
GP22
GP23
GP24
GP25
GP26
GP27
GP28
GP29
GP30
GP31
GP32
GP33
GP34
GP35
GP36
GP37
GP38
GP39
GP11
GP1
GP2
GP3
GP4
GP5
GP6
GP7
GP8
GP9
4
5
6
7
8
9
10
11
12
13
14
15
GU
2.00
4.00
6.00
8.00
10.00
12.00
14.00
16.00
18.00
20.00
22.00
Figure S2B
Minutes
Fold change from untreated
GP
1
2
3
4
5
6
7
8
9
10
11
12
13
14
15
16
17
18
19
20
21
22
23
24
25
26
27
28
29
30
31
32
33
34
35
36
37
38
39
A2780
*
*
A2780cis
Lac+OAF
OligoM
CoreF
B
Lac
A1
A2
A3
A4
G0
G1
G2
G3
G4
G
S0
S1
S2
S3
S4
S
*
A2780
A2780cis
Galactosylation
Sialylation
Branching
A2780
A2780cis

## Slide 3
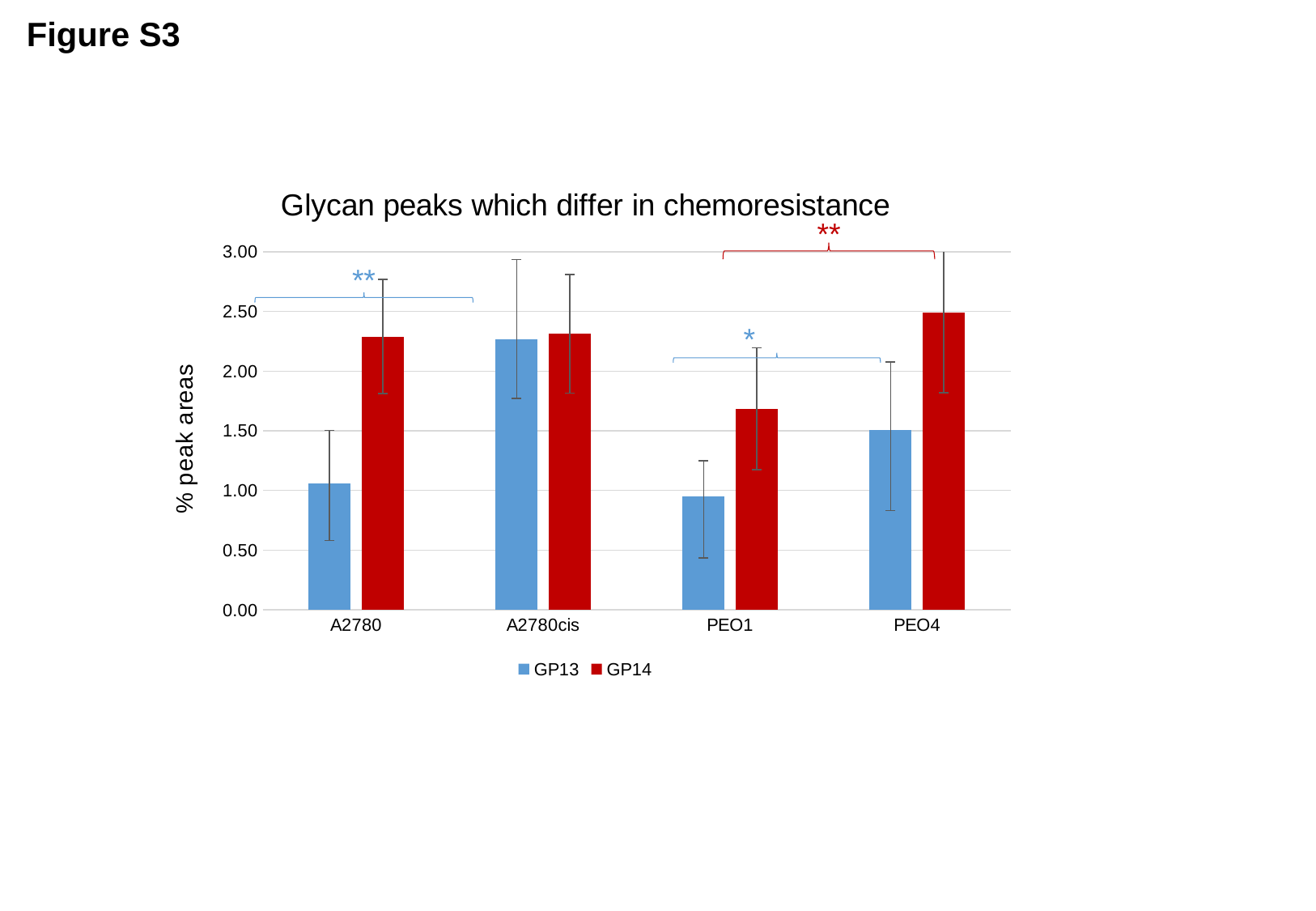

Figure S3
### Chart: Glycan peaks which differ in chemoresistance
| Category | GP13 | GP14 |
|---|---|---|
| A2780 | 1.06 | 2.29 |
| A2780cis | 2.27 | 2.3125 |
| PEO1 | 0.9475000000000001 | 1.6841666666666668 |
| PEO4 | 1.5038461538461543 | 2.491538461538461 |
**
**
*

## Slide 4
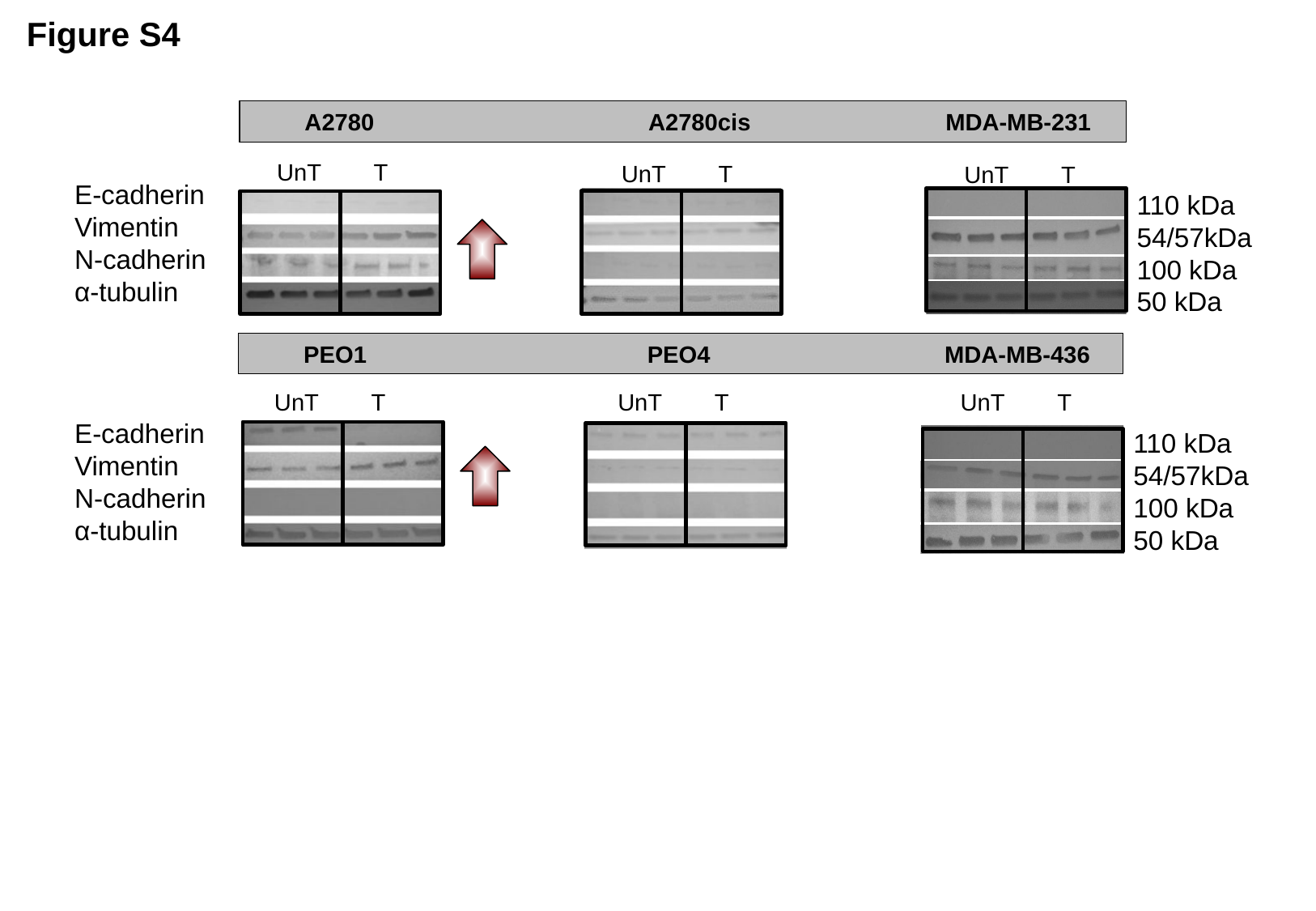

Figure S4
A2780
A2780cis
MDA-MB-231
UnT T
UnT T
UnT T
E-cadherin
Vimentin
N-cadherin
α-tubulin
PEO1
PEO4
MDA-MB-436
UnT T
UnT T
UnT T
E-cadherin
Vimentin
N-cadherin
α-tubulin
110 kDa
54/57kDa
100 kDa
50 kDa
110 kDa
54/57kDa
100 kDa
50 kDa

## Slide 5
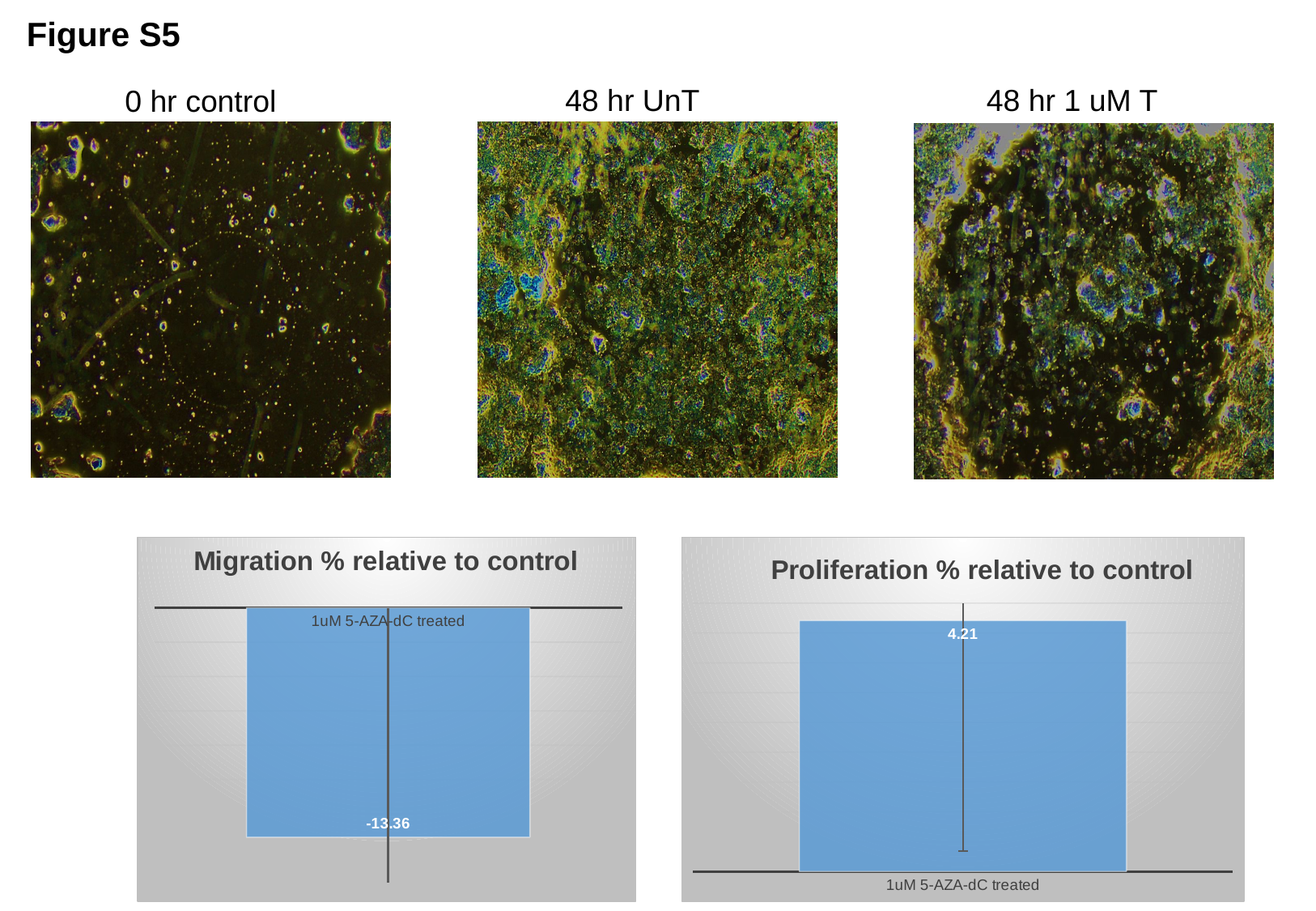

Figure S5
48 hr UnT
48 hr 1 uM T
0 hr control
### Chart:
| Category | Proliferation % relative to control |
|---|---|
| 1uM 5-AZA-dC treated | 4.209493426936637 |
### Chart:
| Category | Migration % relative to control |
|---|---|
| 1uM 5-AZA-dC treated | -13.3586115402708 |

## Slide 6
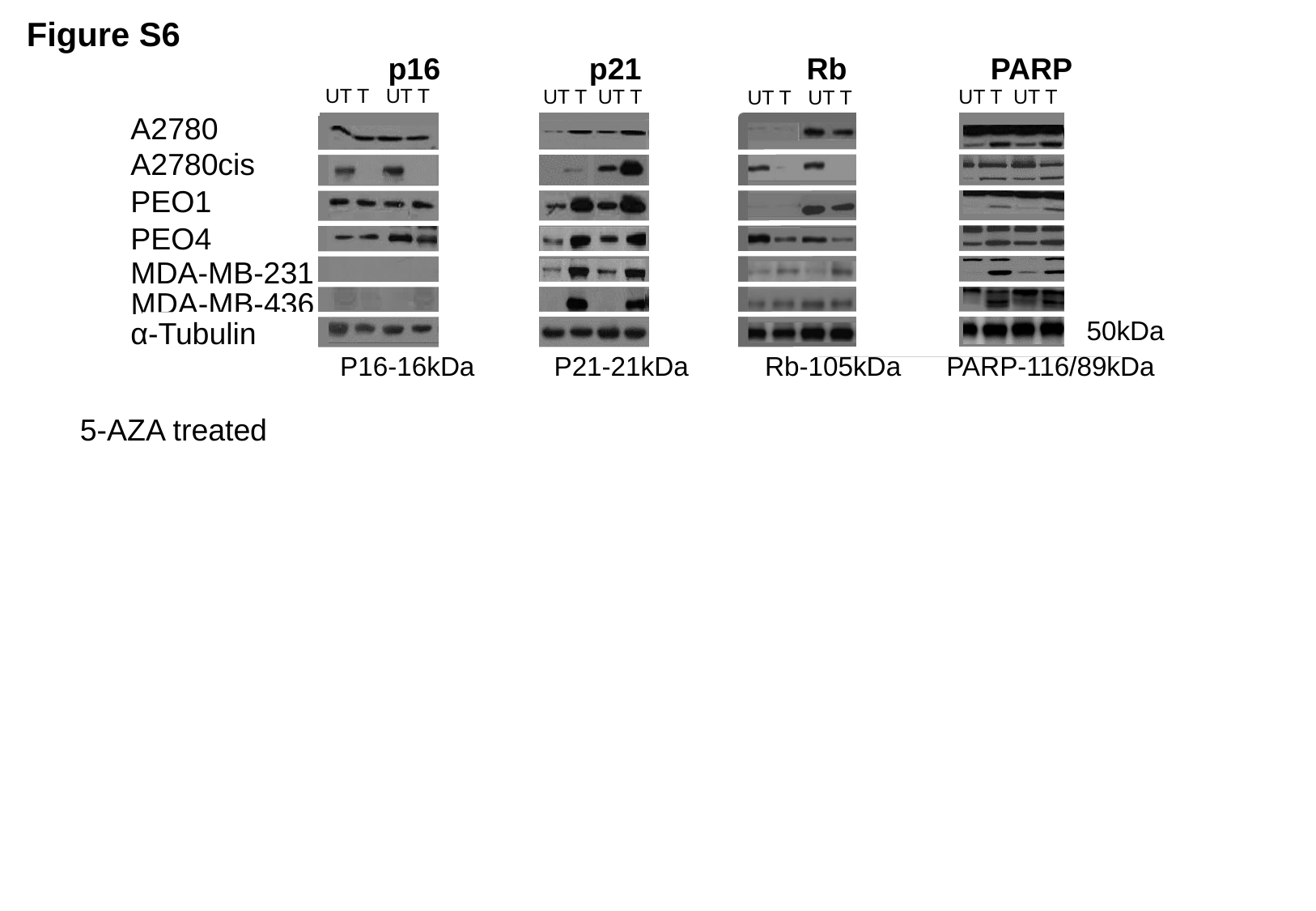

Figure S6
p16
p21
Rb
PARP
v
v
UT T UT T UT T
UT T UT T UT T
UT T UT T UT T
UT T UT T UT T
v
v
A2780
A2780cis
PEO1
PEO4
MDA-MB-231
MDA-MB-436
50kDa
α-Tubulin
P16-16kDa
P21-21kDa
Rb-105kDa
PARP-116/89kDa
5-AZA treated

## Slide 7
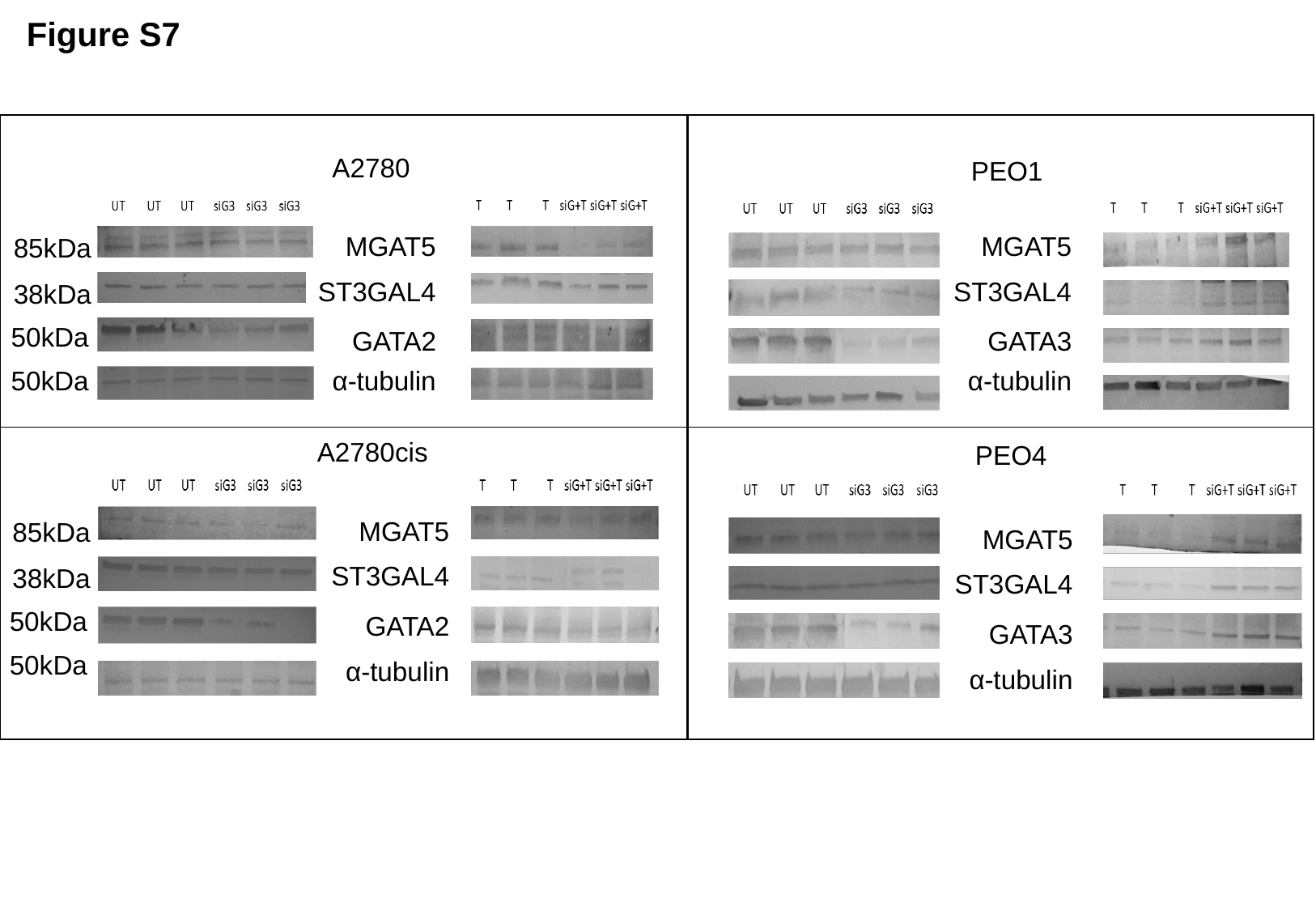

Figure S7
A2780
PEO1
MGAT5
MGAT5
85kDa
ST3GAL4
ST3GAL4
38kDa
50kDa
GATA2
GATA3
50kDa
α-tubulin
α-tubulin
A2780cis
PEO4
MGAT5
85kDa
MGAT5
ST3GAL4
38kDa
ST3GAL4
50kDa
GATA2
GATA3
50kDa
α-tubulin
α-tubulin
